# Anti-inflammatory Limonoids From *Cortex Dictamni*

**DOI:** 10.3389/fchem.2020.00073

**Published:** 2020-02-28

**Authors:** Yue Chen, Jingya Ruan, Fan Sun, Huimei Wang, Shengcai Yang, Ying Zhang, Jiejing Yan, Haiyang Yu, Yuanqiang Guo, Yi Zhang, Tao Wang

**Affiliations:** ^1^Tianjin Key Laboratory of TCM Chemistry and Analysis, Tianjin University of Traditional Chinese Medicine, Tianjin, China; ^2^Institute of TCM, Tianjin University of Traditional Chinese Medicine, Tianjin, China; ^3^State Key Laboratory of Medicinal Chemical Biology, College of Pharmacy, Nankai University, Tianjin, China; ^4^Tianjin Key Laboratory of Molecular Drug Research, Nankai University, Tianjin, China

**Keywords:** *Cortex Dictamni*, dictamlimonoside, dictamlimonol, tumor necrosis factor, interleukin-6, inducible nitric oxide synthase, nuclear factor kappa-light-chain-enhancer of activated B cells, cyclooxygenase-2

## Abstract

The root barks of perennial herb *Dictamnus dasycarpus* (*Cortex Dictamni*) were reported to be rich in anti-inflammation activity constituents, limonoids. Then, the investigation of anti-inflammation therapeutic limonoids from this plant was developed in the present study. Through the combination of various chromatographies isolation, six new limonoids, named dictamlimonol A (**1**), dictamlimonoside B (**2**), and dictamlimonols C–F (**3**–**6**), along with seven known ones (**7**–**13**), were obtained. Their structures were ascertained based on the extensive spectroscopic methods and ECD data analysis. Among them, compound **1** was the first 7,19-epoxy limonoid found in natural products. The anti-inflammatory effects of all limonoids were evaluated in lipopolysaccharide (LPS)-treated RAW 264.7 cell lines. Compounds **5**, **7**–**11**, and **13** were found to inhibit LPS-induced nitric oxide (NO) production. Moreover, dictamlimonol D (**5**), fraxinellone (**11**), and dasylactone A (**13**) were found to reduce the LPS-induced expressions of interleukin-6 (IL-6), tumor necrosis factor (TNF-α), inducible nitric oxide synthase (iNOS), nuclear factor kappa-light-chain-enhancer of activated B cells (NF-κB), and cyclooxygenase-2 (COX-2) at the protein levels in a dose-dependent manner. These findings support that the administration of *Cortex Dictamni* may be beneficial for inflammation.

## Introduction

Limonoids are highly oxygenated tetranorterpenoids derived from a precursor with a 4,4,8-trimethyl-17-furanyl steroid skeleton (Lv et al., 2015). Until now, they are reported to distribute mainly in Rutaceae and Meliaceae plant resources. Previous studies suggested that limonoids exhibited strong anti-inflammatory activity (Akihisa et al., 2017; Hu et al., 2018; Sun et al., 2018).

The natural plants of *Dictamnus* genus (Rutaceae family) are main resources of limonoids. The limonoids obtained from them could be subdivided as limonoid aglycones, degraded limonoids, and limonoid glucosides (Lv et al., 2015). *Dictamnus dasycarpus* Turcz is a perennial herb belonging to *Dictamnus* genus, widely distributed in China. Its root barks (*Cortex Dictamni*) have been used to treat inflammation, scabies, rheumatic pain, jaundice, and other symptoms (Yang et al., 2017, 2018). Though limonoids were reported as one of the major constituents in *Cortex Dictamni*, only fraxinellone obtained from it has been proved to possess *in vitro* anti-inflammatory effect (Kim et al., 2009; Lee et al., 2009; Wu et al., 2014) to date.

This study aims to clarify anti-inflammation limonoids in *Cortex Dictamni* by phytochemistry and bioactivity screening. Multiple chromatographies and spectral techniques were combined to isolate and identify limonoids. Then, the inhibitory activities of all obtained limonoids against nitric oxide (NO) production in RAW 264.7 cell lines induced by lipopolysaccharide (LPS) were evaluated. Furthermore, the anti-inflammatory mechanism of activity compounds was studied by using Western blot assay.

## Results and Discussion

### Identification of New Compounds

**Figure 1:**
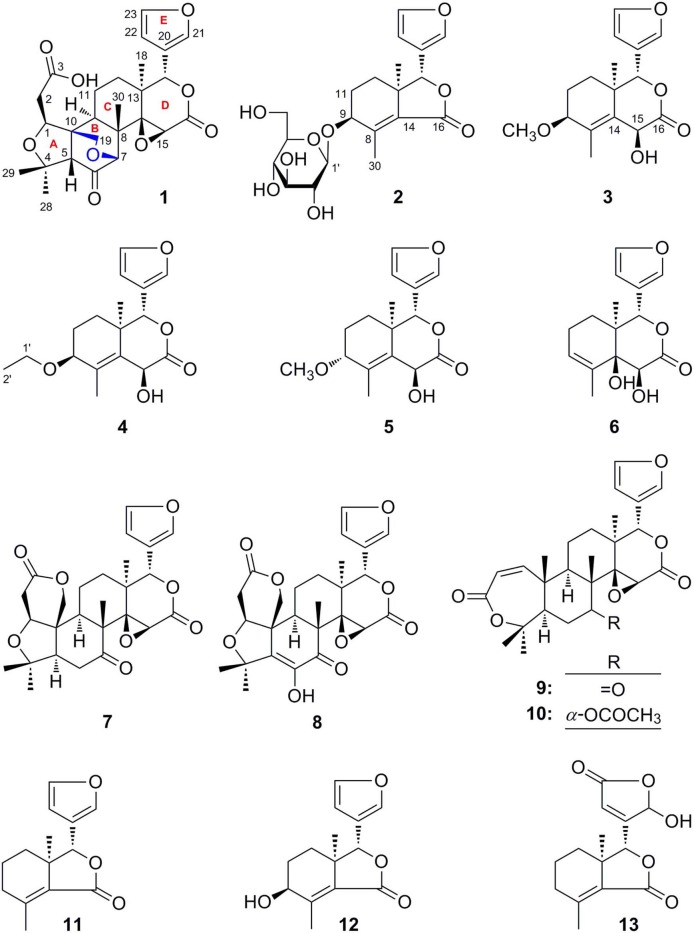
The structures of compounds **1**–**13** isolated from *Cortex Dictamni*.

*Cortex Dictamni* was extracted by 70% ethanol–water and then partitioned in EtOAc-H_2_O to afford EtOAc and H_2_O layer extract, respectively. The H_2_O layer extract was eluted with H_2_O and 95% EtOH, successively. After subjecting to D101 macroporous resin column chromatography (CC), the silica gel, ODS, Sephadex LH-20 CC, and preparative high-performance liquid chromatography (pHPLC) were used to isolate the 95% EtOH eluate from D101 macroporous resin CC. As a result, six new limonoids, dictamlimonol A (**1**), dictamlimonoside B (**2**), and dictamlimonols C–F (**3**–**6**), as well as seven known ones, limonin (**7**) (Guo, 2011), limonin diosphenol (**8**) (Du et al., 2005), obacunon (**9**) (Dong et al., 2010), 7α-obacunyl acetate (**10**) (Bennett and Hasegawa, 1982), fraxinellone (**11**) (Wang et al., 2006), 9β-hydroxyfraxinellone (**12**) (D'Ambrosio and Guerriero, 2002), and dasylactone A (**13**) (Yang et al., 2011), were obtained (Figure 1).

Dictamlimonol A (**1**) was obtained as an optically active [[α]_D_^25^ −100.0, MeOH] white powder. The molecular formula, C_26_H_30_O_9_, of **1** was established by negative-ion ESI-Q-Orbitrap MS analysis [*m/z* 485.17969 [M–H]^−^; calcd for C_26_H_29_O_9_, 485.18061]. Its ^1^H, ^13^C NMR (Table 1, C_5_D_5_N), ^1^H ^1^H COZY, HSQC, and HMBC spectra indicated the presence of one furan ring at δ_H_ 6.60 (1H, t like, *ca*. *J* = 1 Hz, H-22), 7.68 (1H, t like, *ca*. *J* = 1 Hz, H-23), 7.77 (1H, t like, *ca*. *J* = 1 Hz, H-21), and four methyl groups at δ_H_ 0.91, 1.24, 1.40, 1.48 (3H each, all s, H_3_-30, 28, 18, 29). According to the chemical shifts and the correlations observed in its ^1^H ^1^H COZY spectrum, “–O–CH–CH_2_-,” “–CH_2_-CH_2_-,” and “–CH=CH–O–” moieties were deduced. The planar structure of it was determined based on the key HMBC correlations from H-1 to C-3–5; H_2_-2 to C-3, C-10; H-5 to C-6; H-7 to C-6, C-9, C-14; H-15 to C-16; H-17 to C-14, C-20–22; H_3_-18 to C-12–14, C-17; H_2_-19 to C-1, C-5, C-7, C-10; H_3_-28 to C-4, C-5, C-29; H_3_-29 to C-4, C-5, C-28; H_3_-30 to C-7–9, C-14, which was a derivative of limonin (**7**). The relative configurations of C-13 and C-17 of **1** should be the same as those of **7** on the basis of biogenetic consideration, which indicated both 13-methyl and 17-furan ring presented α-orientations. Meanwhile, the NOE correlations between δ_H_ 1.40 (H_3_-18) and δ_H_ 2.89 (H-9), 4.72 (H-15); δ_H_ 2.89 (H-9); and δ_H_ 1.24 (H_3_-28), 4.61 (H-1), 4.72 (H-7); δ_H_ 1.24 (H_3_-28); and δ_H_ 4.61 (H-1) observed in its NOESY spectrum suggested that H-1, H-7, H-9, H-15, and H_3_-28 were α-oriented; on the other hand, δ_H_ 1.48 (H_3_-29) and δ_H_ 3.40 (H-5); δ_H_ 3.40 (H-5) and δ_H_ 4.62 (Hb-19); and δ_H_ 4.62 (Hb-19) and δ_H_ 0.91 (H_3_-30) (**Figure 3**) indicated that H-5, H_2_-19, H_3_-29, and H_3_-30 were β-oriented. Finally, the absolute configuration of **1** was clarified by comparison of experimental and calculated ECD data, which was recorded at the B3LYP/SVP level with the CPCM model (Frisch et al., 2009; Shi et al., 2019). The calculated ECD spectrum of **1** (**Figure 4A**) was consistent with the experimental data closely. The result indicated that the absolute configuration of **1** was 1*S*,5*S*,7*R*,8*S*,9*R*,10*R*,13*S*,14*R*,15*S*,17*S*. Thus, its structure was finally established and named dictamlimonol A. It was the first 7,19-epoxy limonoid found in natural products.

**Table 1:** ^1^H and ^13^C NMR data for **1** in C_5_D_5_N.

**No**.	**δ_**C**_**	**δ_**H**_ (*J* in Hz)**	**No**.	**δ_**C**_**	**δ_**H**_ (*J* in Hz)**
1	83.6	4.61 (dd, 2.5, 2.5)	13	38.2	
2	36.8	3.10 (dd, 2.5, 16.0)	14	66.7	
		3.19 (dd, 2.5, 16.0)	15	52.1	4.72 (s)
3	170.9		16	167.7	
4	82.5		17	78.4	5.76 (s)
5	65.1	3.40 (s)	18	21.2	1.40 (s)
6	208.0		19	70.6	4.50, 4.62 (both d, 12.5)
7	83.2	4.72 (s)	20	121.1	
8	46.6		21	142.0	7.77 (t like, *ca*. 1)
9	46.8	2.89 (dd, 4.5, 10.0)	22	110.7	6.60 (t like, *ca*. 1)
10	49.7		23	143.8	7.68 (t like, *ca*. 1)
11	20.3	1.91 (m)	28	24.2	1.24 (s)
12	32.3	1.47 (m, overlapped)	29	29.1	1.48 (s)
		2.01 (m)	30	15.3	0.91 (s)

**Figure 2:**
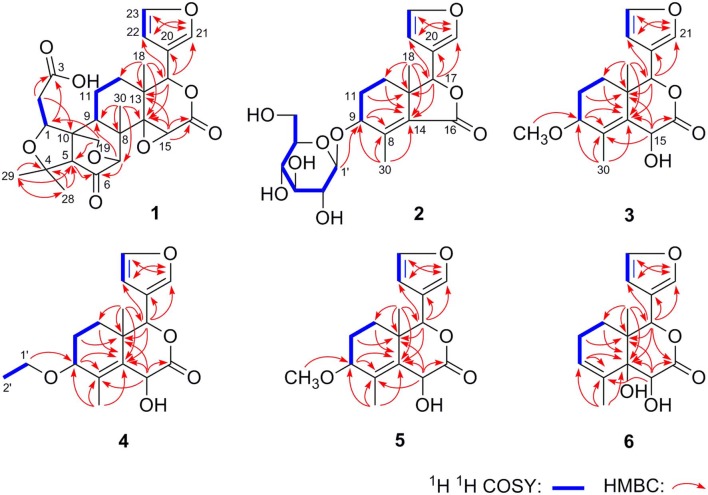
The main ^1^H ^1^H COZY and HMBC correlations of **1**–**6**.

**Figure 3:**
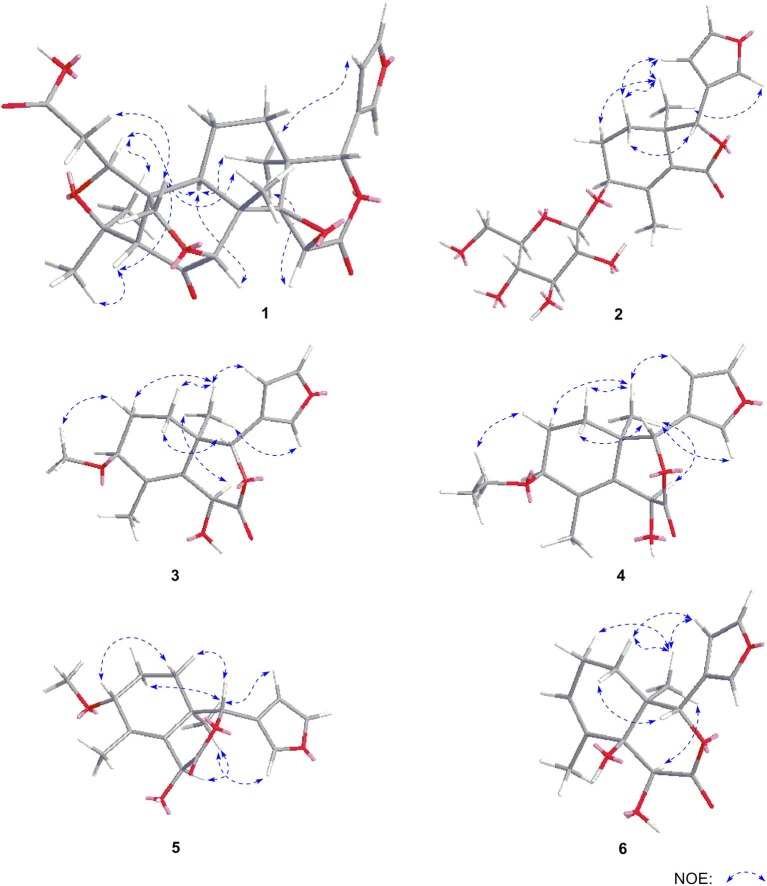
The main NOE correlations of **1**–**6**.

**Figure 4:**
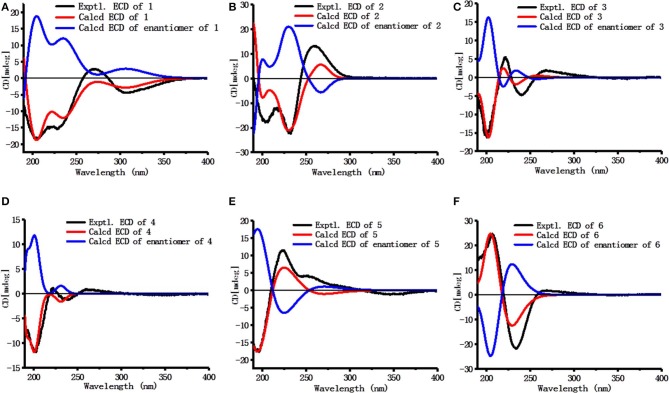
Calculated and experimental ECD spectra of **1**–**6 (A–F)** in acetonitrile.

Dictamlimonoside B (**2**) was a white powder with negative optical rotation [[α]_D_^25^ −72.1, MeOH]. Its molecular formula was determined to be C_20_H_26_O_9_ [*m/z* 455.15375 [M + COOH]^−^, calcd for C_21_H_27_O_11_, 455.15479] by negative-ion ESI-Q-Orbitrap MS. d-glucose was detected from its acid hydrolysis product by HPLC analysis (Zhang et al., 2015). The ^1^H, ^13^C NMR (Table 2, CD_3_OD) spectra showed signals of two methyl groups at δ_H_ 0.84, 2.25 (3H each, both s, H_3_-18 and 30), two methylene groups at δ_H_ [1.58 (1H, dt like, *ca*. *J* = 4, 13 Hz), 1.78 (1H, dt like, *ca*. *J* = 3, 13 Hz), H_2_-12], [1.91, 2.39 (1H each, both m, H_2_-11)], two oxygenated methylene groups at δ_H_ 4.11 (1H, br. d, *ca*. *J* = 4 Hz, H-9), 4.97 (1H, br. s, H-17), one furan ring at δ_H_ 6.44 (1H, br. s, H-22), 7.55 (2H, m, overlapped, H-21 and 23), one β-d-glucopyranosyl at δ_H_ 4.53 (1H, d, *J* = 7.5 Hz, H-1'), together with one α, β-unsaturated ketone group at δ_C_ 131.9 (C-14), 147.1 (C-8), 171.8 (C-16). The ^1^H ^1^H COZY spectrum of **2** suggested the presence of three partial structures written in bold lines (Figure 2). Moreover, the abovementioned three partial structures and relative functional groups were connected together by the long-range correlations from H-9 to C-8, C-14; H_2_-11 to C-8, C-13; H-17 to C-14, C-20–22; H_3_-18 to C-12–14, C-17; H_3_-30 to C-8, C-9, C-14, C-16; H-1' to C-9 displayed in the HMBC spectrum. Meanwhile, the NOE correlations (determined in DMSO-*d*_6_) (Figure 3) between δ_H_ 0.77 (H_3_-18) and δ_H_ 6.52 (H-22), 7.72 (H-21), as well as biogenetic law suggested that 13-methyl and 17-furan ring were α-oriented. The correlation between δ_H_ 0.77 (H_3_-18) and δ_H_ 1.80 (H-11α) indicated that both 13-methyl and the proton with signal at δ_H_ 1.80 were in axial bond. Moreover, the coupling constant of H-9 was about 4 Hz, which suggested that the proton was presented in equatorial bond (one α-H in compound **2**). Furthermore, the aglycone of it, 9β-hydroxyfraxinellone (**12**), was obtained when it was hydrolyzed with β-glucosidase. The calculated ECD spectrum of **2** (Figure 4B) matched the experimental data closely, which indicated that its absolute configuration was 9*S*,13*R*,17*R*. The structure of **2** was therefore clarified and named dictamlimonoside B.

**Table 2:** ^1^H and ^13^C NMR data for **2** in CD_3_OD and DMSO-*d*_6_.

	**in CD**_****3****_**OD**		**in DMSO-*****d***_****6****_	
**No**.	**δ_**C**_**	**δ_**H**_ (*J* in Hz)**	**δ_**C**_**	**δ_**H**_ (*J* in Hz)**
8	147.1		145.4	
9	78.1	4.11 (br. d, *ca*. 4)	75.9	4.01 (br. d, *ca*. 4)
11	27.4	1.91 (m)	25.7	1.80 (tt like, *ca*. 4, 14)
		2.39 (m)		2.27 (dt like, *ca*. 4, 14)
12	28.2	1.58 (dt like, *ca*. 4, 13)	26.6	1.50 (dt like, *ca*. 4, 13)
		1.78 (dt like, *ca*. 3, 13)		1.63 (dt like, *ca*. 2, 13)
13	44.6		42.7	
14	131.9		129.9	
16	171.8		169.0	
17	84.8	4.97 (br. s)	82.1	5.01 (br. s)
18	19.4	0.84 (s)	18.7	0.77 (s)
20	121.7		120.1	
21	141.4	7.55 (m, overlapped)	140.2	7.72 (m, overlapped)
22	109.8	6.44 (br. s)	109.0	6.52 (br. s)
23	145.0	7.55 (m, overlapped)	143.8	7.72 (m, overlapped)
30	15.9	2.25 (s)	15.0	2.18 (s)
1′	107.0	4.53 (d, 7.5)	105.8	4.40 (d, 8.0)
2′	75.5	3.21 (dd, 7.5, 8.5)	73.7	2.98 (m)
3′	78.0	3.38 (dd, 8.5, 8.5)	76.7	3.15 (m)
4′	71.5	3.31 (m, overlapped)	70.0	3.06 (m)
5′	77.9	3.31 (m, overlapped)	76.7	3.17 (m)
6′	62.7	3.69 (dd, 5.0, 12.0)	61.1	3.46 (m)
		3.88 (br. d, *ca*. 12)		3.69 (m)

Dictamlimonol C (**3**) was isolated as white powder with negative optical rotation ([α]_D_^25^ −74.6, MeOH). The molecular formula, C_16_H_20_O_5_, of **3** was established by negative-ion ESI-Q-Orbitrap MS [*m/z* 337.12790 [M + COOH]^−^, calcd for C_17_H_21_O_7_, 337.12818]. Comparing its ^1^H and ^13^C NMR spectroscopic data (Table 3, CDCl_3_) with those of **2** indicated that they possessed the same substituent groups [two methyl groups at δ_H_ 0.95, 1.90 (3H each, both s, H_3_-18 and 30), one furan ring at δ_H_ 6.40, 7.38, 7.45 (1H each, all t like, *ca*. *J* = 2 Hz, H-22, 23, 21), and a “—CH_2_–CH_2_–CH(O)—”moiety]. At the same time, there were one more oxygenated methine proton at δ_H_ 4.90 (1H, s, H-15) and one more methoxy at δ_H_ 3.41 (3H, s, 9-OCH_3_) in **3**. The long-range correlations from H-9 to C-8, C-14; H-15 to C-8, C-13, C-14, C-16; H-17 to C-14, C-20–22; H_3_-18 to C-12–14, C-17; H_3_-30 to C-8, C-9, C-14; 9-OCH_3_ to C-9 were observed in its HMBC spectrum, and its planar structure was elucidated. Moreover, the NOE correlations (Figure 3) between δ_H_ 0.95 (H_3_-18) and δ_H_ 4.90 (H-15), 6.40 (H-22), 7.45 (H-21) revealed that 13-methyl, 17-furan ring, as well as H-15 presented α-orientation, and the correlations between δ_H_ 0.95 (H_3_-18) and δ_H_ 1.73 (H-11α); δ_H_ 1.98 (H-11β); and δ_H_ 3.41 (9-OCH_3_), along with the coupling constant of H-9 (*ca*. *J* = 4 Hz), suggested that 9-OCH_3_ presented β-orientation. The calculated ECD spectrum of **3** (Figure 4C) was identical to the experimental one, which indicated that its absolute configuration was 9*S*,13*R*,15*S*,17*R*. Then, the structure of dictamlimonol C (**3**) was elucidated.

**Table 3:** ^1^H and ^13^C NMR data for **3** in CDCl_3_.

**No**.	**δ_**C**_**	**δ_**H**_ (*J* in Hz)**	**No**.	**δ_**C**_**	**δ_**H**_ (*J* in Hz)**
8	134.8		16	170.9	
9	77.1	3.46 (br. d, *ca*. 4)	17	79.5	5.47 (s)
11	21.6	1.73 (tt like, *ca*. 4, 15)	18	17.1	0.95 (s)
		1.98 (dt like, *ca*. 4, 15)	20	120.1	
12	27.3	1.09 (dt like, *ca*. 3, 15)	21	141.2	7.45 (t like, *ca*. 2)
		1.56 (dt like, *ca*. 4, 15)	22	110.0	6.40 (t like, *ca*. 2)
13	39.2		23	142.8	7.38 (t like, *ca*. 2)
14	136.7		30	17.0	1.90 (s)
15	66.9	4.90 (s)	9-OCH_3_	57.3	3.41 (s)

Dictamlimonol D (**4**) was a white powder with negative optical rotation [[α]_D_^25^ −68.0, MeOH]. Its molecular formula, C_17_H_22_O_5_, was determined by its quasi-molecular ion peak at *m/z* 351.14261 [M + COOH]^−^ (calcd for C_18_H_23_O_7_, 351.14383) in the negative ESI-Q-Orbitrap MS experiment, which was 14 amu greater than that of **3**. The ^1^H and ^13^C NMR (Table 4, CDCl_3_) signals of **4** were similar to those of **3** except for C-9. The 9-methoxy in the structure of **3** has changed into an ethoxy in **4** at δ_H_ 1.25 (3H, t, *J* = 7.0 Hz, H_3_-2'), 3.47, 3.70 (1H each, both m, H_2_-1'), which was consistent with the mass spectrometry data as well. In addition, in its HMBC spectra, the long-range correlation from H_2_-1' to C-9 was observed, which verified that the ethoxyl connected to the C-9 position. Meanwhile, the optical rotation, NOE correaltions (Figure 3), and ECD spectra (Figure 4D) of **4** and **3** were also basically consistent. Consequently, the structure of dictamlimonol D (**4**) was deduced.

**Table 4:** ^1^H and ^13^C NMR data for **4** in CDCl_3_.

**No**.	**δ_**C**_**	**δ_**H**_ (*J* in Hz)**	**No**.	**δ_**C**_**	**δ_**H**_ (*J* in Hz)**
8	134.9		17	79.4	5.48 (s)
9	75.3	3.56 (br. d, *ca*. 4)	18	17.2	0.95 (s)
11	22.6	1.75 (tt like, *ca*. 4, 15)	20	120.1	
		1.94 (m)	21	141.3	7.46 (t like, *ca*. 2)
12	27.4	1.08 (dt like, *ca*. 3, 13)	22	110.0	6.41 (t like, *ca*. 2)
		1.55 (dt like, *ca*. 3, 13)	23	142.8	7.37 (t like, *ca*. 2)
13	39.2		30	16.9	1.89 (s)
14	136.6		1′	65.2	3.47, 3.70 (both m)
15	66.9	4.91 (s)	2′	15.5	1.25 (t, 7.0)
16	170.9				

Dictamlimonol E (**5**) was a white powder, too. It also had negative optical rotation [[α]_D_^25^ −9.1, MeOH]. The ESI-Q-Orbitrap MS experiment results indicated that the molecular fomula of **5** was the same as that of **3**, C_16_H_20_O_5_ (*m/z* 337.12818 [M + COOH]^−^; calcd for C_17_H_21_O_7_, 337.12818). The same planar structure of **5** and **3** was revealed by the ^1^H, ^13^C NMR (Table 5, CDCl_3_), ^1^H ^1^H COZY, HSQC, and HMBC spectra. Meanwhile, the chemical shifts of C-9, 11, 12, 14, and 30 were different between these two compounds, which suggested they might be the C-9 epimers. In the NOESY spectrum of **5**, NOE correlations (Figure 3) were observed between the following proton and proton pairs: δ_H_ 1.14 (H_3_-18) and δ_H_ 1.76 (H-11α), 4.98 (H-15), 6.41 (H-22), δ_H_ 1.47 (H-12β) and δ_H_ 3.79 (H-9), 5.44 (H-17). As a result, the relative configuration of 9-OCH_3_ was deduced to be α. Finally, the absolute configuration of dictamlimonol E (**5**) was determined to be 9*R*,13*R*,15*S*,17*R* by ECD spectra (Figure 4E) comparison between experimental and calculated data.

**Table 5:** ^1^H and ^13^C NMR data for **5** in CDCl_3_.

**No**.	**δ_**C**_**	**δ_**H**_ (*J* in Hz)**	**No**.	**δ_**C**_**	**δ_**H**_ (*J* in Hz)**
8	137.7		16	170.8	
9	78.2	3.79 (dd, 8.0, 10.0)	17	79.9	5.44 (s)
11	23.3	1.76 (dddd, 4.0, 10.0, 13.5, 13.5)	18	18.4	1.14 (s)
		2.14 (m)	20	119.8	
12	31.2	1.32 (dt like, *ca*. 4, 14)	21	141.2	7.46 (t like, *ca*. 2)
		1.47 (dt like, *ca*. 4, 14)	22	109.9	6.41 (t like, *ca*. 2)
13	39.1		23	143.0	7.42 (t like, *ca*. 2)
14	134.9		30	14.6	1.86 (s)
15	67.3	4.98 (br. s)	9-OCH_3_	56.0	3.37 (s)

Dictamlimonol F (**6**) was a white powder with negative optical rotation [[α]_D_^25^ −18.5, MeOH]. Its molecular formula, C_15_H_18_O_5_, was deduced by the ion peak at *m/z* 323.11221 [M + COOH]^−^ (calcd for C_16_H_19_O_7_, 323.11253). The aglycone of **6** was suggested to be similar to those of **3**–**5** by the comparison of their ^1^H, ^13^C NMR spectra. The differences were as follows: the substituent of C-9 disappeared; Δ^8,14^ had changed into Δ^8,9^; and there was one more oxygenated quaternary carbon at δ_C_ 75.4 (C-14) in **6**. The presence of “–C=CH–CH_2_-CH_2_-” and “–CH=CH–O–” moieties was determined by the observation of its ^1^H ^1^H COZY spectrum. Moreover, the long-range correlations from H-9 to C-8, C-14; H-15 to C-13, C-14, C-16; H-17 to C-16, C-20–22; H_3_-18 to C-12–14, C-17; H_3_-30 to C-8, C-9, C-14 were observed in the HMBC spectrum. As a result, the planar structure of **6** was deduced. The NOE correlations (Figure 3) between H_3_-18 and H-12α, H-15, H-22; H-17 and H-12β were observed in its NOESY experiment. Moreover, Chem3D modeling was used to disclose the relative configuration of it (total energy of MM2 optimized calculation results: 33.9 kcal/mol for 14α-OH; 28.1 kcal/mol for 14β-OH). According to the relative stability, the relative configuration of **6** was speculated. The absolute configuration, 13*S*,14*S*,15*S*,17*S*, was clarified by comparing the experimental ECD spectrum (Figure 4F) with that of the calculated one.

Meanwhile, comparing the NMR data reported in the literatures, the structures of seven known compounds (**7**–**13**) were determined.

NO is closely associated with inflammation. Agents that block NO production might be beneficial for the treatment of inflammatory responses. The LPS-stimulated RAW 264.7 cells were used as a potential *in vitro* anti-inflammatory activity screening model to investigate the NO production inhibitory effect of limonoids **1**–**13** at a final concentration of 20 μM. Compared to the LPS group, compounds **5**, **7**–**11**, and **13** exhibited potential *in vitro* anti-inflammatory activities at 20 μM (Table 7).

**Table 6:** ^1^H and ^13^C NMR data for **6** in C_5_D_5_N.

**No**.	**δ_**C**_**	**δ_**H**_ (*J* in Hz)**	**No**.	**δ_**C**_**	**δ_**H**_ (*J* in Hz)**
8	135.3		16	172.7	
9	128.8	5.90 (m)	17	80.3	6.36 (s)
11	21.8	2.03 (m)	18	18.3	1.18 (s)
12	33.7	1.64 (ddd, 3.5, 3.5, 13.0)	20	122.8	
		2.35 (m)	21	141.1	7.76 (t like, *ca*. 2)
13	40.8		22	111.0	6.71 (t like, *ca*., 2)
14	75.4		23	143.4	7.65 (t like, *ca*., 2)
15	74.7	5.15 (s)	30	18.4	2.03 (s)

**Table 7:** Inhibitory effects of positive control and **1**–**13** on NO production in RAW 264.7 cells.

**No**.	**NRC (%)**	**No**.	**NRC (%)**	**No**.	**NRC (%)**
Normal	1.5 ± 0.5	**4**	102.2 ± 3.1	**10**	91.5 ± 2.6^**^
Control	100.0 ± 3.3	**5**	73.3 ± 2.6^***^	**11**	56.5 ± 3.5^***^
DEX	71.8 ± 0.9^***^	**6**	103.8 ± 3.0	**12**	97.4 ± 1.1
**1**	101.6 ± 3.7	**7**	90.4 ± 2.6^**^	**13**	10.5 ± 0.3^***^
**2**	99.2 ± 1.9	**8**	90.2 ± 0.7^**^		
**3**	99.9 ± 2.3	**9**	92.2 ± 2.4^**^		

*N, Unstimulated normal (negative control); C, Control group (stimulated by LPS); D, Dexamethasone (Dex, a positive control). Nitrite relative concentration (NRC): percentage of control group, which set as 100%. Values represent the mean ± SD of three determinations*.

** *P < 0.01*;

*** *P < 0.001 (differences between the compound-treated group and the control group). n = 4. Final concentration was 20 μM for **1**–**13**, and 1 μg/ml for Dex, respectively*.

**Figure 5:**
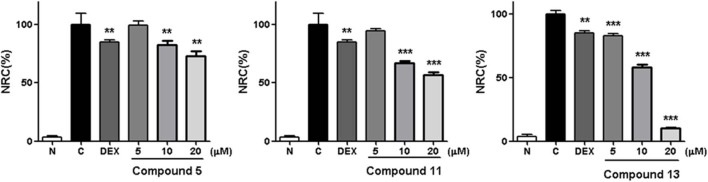
Inhibitory effects of **5**, **11**, and **13** in the concentration of 5, 10 and 20 μM on NO production in RAW 264.7 cells. N, normal group without LPS, DEX, and other tested samples. C, control group with LPS. Nitrite relative concentration (NRC): percentage of control group, set as 100%. Values represent the mean ± SD of three determinations. **P < 0.01; ***P < 0.001 (differences between the compound-treated group and the control group). *N* = 4. Final concentration was 5, 10, and 20 μM for **5**, **11**, and **13**, respectively.

Within the active compounds, **5**, **11**, and **13** were selected for further research. It was found they inhibited NO release from RAW 264.7 cells in a concentration-dependent manner at 5, 10, and 20 μM (Figure 5).

The structure–activity relationships on NO production in LPS-stimulated RAW 264.7 cells were summarized as follows: In compounds **2** and **11**–**13**, hydroxyl group or glucosyl group substitution at position 9 weakened the activity (**11** and **13** > **2** and **12**). Although the specimen was limited, carbonyl substitution at position 23 showed enhanced activity (**13** > **11**). In compounds **3**–**6**, α type alkoxy group substitution showed stronger inhibitory effects on NO production than β type (**5** > **3** and **4**).

LPS can stimulate the acute inflammatory response by increasing expression of tumor necrosis factor (TNF-α) and interleukin 6 (IL-6), inducible nitric oxide synthase (iNOS), nuclear factor kappa-light-chain-enhancer of activated B cells (NF-κB), and cyclooxygenase-2 (COX-2), successively (Ahujaa et al., 2019). In the process, the expressions of TNF-α, IL-6, iNOS/NO, NF-κB, as well as COX-2 will be upregulated, which will promote tissue damage and chronic disease. Therefore, the Western blot method was used to study the anti-inflammatory mechanism of compounds **5**, **11**, and **13** by determining the levels of these five proteins in LPS-stimulated RAW 264.7 cells.

**Figure 6:**
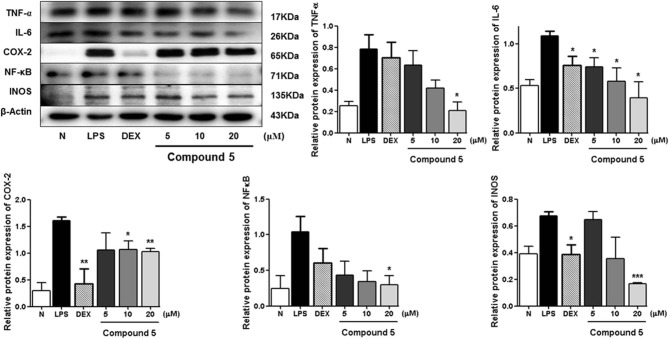
Inhibitory effects of **5** on the protein expression of TNF-α, IL-6, COX-2, NF-κB, and iNOS in RAW 264.7 cells. N, normal group without LPS, DEX, and other tested samples. Values represent the mean ± SEM of three determinations. *P < 0.05; **P < 0.01; ***P < 0.001 (differences between the compound-treated group and the control group) *N* = 3.

**Figure 7:**
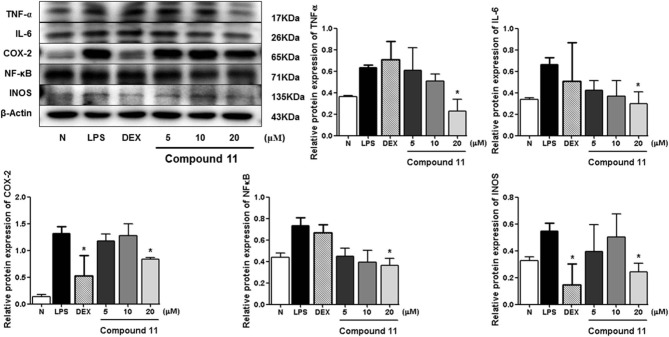
Inhibitory effects of compound **11** on the protein expression of TNF-α, IL-6, COX-2, NF-κB, and iNOS in RAW 264.7 cells. N, normal group without LPS, DEX, and other tested samples. Values represent the mean ± SEM of three determinations. *P < 0.05 (differences between the compound-treated group and the control group) *N* = 3.

**Figure 8:**
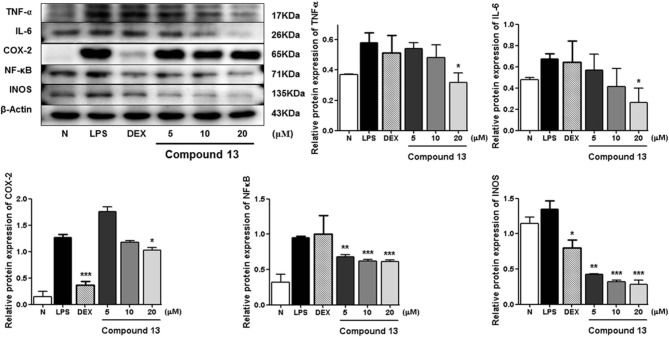
Inhibitory effects of compound **13** on the protein expression of TNF-α, IL-6, COX-2, NF-κB, and iNOS in RAW 264.7 cells. N, normal group without LPS, DEX, and other tested samples. Values represent the mean ± SEM of three determinations. *P < 0.05; **P < 0.01; ***P < 0.001 (differences between the compound-treated group and the control group) *N* = 3.

Compared with the normal group, LPS treatment led to an obvious upregulation in the protein expressions of TNF-α, IL-6, NF-κB, iNOS, and COX-2. Compounds **5**, **11**, and **13** were found to inhibit the expression of the abovementioned proteins in a dose-dependent manner (Figures 6–8).

Among the anti-inflammatory active compounds, fraxinellone (**11**) had been found to inhibit LPS-induced NO production and reduce the LPS-induced expressions of iNOS and COX-2 at the mRNA and protein levels in a dose-dependent manner by regulating NF-κB in RAW 264.7 macrophage-like cells (Kim et al., 2009; Lee et al., 2009; Wu et al., 2014), which was identical to our experimental result, suggesting that our screening system was stable and suitable. Meanwhile, the effects and the mechanism of a new compound, dictamlimonol D (**5**), as well as the known compound, dasylactone A (**13**), in inflammation were characterized here firstly, which would provide new candidate drugs for inflammation-related diseases.

Since fraxinellone (**11**) is the main constituent in *Cortex Dictamni*, and the content of it is about 0.15% in *Cortex Dictamni*, we can predict that the anti-inflammatory activity of the plant is mainly derived from limonoids, especially fraxinellone. Further mechanism and clinical studies for it is necessary.

## Materials and Methods

### Materials and Methods for Phytochemistry Research

#### General Experimental Procedures

Optical rotations were measured on a Rudolph Autopol® IV automatic polarimeter (l = 50 mm) (Rudolph Research Analytical, Hackettstown NJ, USA). NMR spectra were determined on a Bruker 500-MHz NMR spectrometer (Bruker BioSpin AG Industriestrasse 26 CH-8117, Fällanden, Switzerland) at 500 MHz for ^1^H and 125 MHz for ^13^C NMR (internal standard: TMS). IR spectra were recorded on a Varian 640-IR FT-IR spectrophotometer (Varian Australia Pty Ltd., Mulgrave, Australia). Negative-ion mode ESI-Q-Orbitrap-MS was obtained on a Thermo ESI-Q-Orbitrap MS mass spectrometer connected with the UltiMate 3000 UHPLC instrument via ESI interface (Thermo Fisher Scientific, Inc., Waltham, MA, USA).

CC was performed on macroporous resin D101 (Haiguang Chemical Co., Ltd., Tianjin, China), silica gel (48–75 μm, Qingdao Haiyang Chemical Co., Ltd., Qingdao, China), and ODS (40–63 μm, YMC Co., Ltd., Tokyo, Japan). pHPLC columns, Cosmosil 5C_18_-MS-II (20 mm i.d. ×250 mm, Nacalai Tesque, Inc., Kyoto, Japan), and Cosmosil PBr (20 mm i.d. ×250 mm, Nacalai Tesque, Inc., Kyoto, Japan) were used to separate the constituents.

#### Plant Material

*Cortex Dictamni* was purchased from the medicine market in Anguo city, Heibei province, China, and identified by Dr. Li Tianxiang (Experiment Teaching Department, Tianjin University of Traditional Chinese Medicine). The voucher specimen was deposited at the Academy of Traditional Chinese Medicine of Tianjin University of TCM.

#### Extraction and Isolation

*Cortex Dictamni* (9.0 kg) was refluxed with 70% EtOH-H_2_O. The 70% EtOH extract (1725.3 g) was partitioned in an EtOAc-H_2_O mixture (1:1, v/v). The H_2_O layer (1253.8 g) was subjected to D101 macroporous resin CC (H_2_O → 95% EtOH). Then, H_2_O (1032.9 g) and 95% EtOH (123.4 g) eluates were obtained.

The 95% EtOH eluate (90.0 g) was subjected to silica gel CC [CHCl_3_ → CHCl_3_-MeOH (100:1 → 100:3 → 100:7, v/v) → CHCl_3_-MeOH-H_2_O (10:3:1 → 7:3:1 → 6:4:1, v/v/v, lower layer) → MeOH], and 12 fractions (Fr. 1–Fr. 12) were afforded. Fraction 7 (4.0 g) was isolated by ODS CC [MeOH-H_2_O (10% → 20% → 30% → 40% → 50% → 60% → 100%, v/v)] to give nine fractions (Fr. 7-1–Fr. 7-9). Fraction 7-6 (923.7 mg) was separated by Sephadex LH-20 CC (MeOH), and three fractions (Fr. 7-6-1–Fr. 7-6-3) were obtained. Fraction 7-6-2 (526.5 mg) was purified by pHPLC [MeOH-H_2_O (50:50, v/v), Cosmosil 5C_18_-MS-II column] to gain dictamlimonoside B (**2**, 27.4 mg).

The EtOAc layer (175.0 g, E) was isolated by silica gel CC [hexane-EtOAc (100:1 → 20:1 → 10:1 → 5:1 → 3:1 → 1:1 → 0:1, v/v) → MeOH] to afford 10 fractions (Fr. E-1–Fr. E-10). Fraction E-4 (25.2 g) was separated by silica gel CC [hexane-EtOAc (1:1, v/v)] to gain fraxinellone (**11**, 8.0 g). Fraction E-7 (3.2 g) was isolated by pHPLC [MeOH-H_2_O (80:20, v/v), Cosmosil 5C_18_-MS-II column], and nine fractions (Fr. E-7-1–Fr. E-7-9) were obtained. Fraction E-7-2 (278.5 mg) was purified by pHPLC [MeOH-H_2_O (75:25, v/v), Cosmosil PBr column] to afford dictamlimonol D (**4**, 56.9 mg). Fraction E-8 (37.0 g) was centrifuged after dissolving in hexane, and two fractions (Fr. E-8-1–Fr. E-8-2) were gained. Fraction E-8-1 (70.0 mg) was isolated by pHPLC [MeOH-H_2_O (50:50, v/v), Cosmosil 5C_18_-MS-II column] to obtain dictamlimonol A (**1**, 8.7 mg) and limonin (**7**, 22.3 mg). Fraction E-8-2 (35.16 g) was subjected to silica gel CC [PE-EtOAc (5:1 → 3:1 → 2:1 → 1:1 → 0:1, v/v)], and nine fractions (Fr. E-8-2-1–Fr. 8-2-9) were afforded. Fraction E-8-2-5 (4.4 g) was separated by pHPLC [MeOH-H_2_O (67:33, v/v), Cosmosil 5C_18_-MS-II column] to provide nine fractions (Fr. E-8-2-5-1–Fr. E-8-2-5-9). Fraction E-8-2-5-1 (1396.6 mg) was subjected to pHPLC [MeOH-H_2_O (65:35, v/v), Cosmosil PBr column], and eight fractions (Fr. E-8-2-5-1-1–Fr. E-8-2-5-1-8) were given. Fraction E-8-2-5-1-3 (190.9 mg) was purified by pHPLC [MeOH-H_2_O (45:55, v/v), Cosmosil 5C_18_-MS-II column] to afford dictamlimonol F (**6**, 22.1 mg) and 9β-hydroxyfraxinellone (**12**, 33.8 mg). Fraction E-8-2-5-1-4 (81.0 mg) was isolated by pHPLC [MeOH-H_2_O (55:45, v/v), Cosmosil PBr column] to gain dictamlimonol E (**5**, 12.2 mg). Fraction E-8-2-5-1-6 (81.2 mg) was further subjected to pHPLC [MeOH-H_2_O (65:35, v/v), Cosmosil PBr column], and dictamlimonol C (**3**, 63.1 mg) was afforded. Fraction E-8-2-6 (7.1 g) was separated by silica gel CC [PE-EtOAc (5:1 → 4:1 → 3:1 → 2:1 → 1:1 → 0:1, v/v) → MeOH] to afford eight fractions (Fr. E-8-2-6-1–Fr. E-8-2-6-8). Among them, fraction E-8-2-6-8 was identified as obacunon (**9**, 1380.0 mg). Fraction E-8-2-6-4 (3483.7 mg) was isolated by pHPLC [MeOH-H_2_O (85:15, v/v), Cosmosil 5C_18_-MS-II column], and 10 fractions (Fr. E-8-2-6-4-1–Fr. E-8-2-6-4-10) were obtained. Fraction E-8-2-6-4-1 (778.2 mg) was subjected to pHPLC [CH_3_CN-H_2_O (42:58, v/v), Cosmosil 5C_18_-MS-II column] to provide six fractions (Fr. E-8-2-6-4-1-1–Fr. E-8-2-6-4-1-6). Fraction E-8-2-6-4-1-6 (70.5 mg) was purified by pHPLC [MeOH-H_2_O (60:40, v/v), Cosmosil PBr column] to gain dasylactone A (**13**, 32.4 mg). Fraction E-8-2-6-4-2 (693.5 mg) was isolated by pHPLC [CH_3_CN-H_2_O (42:58, v/v), Cosmosil 5C_18_-MS-II column] to afford five fractions (Fr. E-8-2-6-4-2-1–Fr. E-8-2-6-4-2-5). Fraction E-8-2-6-4-2-5 (50.8 mg) was further purified by pHPLC [MeOH-H_2_O (70:30, v/v), Cosmosil 5C_18_-MS-II column], and 7α-obacunyl acetate (**10**, 9.6 mg) was obtained. Fraction E-8-2-7 (3.1 g) was separated by pHPLC [MeOH-H_2_O (62:38, v/v), Cosmosil 5C_18_-MS-II column] to obtain seven fractions (Fr. E-8-2-7-1–Fr. E-8-2-7-7). Fraction E-8-2-7-3 (80.6 mg) was purified by pHPLC [MeOH-H_2_O (57:43, v/v), Cosmosil 5C_18_-MS-II column] to gain limonin diosphenol (**8**, 16.7 mg).

Dictamlimonol A (**1**). White powder; [α]_D_^25^ −100.0 (*conc* 0.10, MeOH); UV λ_max_ (MeOH) nm (log ε): 204 (4.05), 280 (3.35); CD (*conc*. 0.002 M, CH_3_CN) mdeg (λ_nm_): −18.3 (223), −14.8 (223), −15.3 (228), +2.7 (271), −4.1 (307); IR ν_max_ (KBr) cm^−1^: 3,515, 3,243, 2,985, 2,951, 1,746, 1,708, 1,652, 1,458, 1,390, 1,368, 1,281, 1,220, 1,176, 1,131, 1,105, 1,058, 1,023, 960, 898, 884, 828, 796; ^1^H NMR (C_5_D_5_N, 500 MHz), ^13^C NMR (C_5_D_5_N, 125 MHz); see Table 1. ESI-Q-Orbitrap MS Negative-ion mode *m/z* 485.17969 [M – H]^−^ (calcd for C_26_H_29_O_9_, 485.18061) (Figures S1–S8).

Dictamlimonoside B (**2**). White powder; [α]_D_^25^ −72.1 (*conc* 0.61, MeOH); UV λ_max_ (MeOH) nm (log ε): 213 (4.20); CD (*conc*. 0.002 M, CH_3_CN) mdeg (λ_nm_): −17.5 (204), −12.4 (216), −21.8 (231), +13.0 (259); IR ν_max_ (KBr) cm^−1^: 3,359, 2,931, 2,876, 1,755, 1,678, 1,504, 1,448, 1,406, 1,378, 1,344, 1,289, 1,226, 1,209, 1,161, 1,077, 1,048; ^1^H NMR (CD_3_OD/DMSO-*d*_6_, 500 MHz), ^13^C NMR (CD_3_OD/DMSO-*d*_6_, 125 MHz); see Table 2. ESI-Q-Orbitrap MS Negative-ion mode *m/z* 455.15375 [M + COOH]^−^ (calcd for C_21_H_27_O_11_, 455.15479) (Figures S9–S19).

Dictamlimonol C (**3**). White powder; [α]_D_^25^ −74.6 (*conc* 0.81, MeOH); UV λ_max_ (MeOH) nm (log ε): 206 (4.10); CD (*conc*. 0.002 M, CH_3_CN) mdeg (λ_nm_): −15.4 (200), +4.9 (222), −4.7 (240), +1.8 (268), −0.2 (355); IR ν_max_ (KBr) cm^−1^: 3,445, 3,152, 2,973, 2,941, 2,863, 2,825, 1,748, 1,653, 1,505, 1,458, 1,381, 1,350, 1,327, 1,278, 1,192, 1,162, 1,133, 1,109, 1,070, 1,025, 994, 907, 874, 800, 764, 732; ^1^H NMR (CDCl_3_, 500 MHz), ^13^C NMR (CDCl_3_, 125 MHz); see Table 3. ESI-Q-Orbitrap MS Negative-ion mode *m/z* 337.12790 [M + COOH]^−^ (calcd for C_17_H_21_O_7_, 337.12818) (Figures S20–S27).

Dictamlimonol D (**4**). White powder; [α]_D_^25^ −68.0 (*conc* 0.88, MeOH); UV λ_max_ (MeOH) nm (log ε): 206 (4.10), 239 (3.51, sh); CD (*conc*. 0.002 M, CH_3_CN) mdeg (λ_nm_): −11.3 (201), +0.8 (223), −1.1 (240), +0.8 (262), −0.2 (350); IR ν_max_ (KBr) cm^−1^: 3,383, 3,147, 2,973, 2,941, 2,869, 1,750, 1,653, 1,506, 1,457, 1,380, 1,345, 1,325, 1,278, 1,265, 1,198, 1,162, 1,064, 1,024, 1,001, 911, 875, 800, 765, 731; ^1^H NMR (CDCl_3_, 500 MHz), ^13^C NMR (CDCl_3_, 125 MHz); see Table 4. ESI-Q-Orbitrap MS Negative-ion mode *m/z* 351.14261 [M + COOH]^−^ (calcd for C_18_H_23_O_7_, 351.14383) (Figures S28–S35).

Dictamlimonol E (**5**). White powder; [α]_D_^25^ −9.1 (*conc* 0.35, MeOH); UV λ_max_ (MeOH) nm (log ε): 208 (3.94), 253 (3.04, sh); CD (*conc*. 0.001 M, CH_3_CN) mdeg (λ_nm_): −17.3 (195), +11.3 (224), +4.1 (251), −1.1 (347); IR ν_max_ (KBr) cm^−1^: 3,481, 3,148, 2,943, 2,876, 2,823, 1,749, 1,653, 1,506, 1,456, 1,380, 1,353, 1,282, 1,222, 1,163, 1,093, 1,023, 996, 911, 877, 802, 765, 731; ^1^H NMR (CDCl_3_, 500 MHz), ^13^C NMR (CDCl_3_, 125 MHz); see Table 5. ESI-Q-Orbitrap MS Negative-ion mode *m/z* 337.12818 [M + COOH]^−^ (calcd for C_17_H_21_O_7_, 337.12818) (Figures S36–S43).

Dictamlimonol F (**6**). White powder; [α]_D_^25^ −18.5 (*conc* 0.52, MeOH); UV λ_max_ (MeOH) nm (log ε): 203 (4.01), 249 (3.06, sh); CD (*conc*. 0.002 M, CH_3_CN) mdeg (λ_nm_): +23.9 (207), −21.4 (234), +1.7 (266); IR ν_max_ (KBr) cm^−1^: 3,447, 3,149, 2,972, 2,944, 2,889, 1,745, 1,668, 1,550, 1,504, 1,450, 1,384, 1,305, 1,278, 1,160, 1,143, 1,023, 992, 970, 875, 806, 771, 758, 719; ^1^H NMR (C_5_D_5_N, 500 MHz), ^13^C NMR (C_5_D_5_N, 125 MHz); see Table 6. ESI-Q-Orbitrap MS Negative-ion mode *m/z* 323.11221 [M + COOH]^−^ (calcd for C_16_H_19_O_7_, 323.11253) (Figures S44–S51).

#### Enzymatic Hydrolysis of 2 With β-Glucosidase

Compound **2** (10.0 mg) was hydrolyzed with β-glucosidase (10.0 mg) in H_2_O (1.0 ml) at 37°C for 5 h. After cooling, the reaction mixture was partitioned with EtOAc. The EtOAc layer was separated by silica gel CC [Hexane-EtOAc (3:2, v/v)] to yield 9β-hydroxyfraxinellone (**12**, 5.1 mg, 84.3%).

#### Acid Hydrolysis of 2

Acid hydrolysis reaction was performed by using a similar method to our previously reported one (Zhang et al., 2015), and d-glucose (12.6 min, positive optical rotation) from **2** was identified by comparison of its retention time and optical rotation with that of the authentic sample.

#### ECD Calculation

The calculations for ECD spectra were conducted as published previously (Shi et al., 2019).

### Materials and Methods for Anti-inflammatory Assay

#### Materials

The materials for anti-inflammatory assay were similar to those reported by us (Ruan et al., 2019).

#### Cell Culture

RAW 264.7 macrophage-like cells were cultured by the method reported previously (Ruan et al., 2019).

#### Cell Viability Assay

Cell viability of RAW 264.7 macrophage-like cells was performed by MTT colorimetric assay (result shown in Figure S52) (Ruan et al., 2019).

#### Measurement of NO Levels

After pretreating compounds **1–13** to cells for 1 h, the cells were stimulated with LPS (1 μg/ml) for 18 h. Each culture medium (50 μl) was mixed with an equal volume of Griess reagent after incubation. An ELISA plate reader was used to determine the nitrite level (a major stable product of NO) at 540 nm, and the concentrations were calculated by referring to a NaNO_2_ standard calibration curve.

#### Western Blot Analysis

Western blot method was used to study the anti-inflammatory mechanism of compounds **5**, **11**, and **13** by determining the levels of these five proteins (TNF-α, IL-6, COX-2, NF-κB, and iNOS) in LPS-stimulated RAW 264.7 cells as previously reported (He et al., 2018; Ruan et al., 2019). The raw quantification data were displayed in Figures S53–S55.

#### Statistical Analysis

Values were statistically analyzed by using SPSS 17.0 software. *P* < 0.05 was considered to indicate statistical significance. One-way analysis of variance (ANOVA) and Tukey's Studentized range test were used for the evaluation of the significant differences between means and *post hoc*, respectively.

## Conclusion

In this study, 13 limonoids including 6 new ones, named dictamlimonol A (**1**), dictamlimonoside B (**2**), and dictamlimonols C–F (**3**–**6**), along with 7 known ones (**7**–**13**) were isolated from *Cortex Dictamni* by various chromatographies and identified by spectroscopies and chemical reactions. Among them, compound **1** was a first 7,19-epoxy limonoid found in natural products. Activity evaluation research showed several kinds of limonoids reduce expression of TNF-α, IL-6, iNOS, NF-κB, and COX-2 in LPS-stimulated RAW 264.7 cells. These findings support the idea that the administration of *Cortex Dictamni* may be beneficial for inflammation.

## Data Availability Statement

The datasets generated for this study are available on request to the corresponding author.

## Author Contributions

YiZ and TW designed the research and wrote the manuscript. JR and YC performed the activity research. FS, SY, YinZ, and JY contributed to the isolation, purification, and characterization of all compounds. HW and YG performed the ECD calculation. HY perfected the language. All authors discussed, edited, and approved the final version.

### Conflict of Interest

The authors declare that the research was conducted in the absence of any commercial or financial relationships that could be construed as a potential conflict of interest.
