# Supplementary material for: Anti-inflammatory Limonoids From Cortex Dictamni
Source: Front Chem. 2020 Feb 28;8:73. doi: 10.3389/fchem.2020.00073 (PMC7058982; doi:10.3389/fchem.2020.00073)
Supplement: Supplementary file 1 [file Data_Sheet_1.pdf]

Supplementary Material

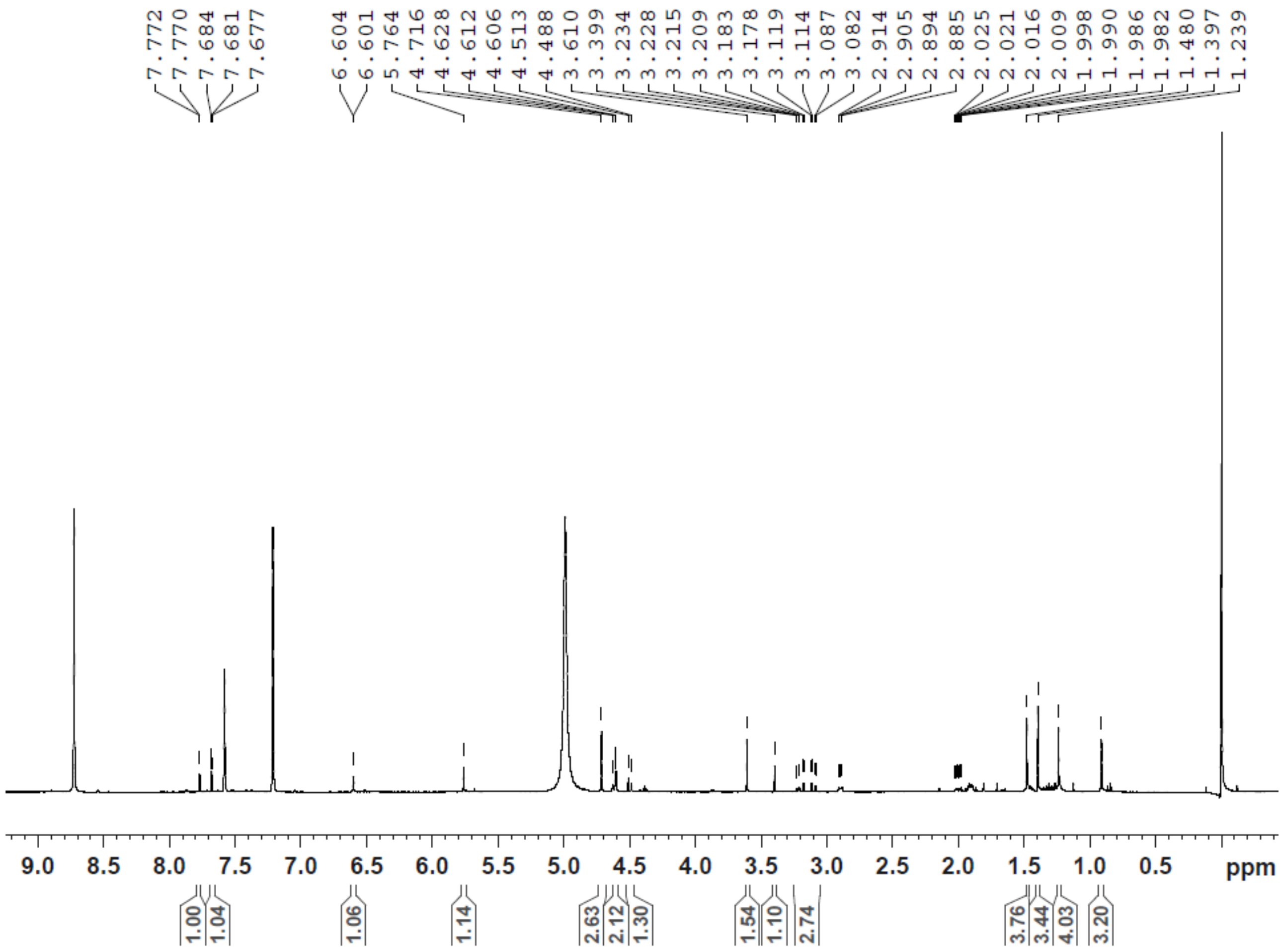

Figure S1 ^{1}H NMR (500 MHz, C_5D_5N) spectrum of compound **1**

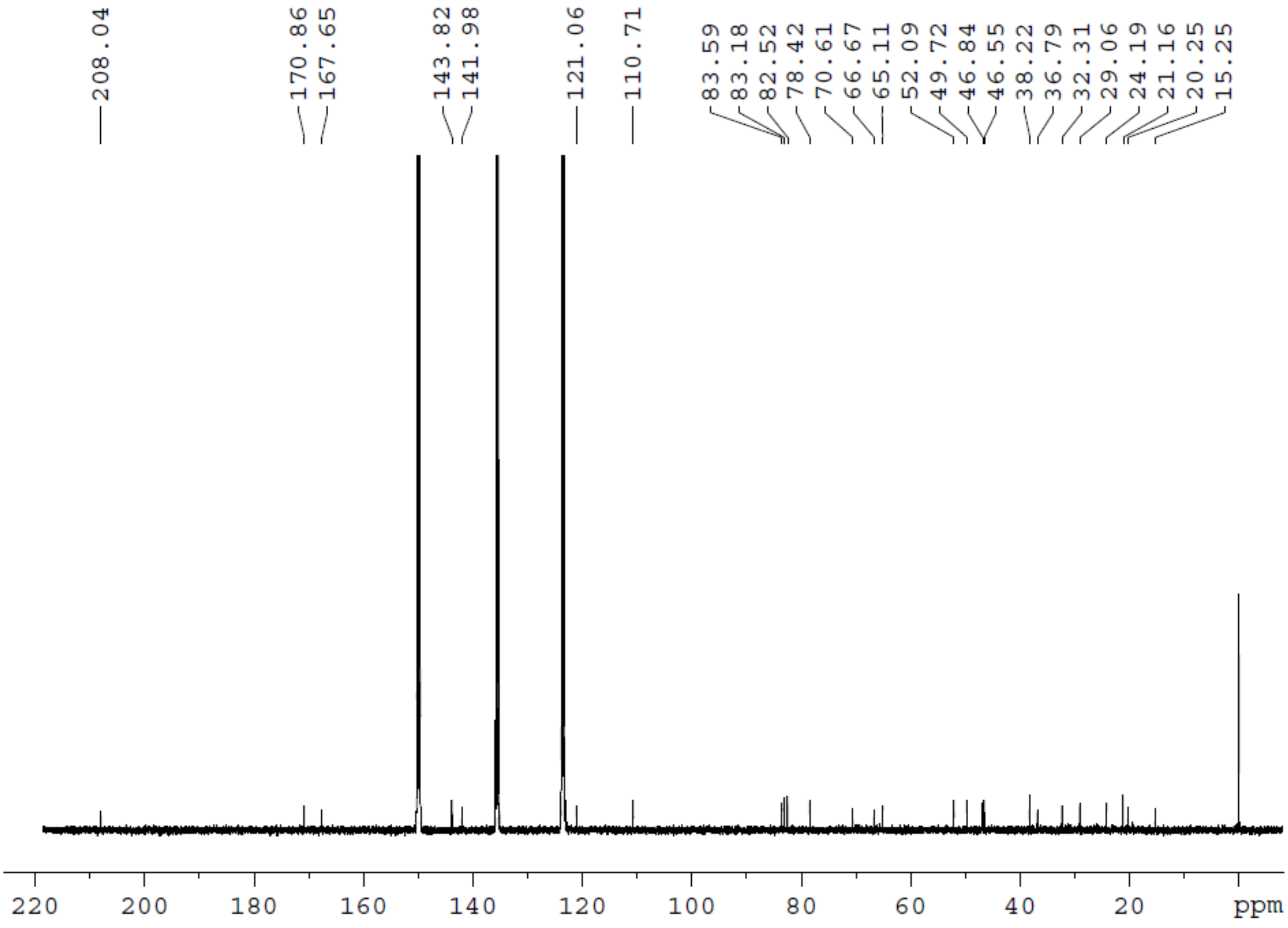

Figure S2 ^{13}C NMR (125 MHz, C_5D_5N) spectrum of compound **1**

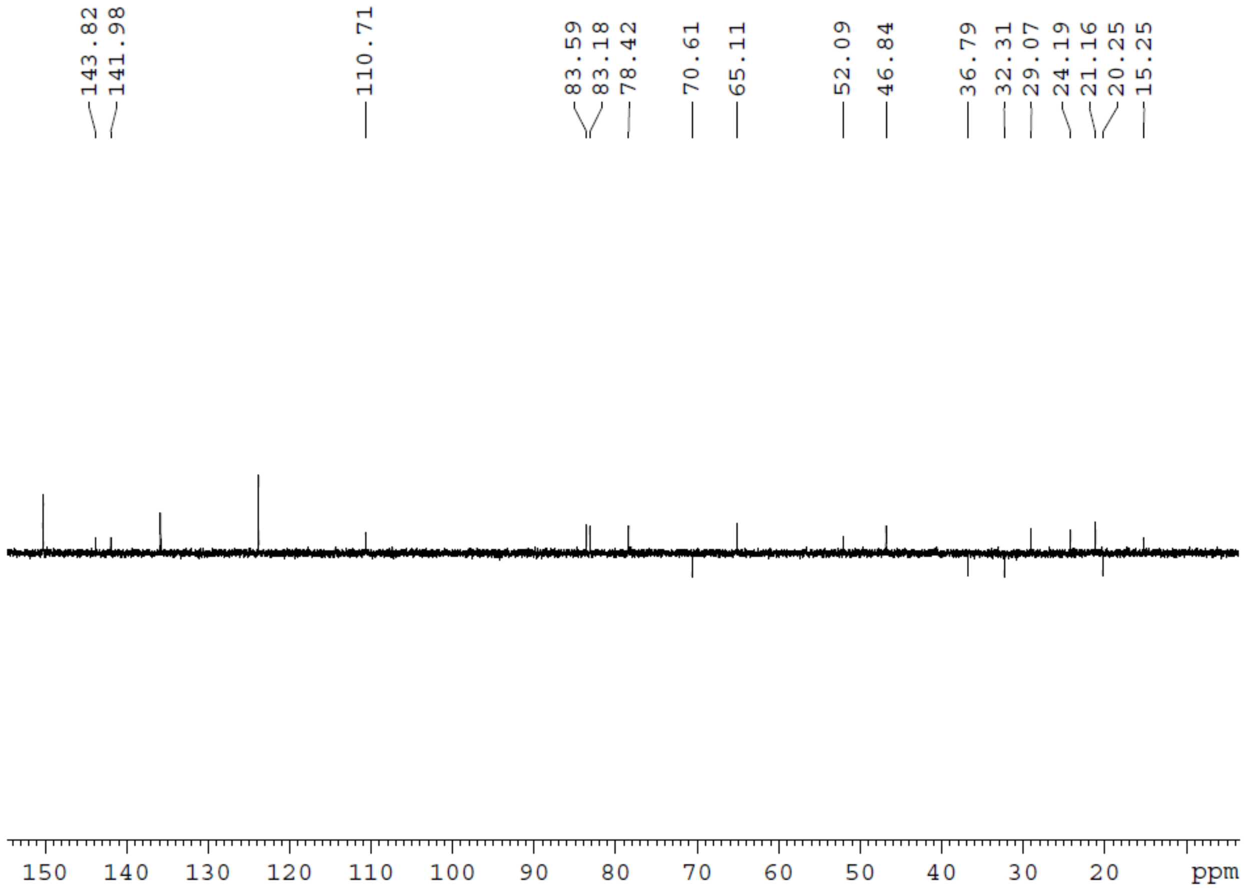

Figure S3 DEPT 135 (C_5D_5N) spectrum of compound **1**

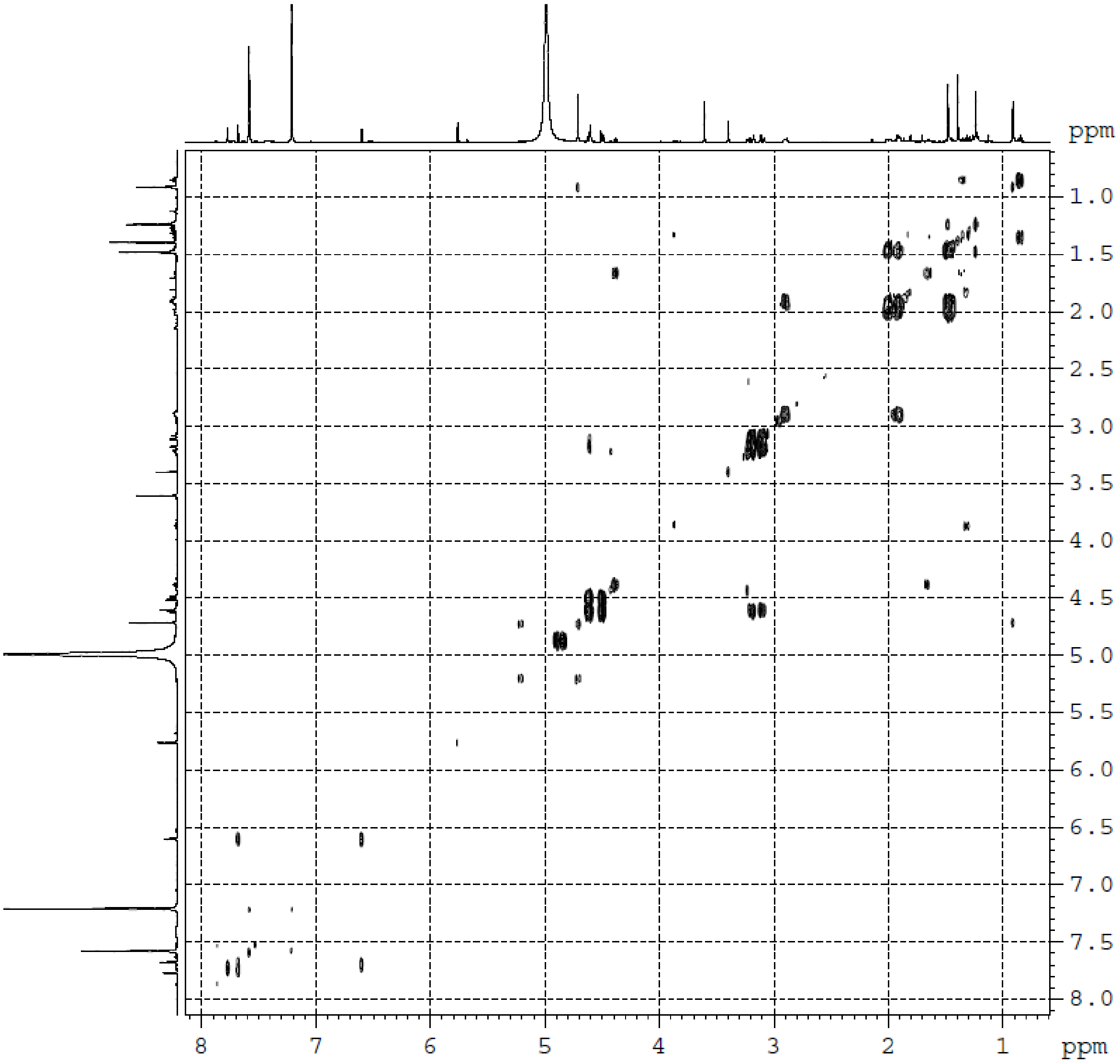

Figure S4 ^{1}H ^{1}H COSY (C_5D_5N) spectrum of compound **1**

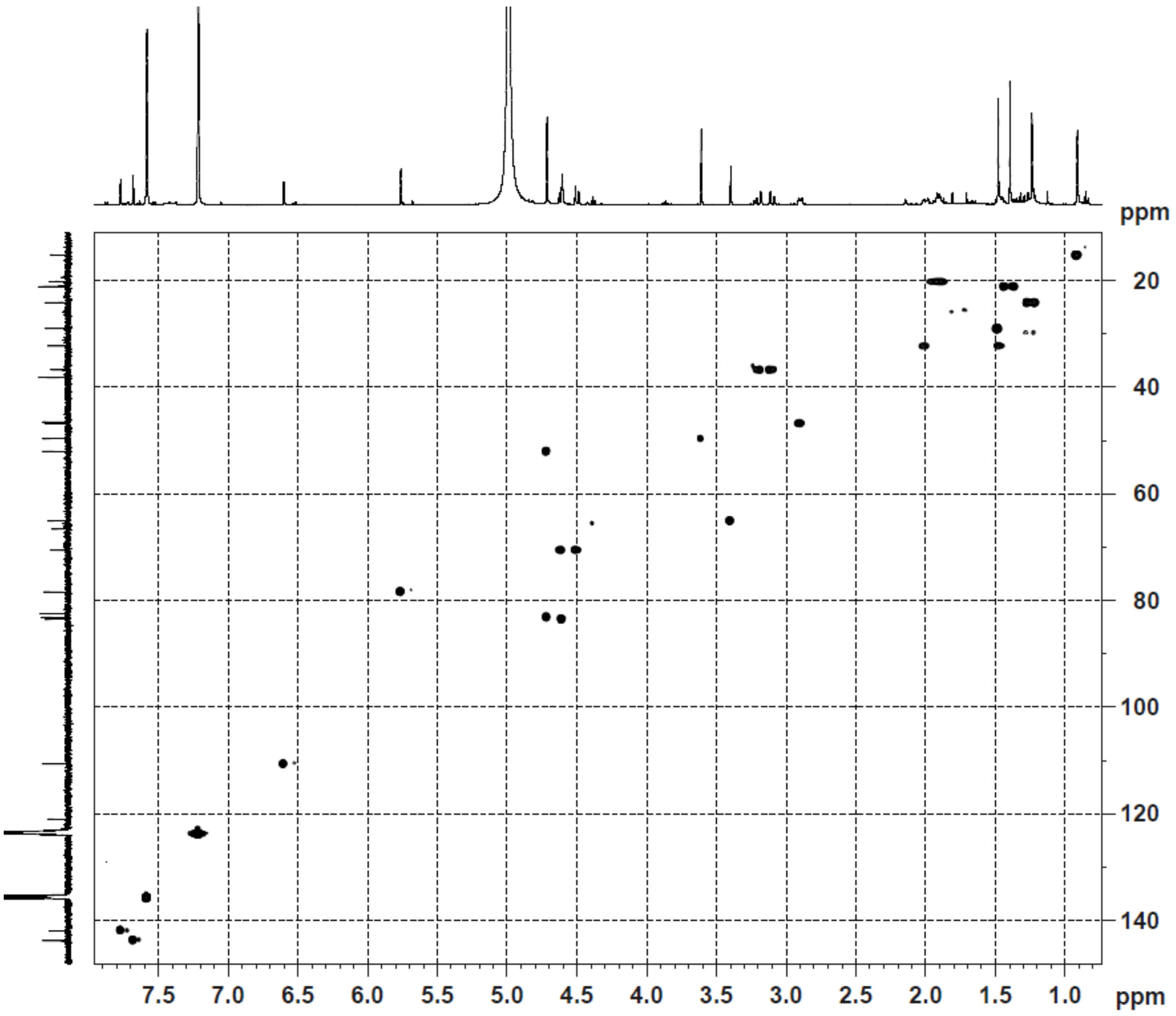

Figure S5 HSQC (C_5D_5N) spectrum of compound **1**

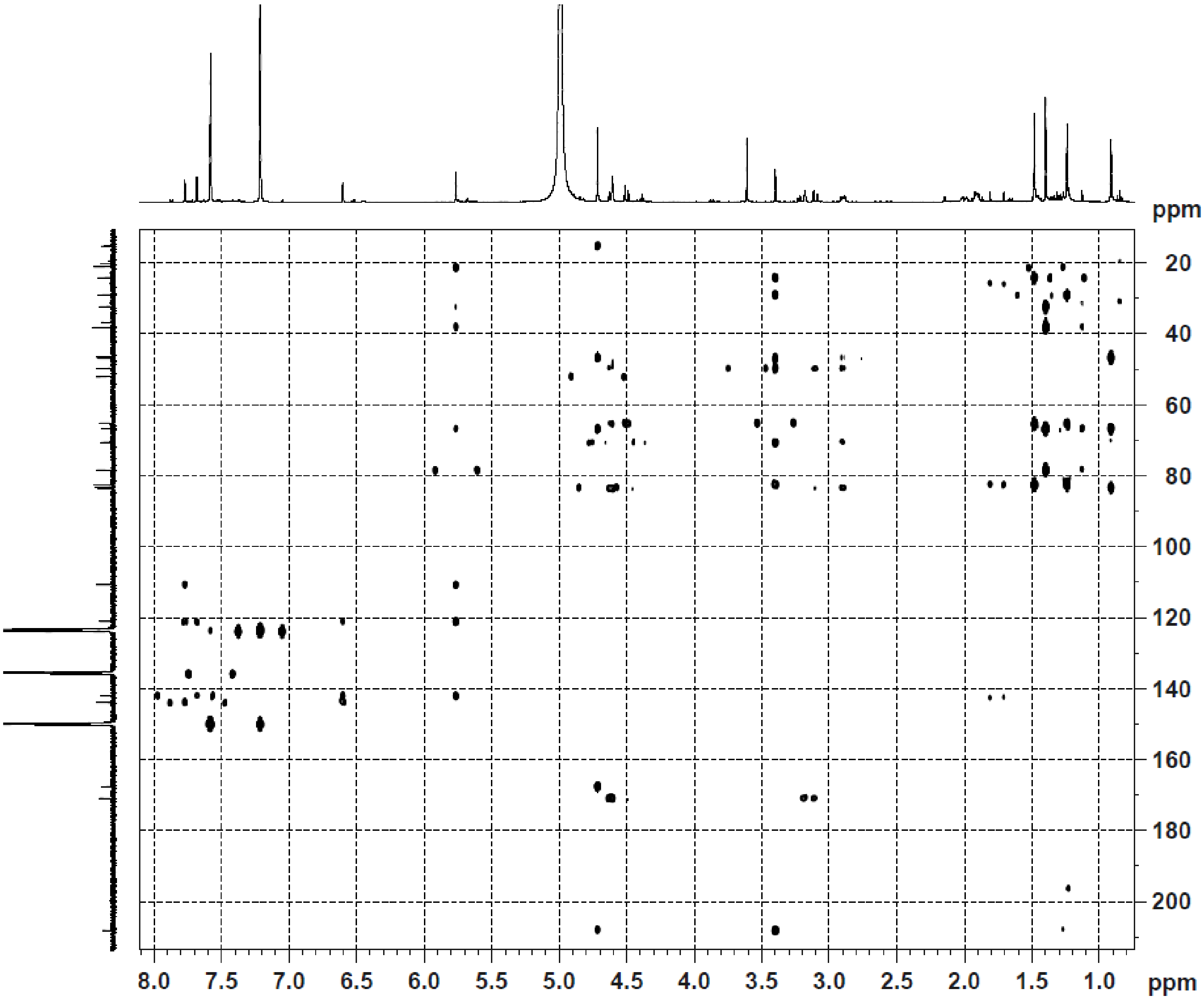

Figure S6 HMBC (C_5D_5N) spectrum of compound **1**

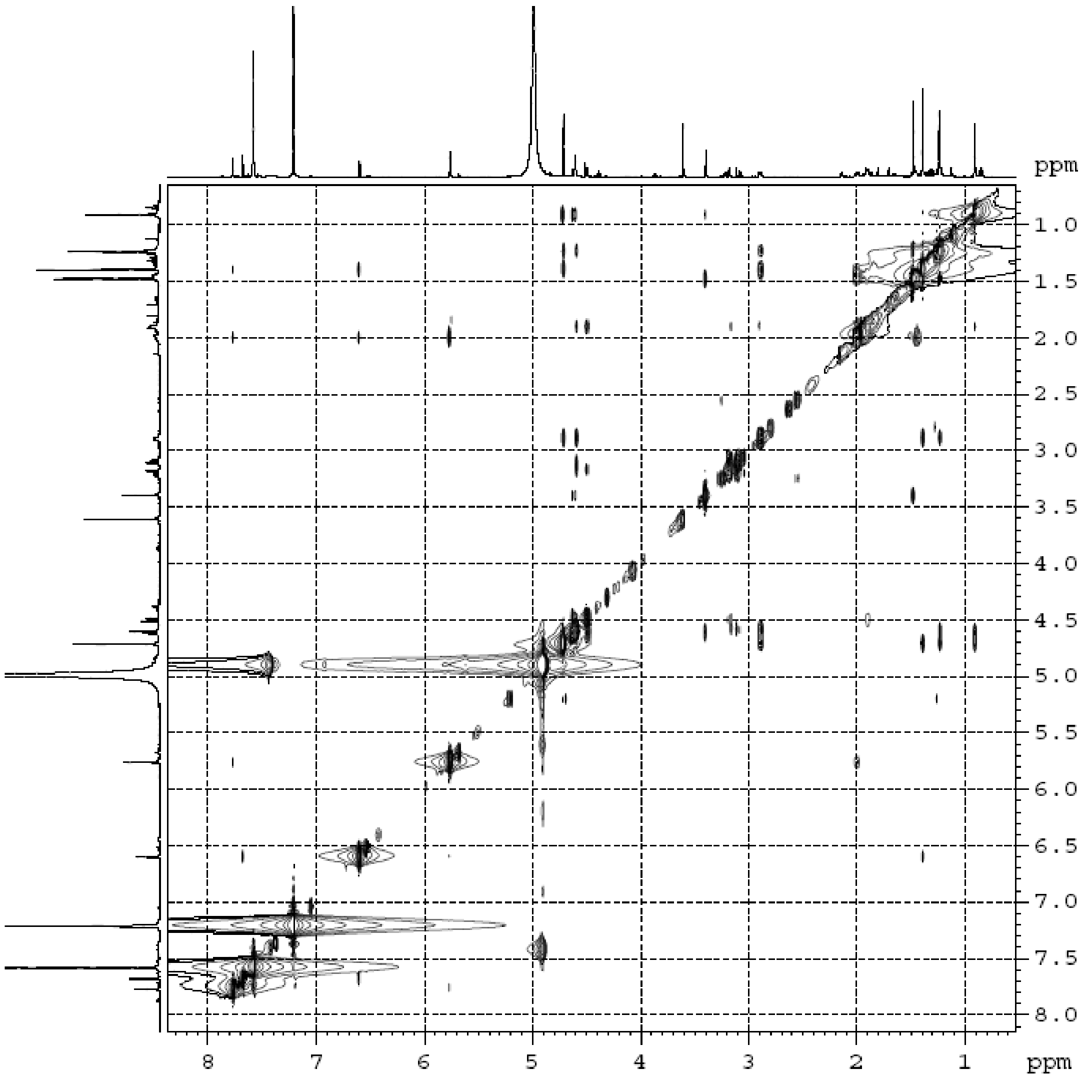

Figure S7 NOSEY (C_5D_5N) spectrum of compound **1**

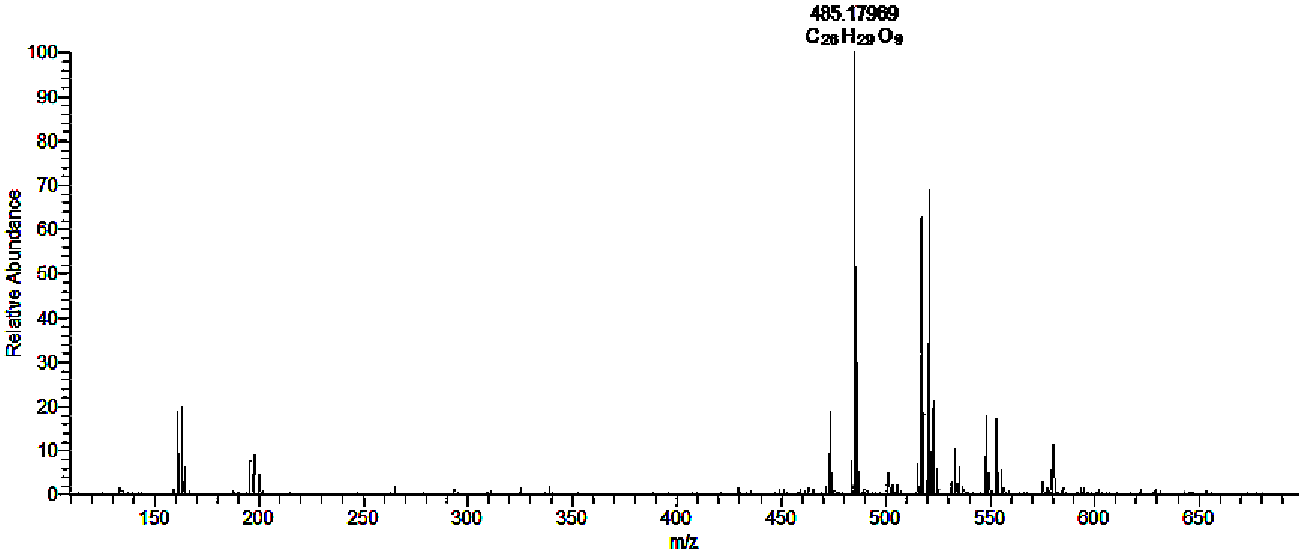

Figure S8 HRESI-Orbitrap-MS spectrum of compound **1**

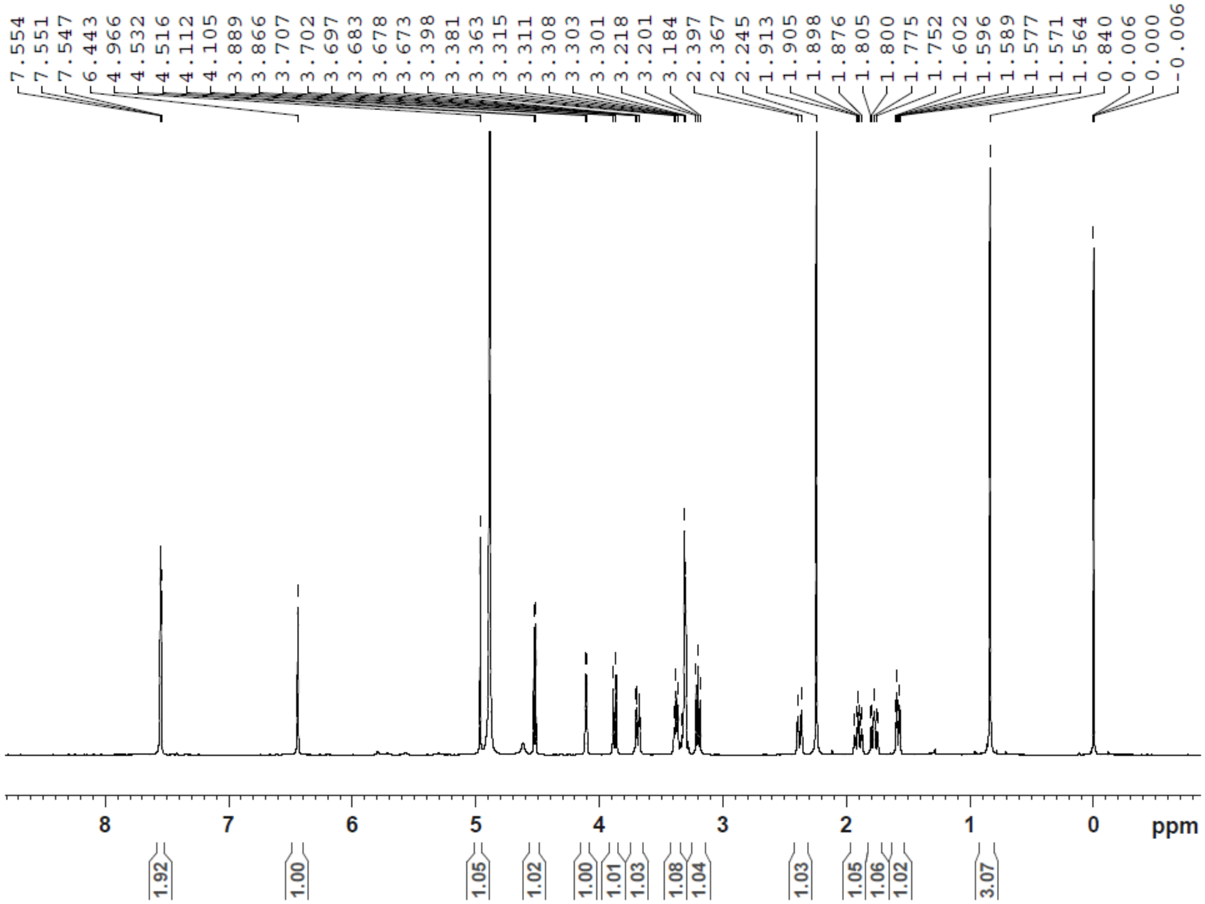

Figure S9 ^{1}H NMR (500 MHz, CD_3OD) spectrum of compound **2**

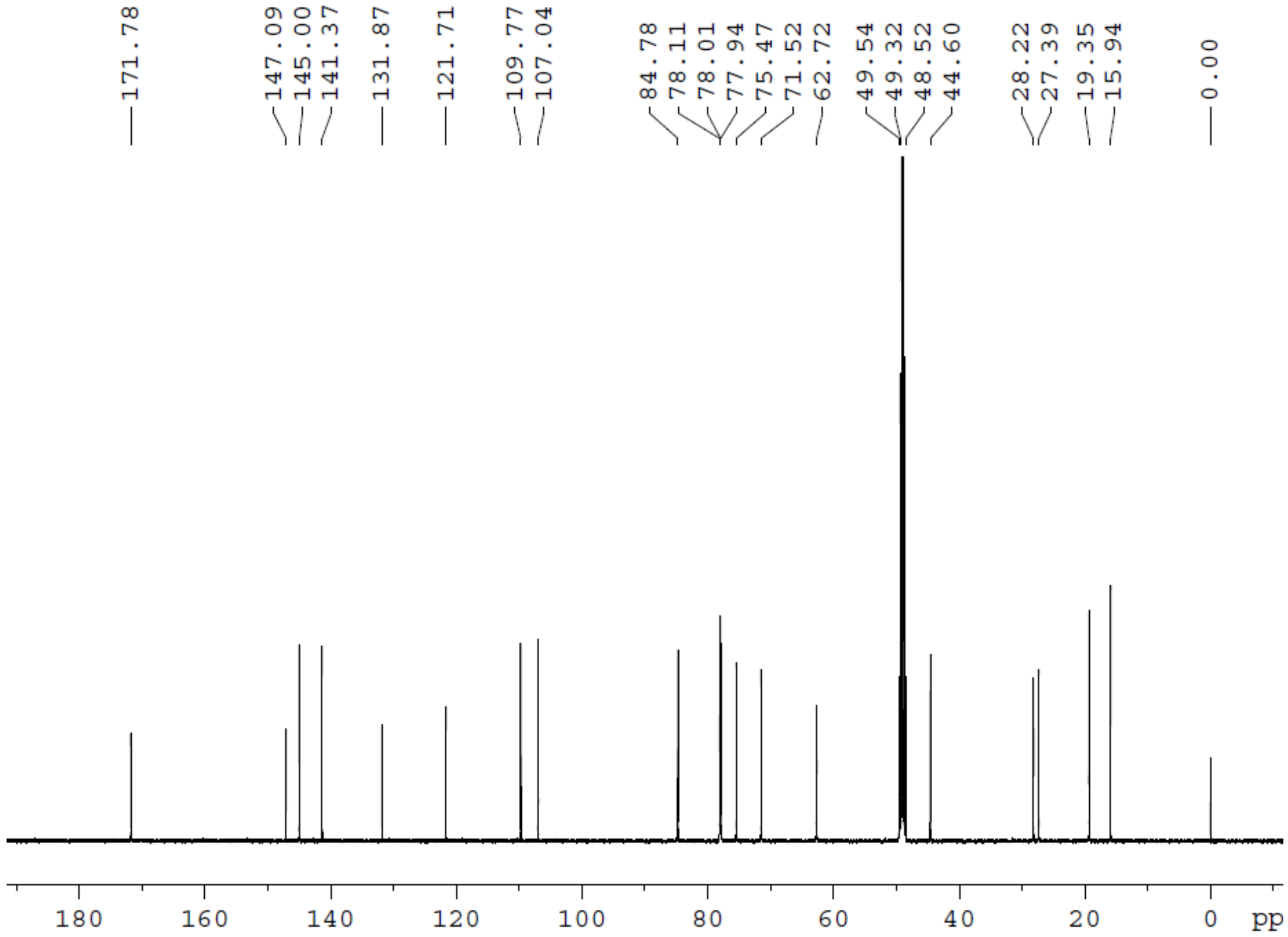

Figure S10 ^{13}C NMR (125 MHz, CD_3OD) spectrum of compound **2**

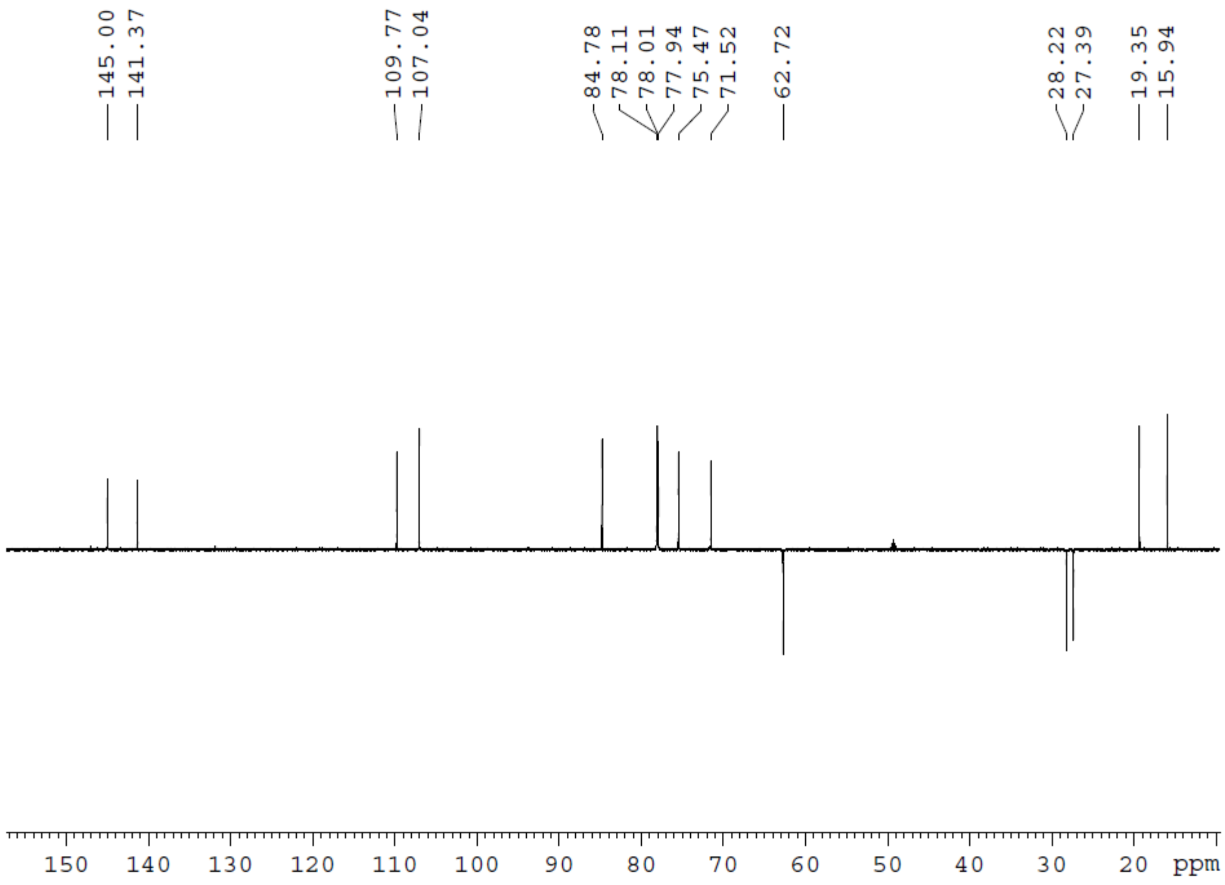

Figure S11 DEPT 135 (CD_3OD) spectrum of compound **2**

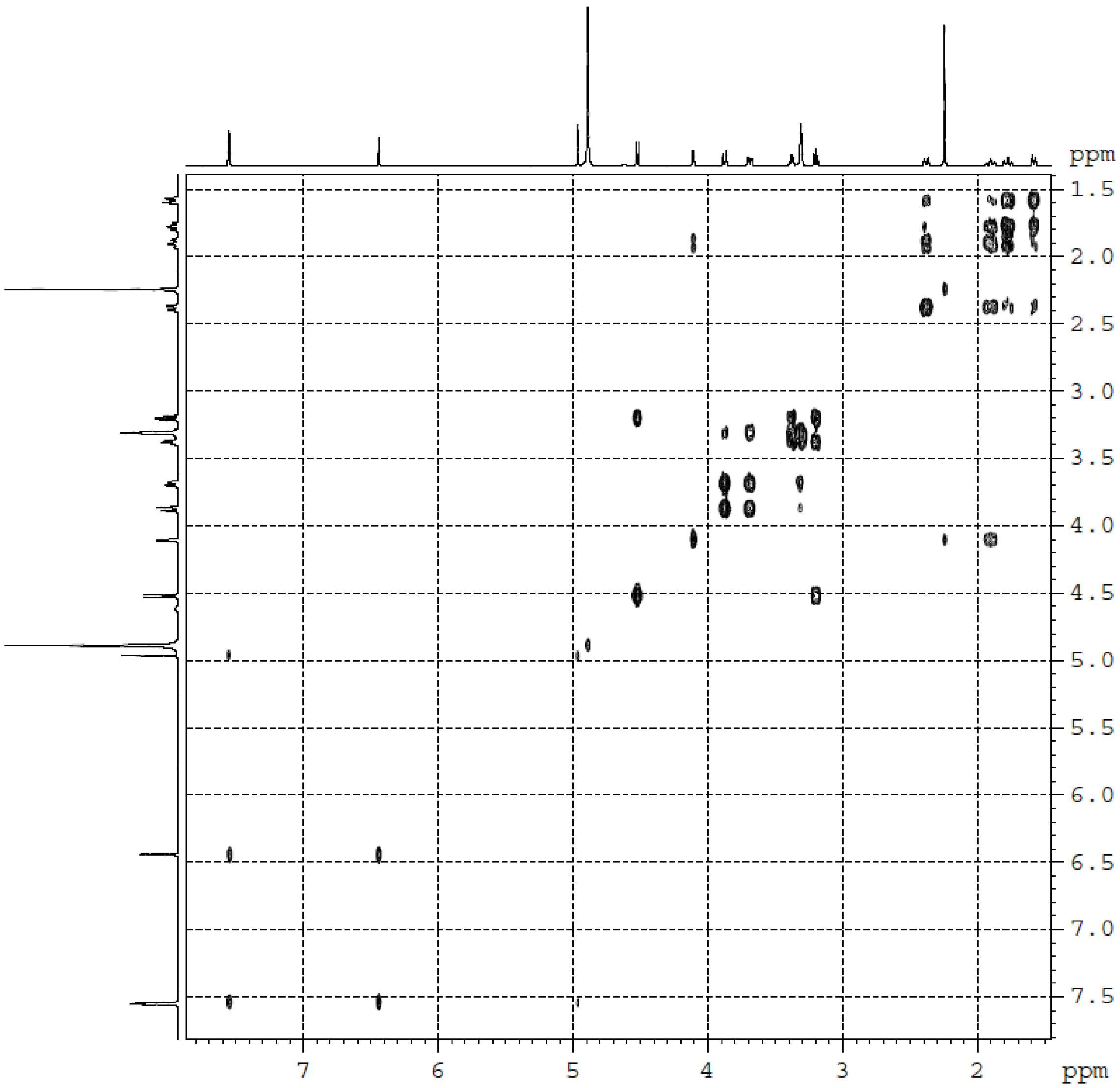

Figure S12 ^{1}H ^{1}H COSY (CD_3OD) spectrum of compound **2**

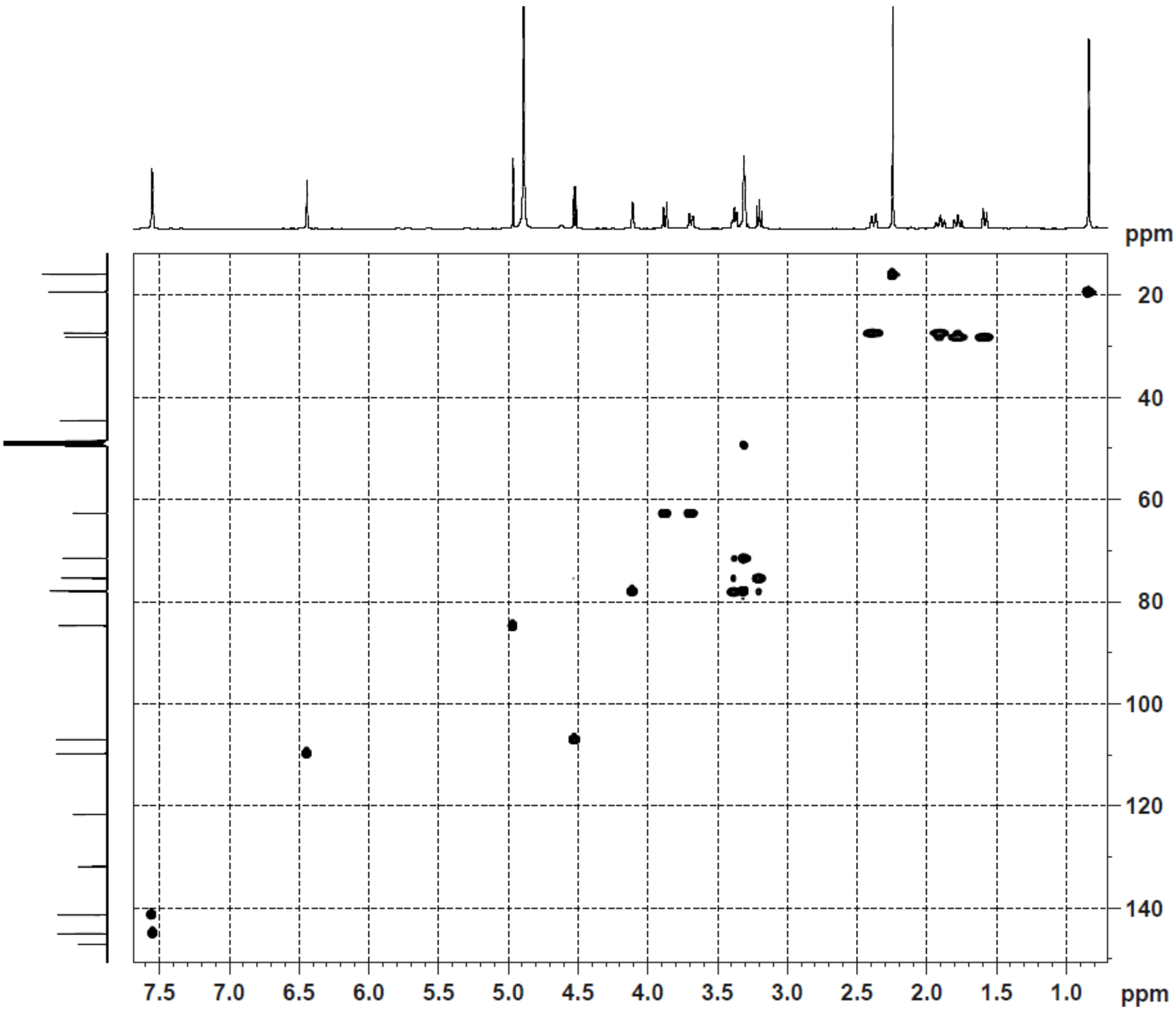

Figure S13 HSQC (CD_3OD) spectrum of compound **2**

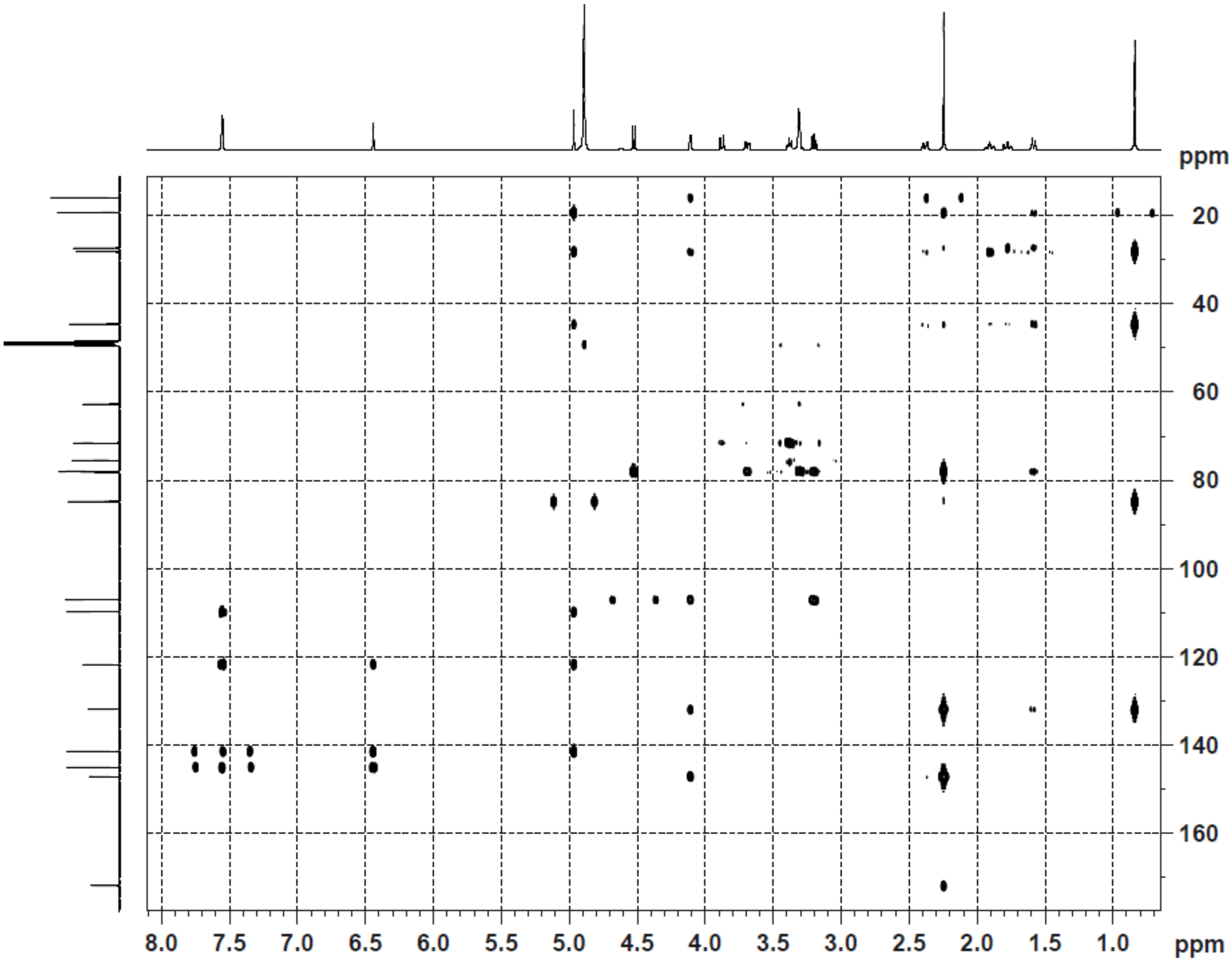

Figure S14 HMBC (CD_3OD) spectrum of compound **2**

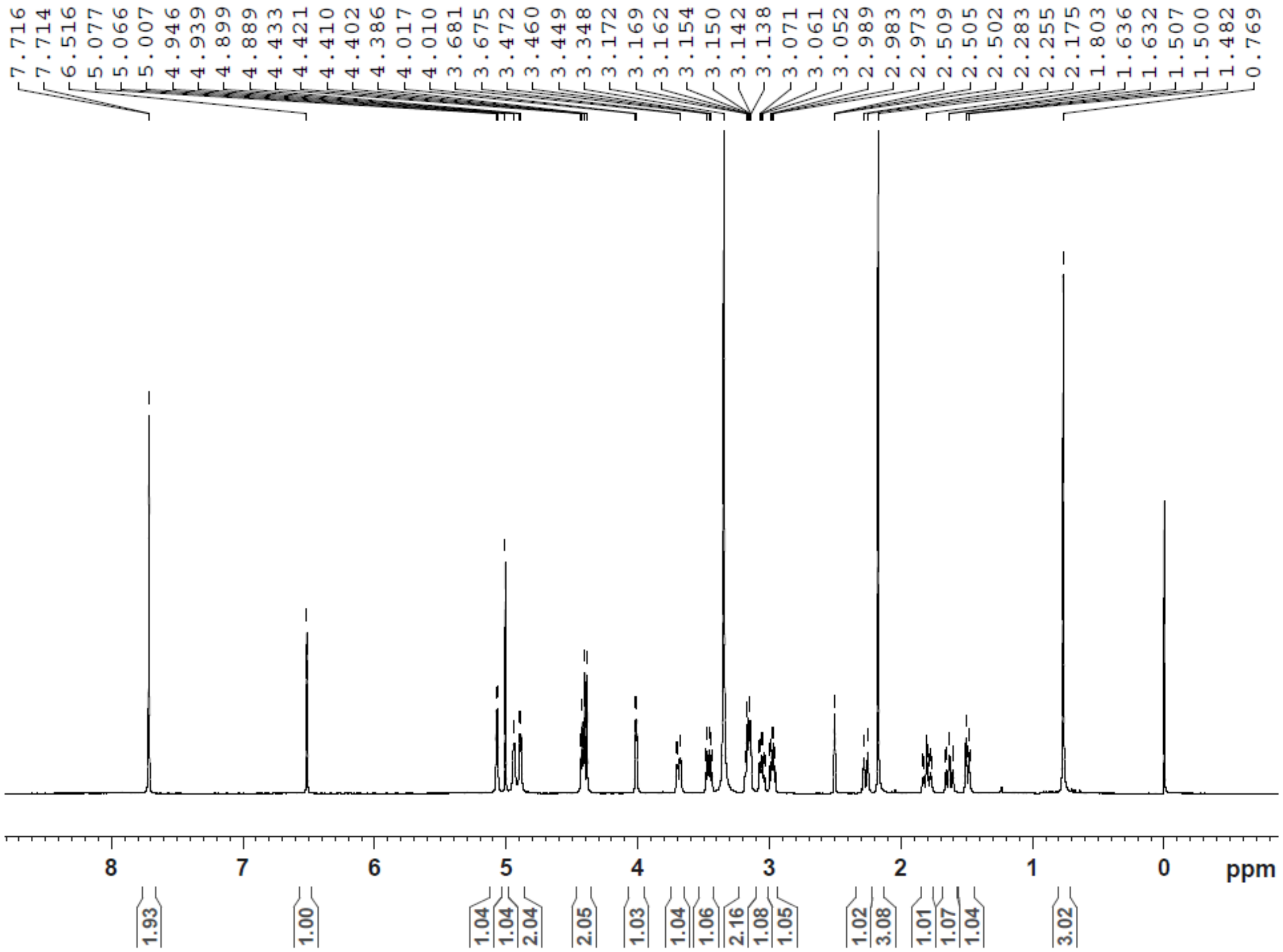

Figure S15 ^{1}H NMR (500 MHz, DMSO-d_6) spectrum of compound **2**

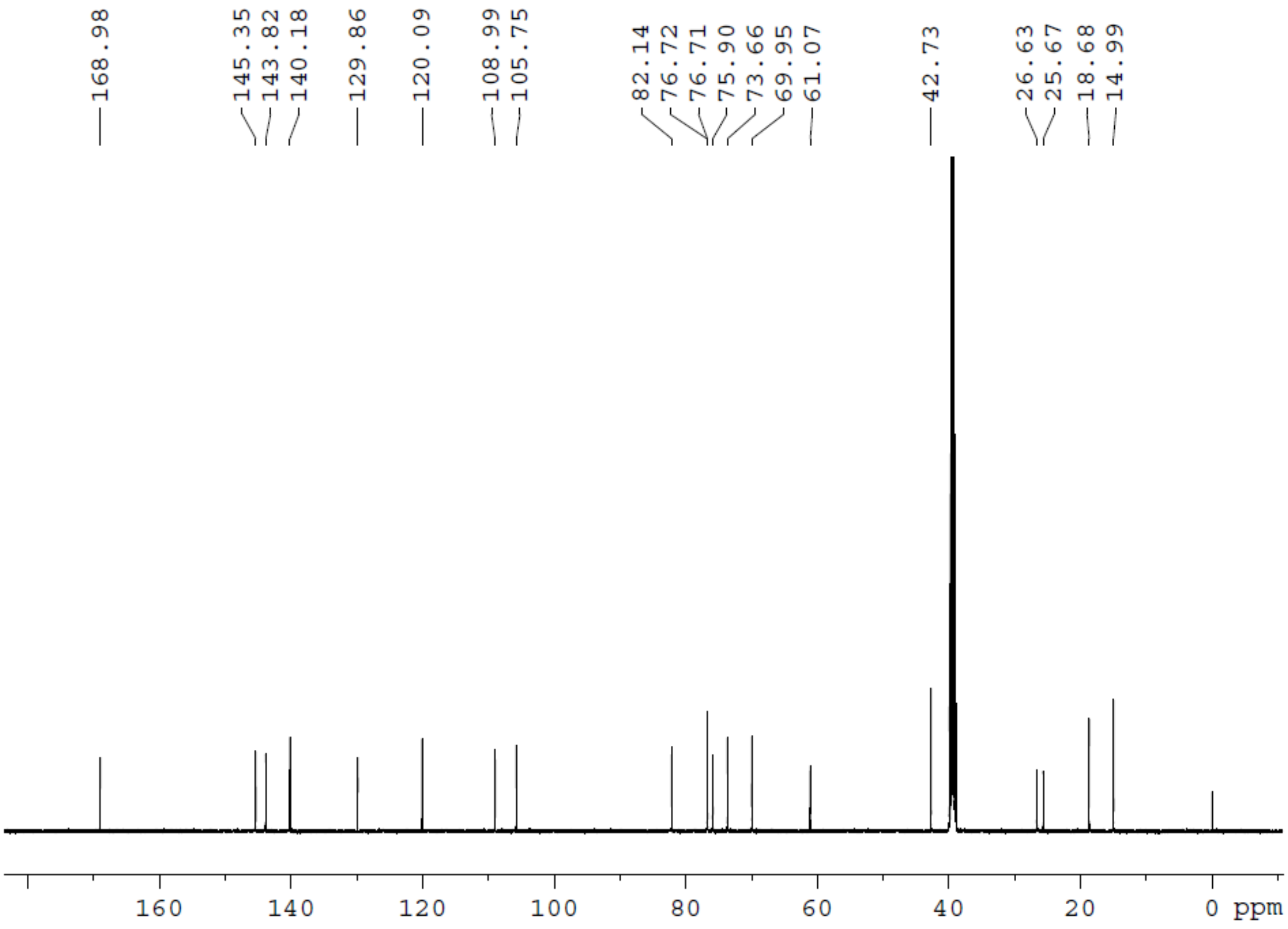

Figure S16 ^{13}C NMR (125 MHz, DMSO-d_6) spectrum of compound **2**

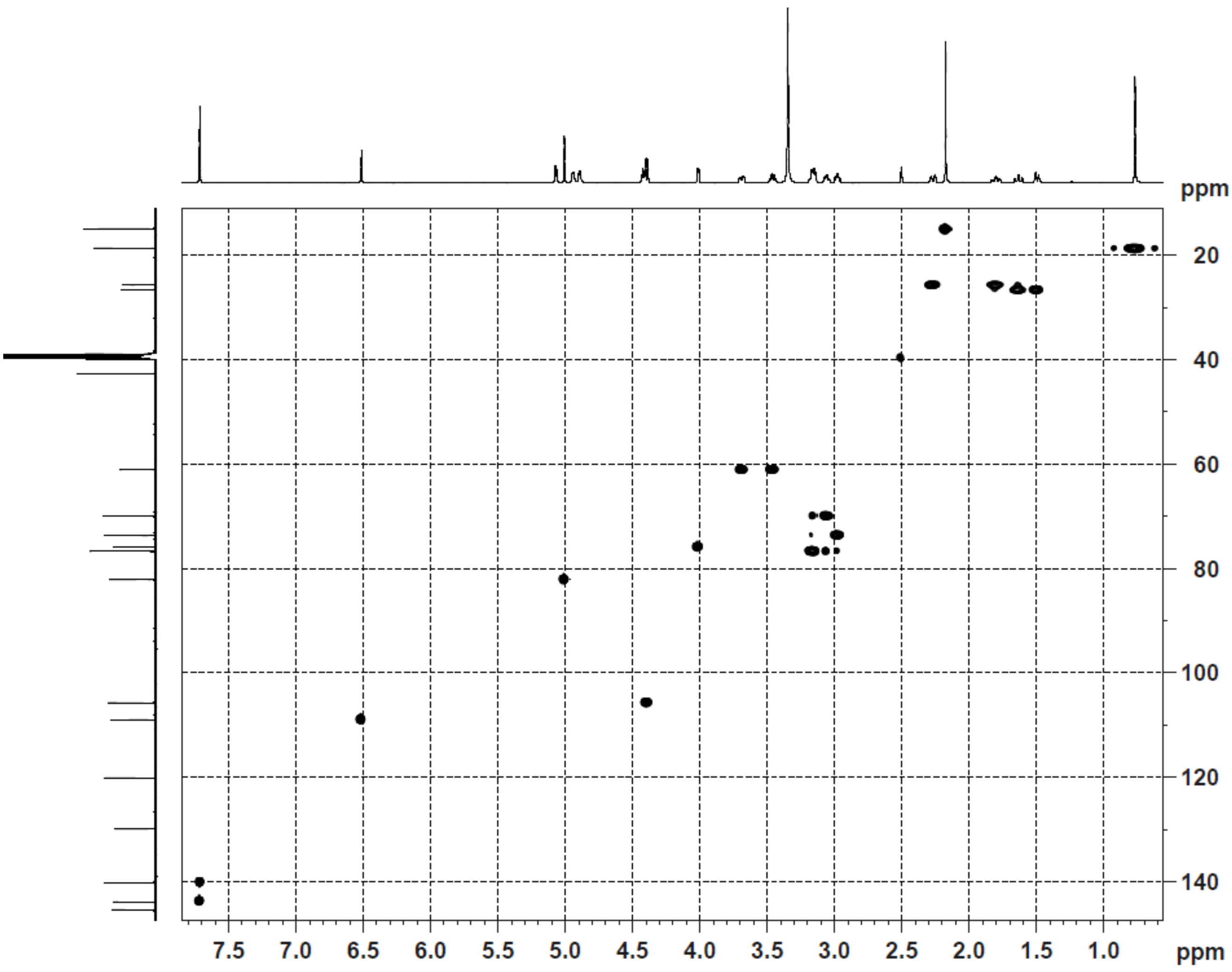

Figure S17 HSQC (DMSO-d_6) spectrum of compound **2**

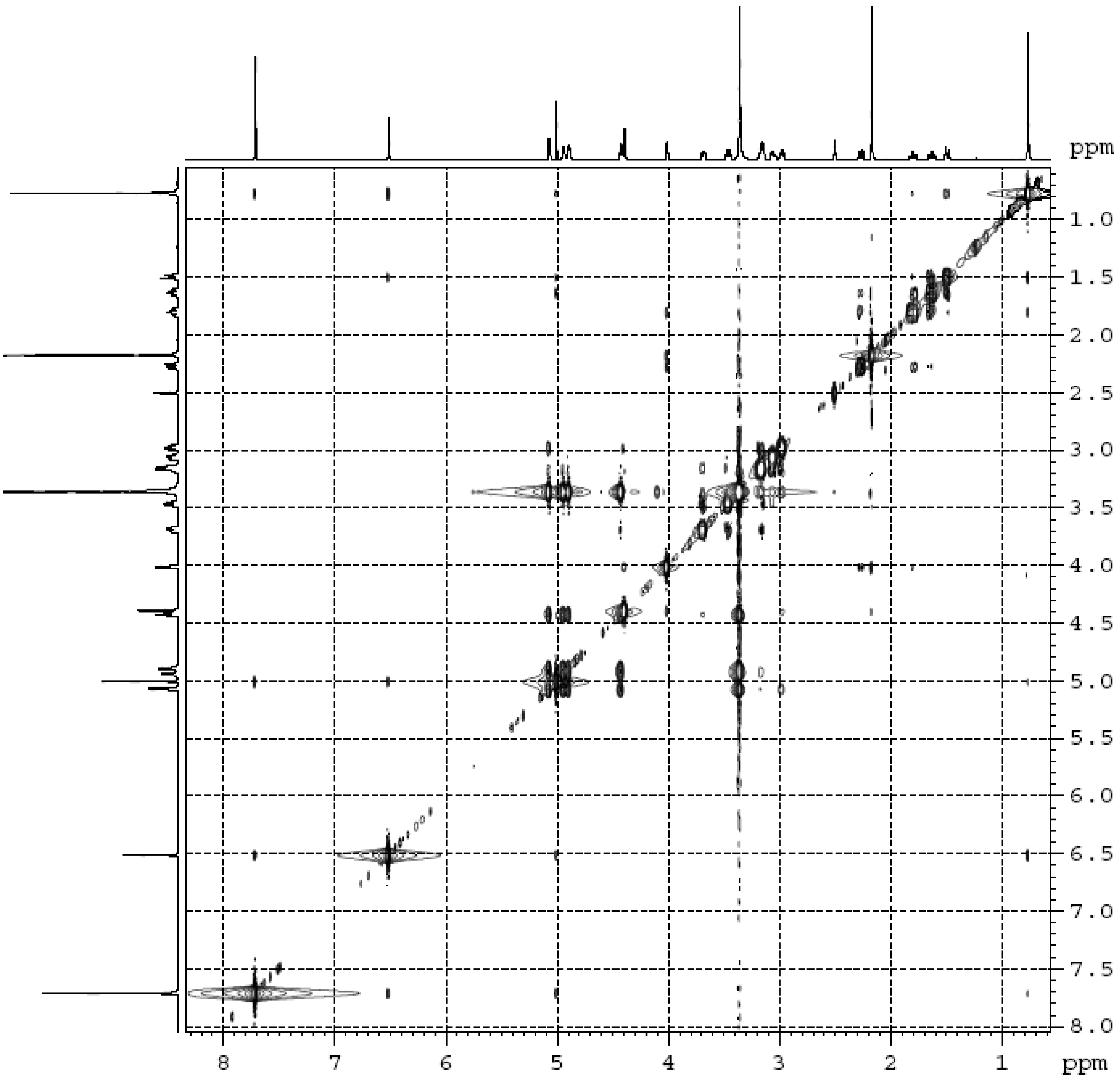

Figure S18 NOESY (DMSO-d_6) spectrum of compound **2**

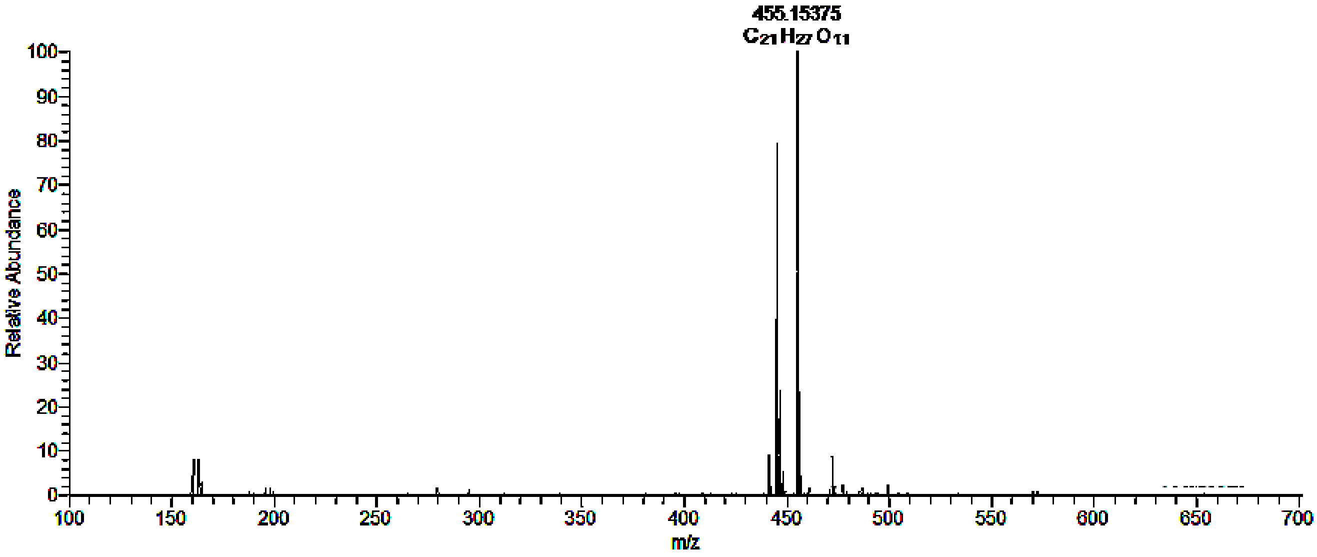

Figure S19 HRESI-Orbitrap-MS spectrum of compound **2**

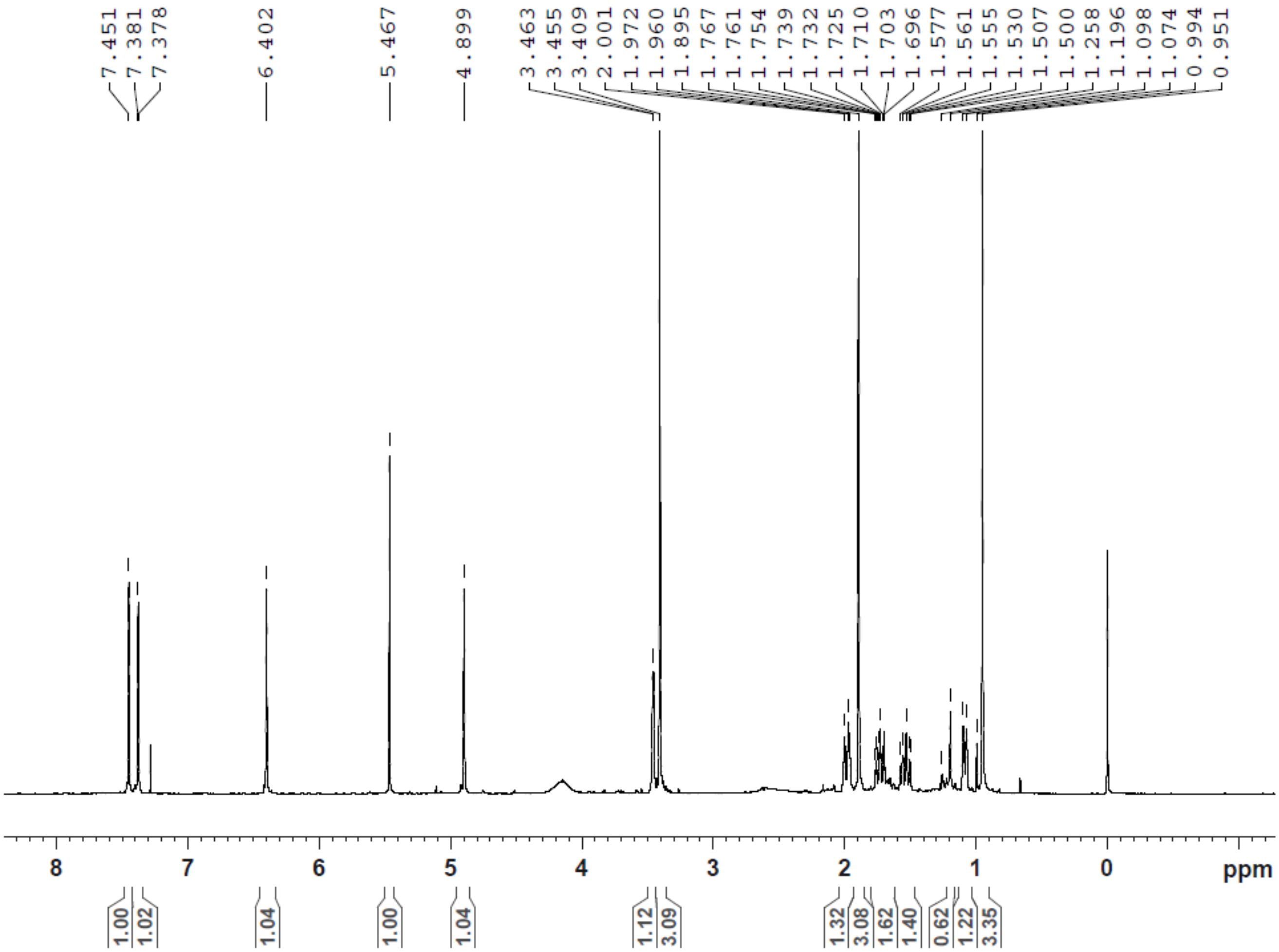

Figure S20 ^{1}H NMR (500 MHz, $CDCl_3$) spectrum of compound **3**

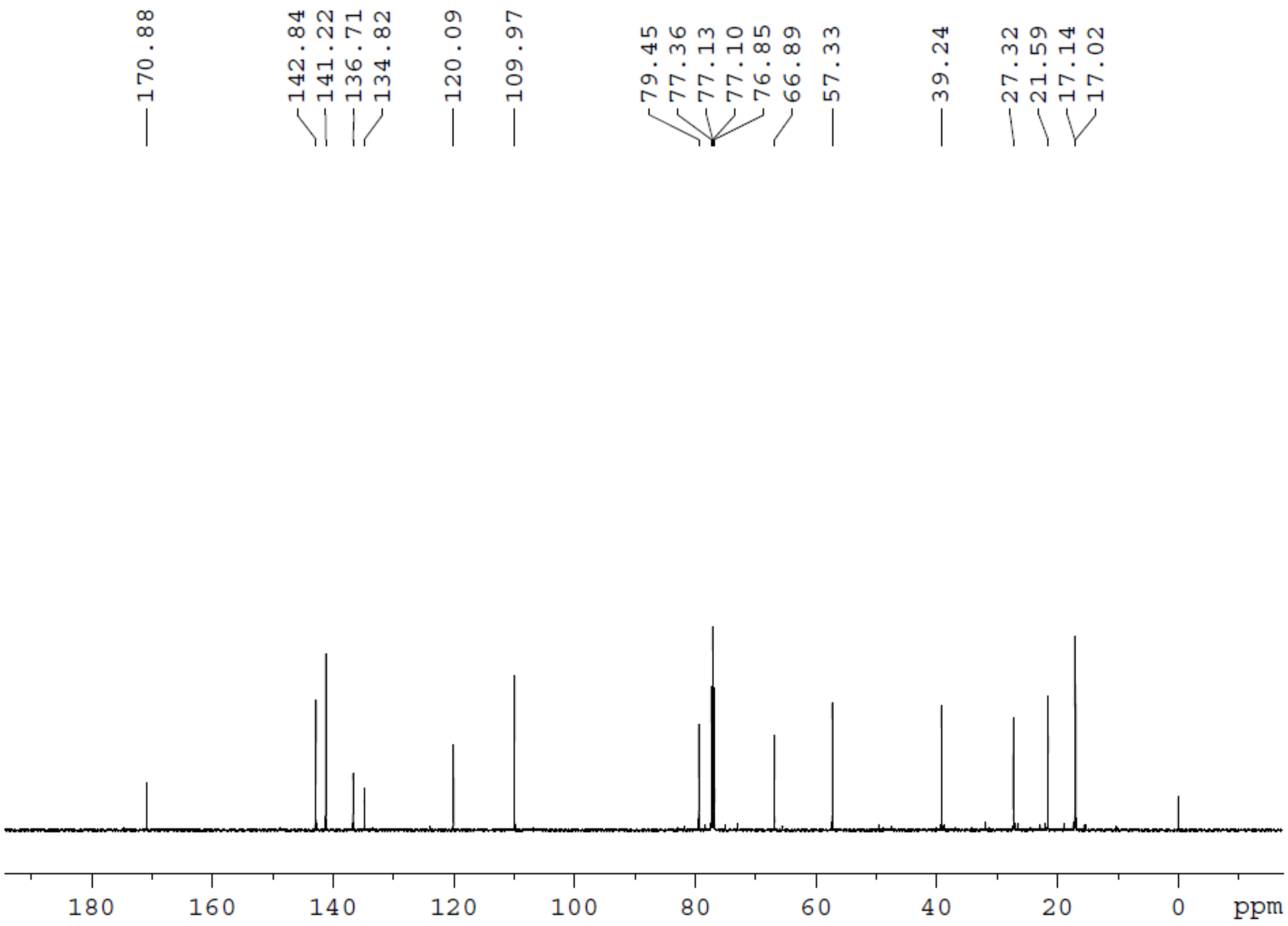

Figure S21 ^{13}C NMR (125 MHz, $CDCl_3$) spectrum of compound **3**

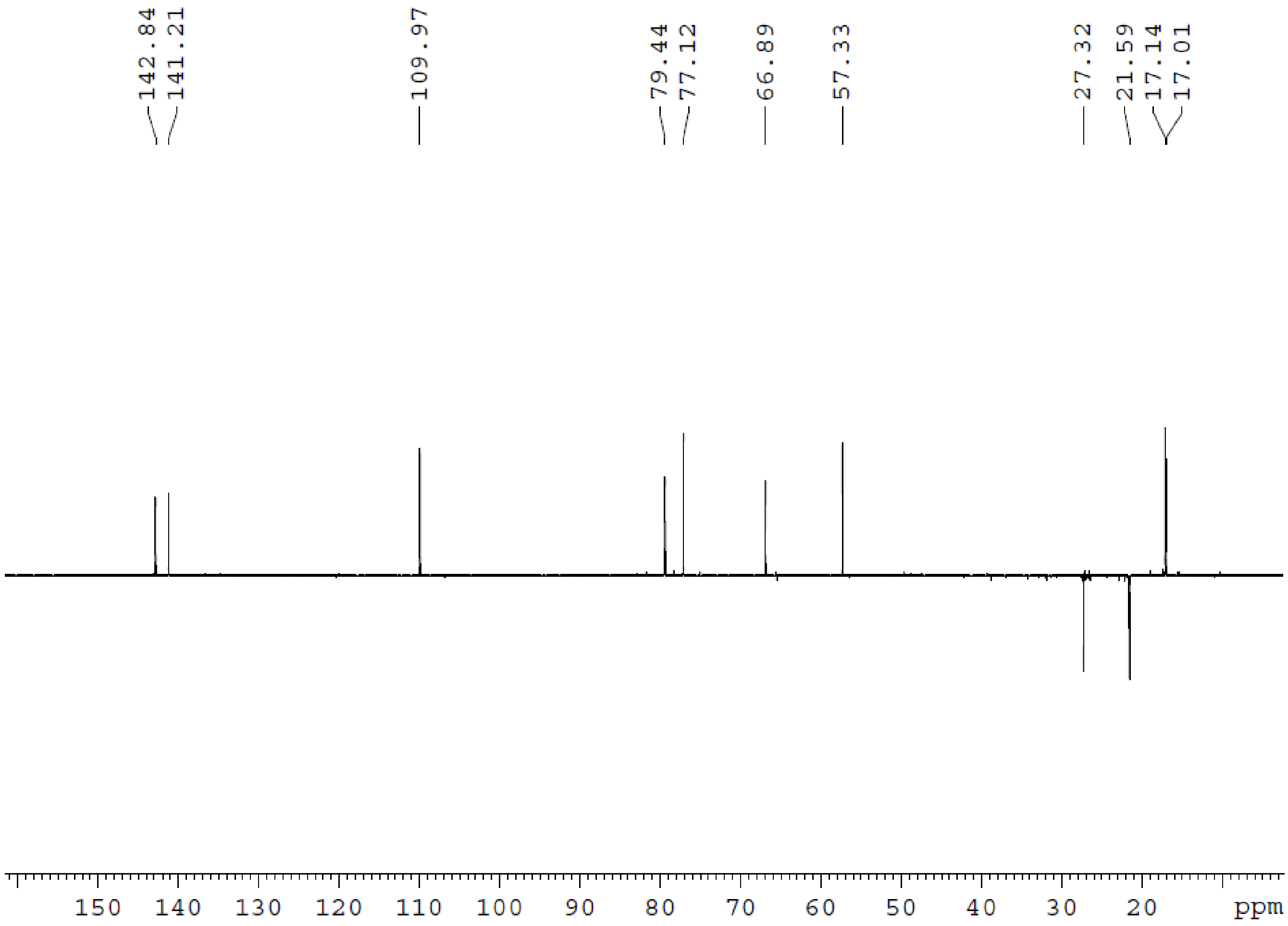

Figure S22 DEPT 135 ($CDCl_3$) spectrum of compound **3**

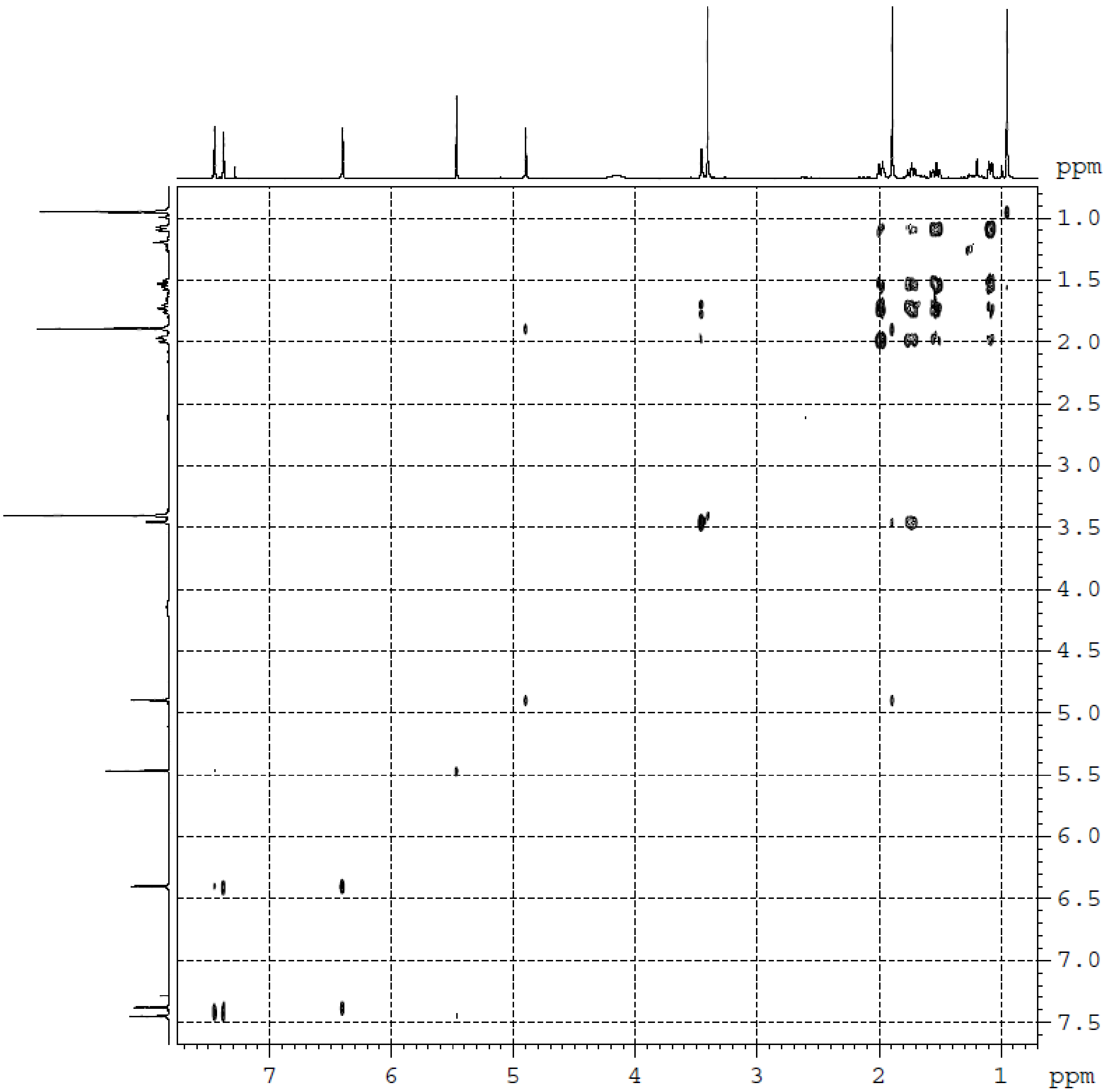

Figure S23 ^{1}H ^{1}H COSY ($CDCl_3$) spectrum of compound **3**

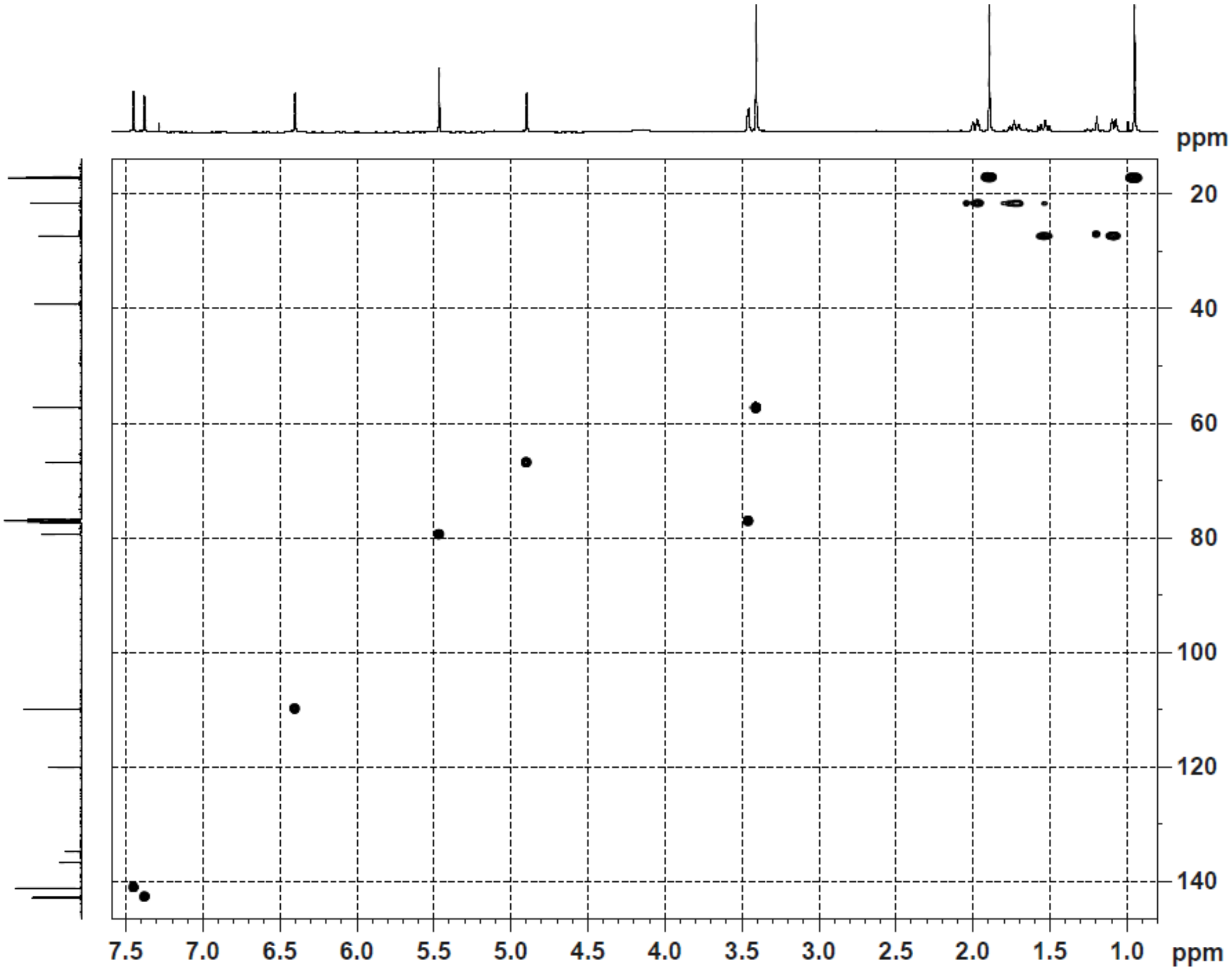

Figure S24 HSQC ($CDCl_3$) spectrum of compound **3**

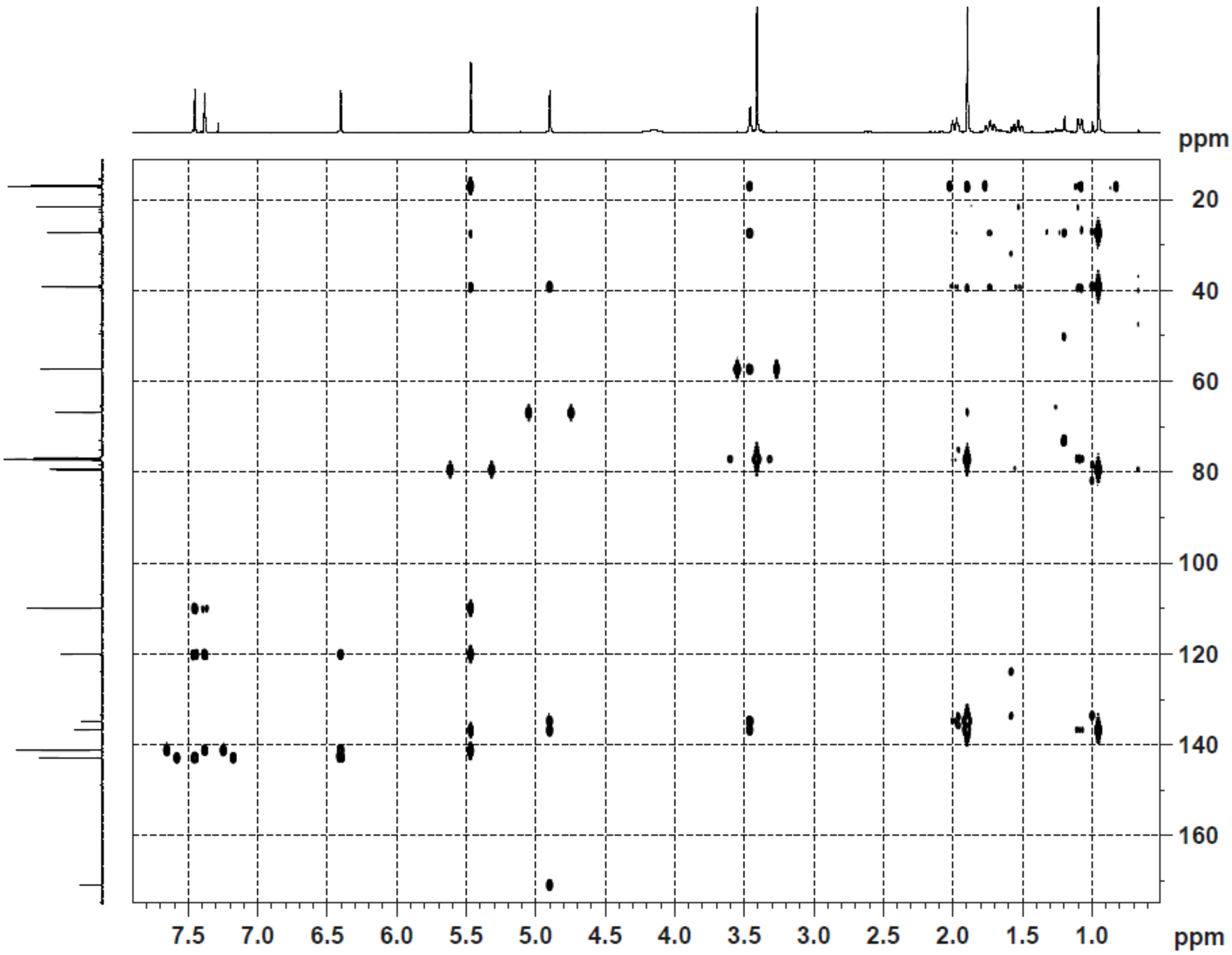

Figure S25 HMBC ($CDCl_3$) spectrum of compound **3**

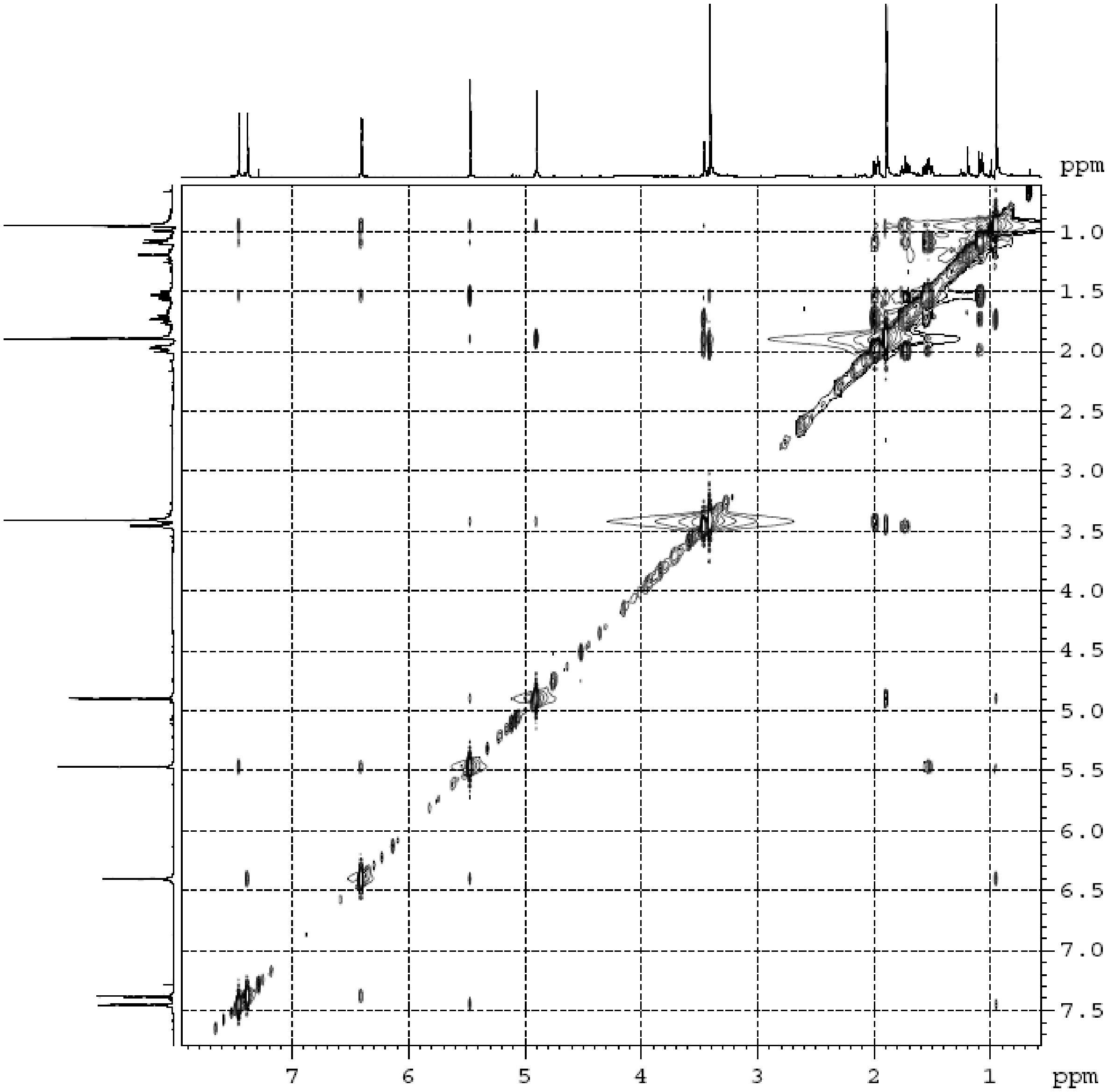

Figure S26 NOSEY ($CDCl_3$) spectrum of compound **3**

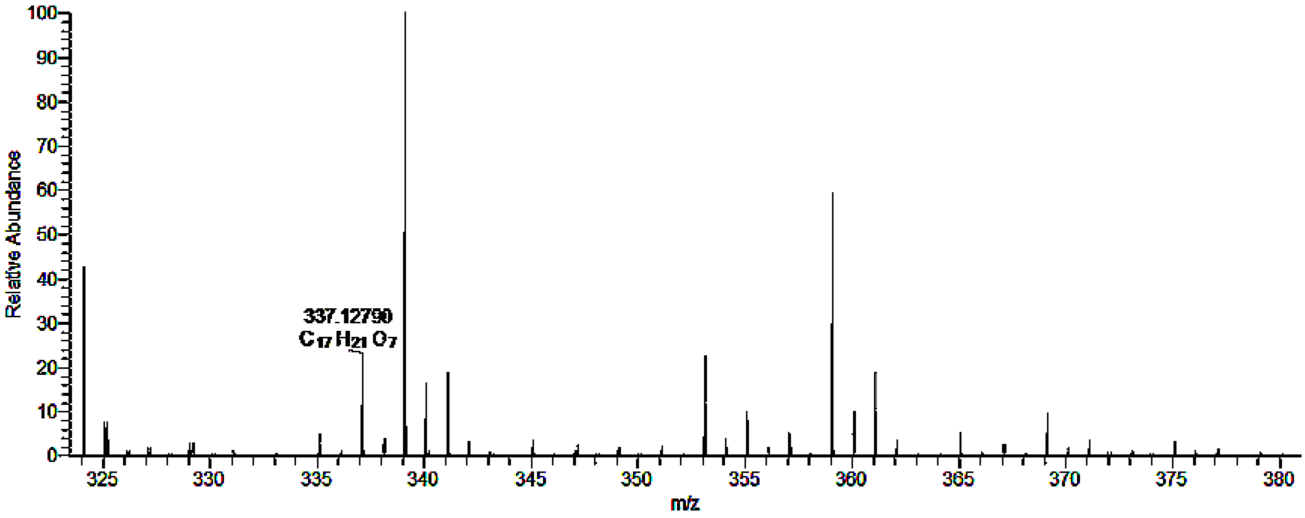

Figure S27 HRESI-Orbitrap-MS spectrum of compound **3**

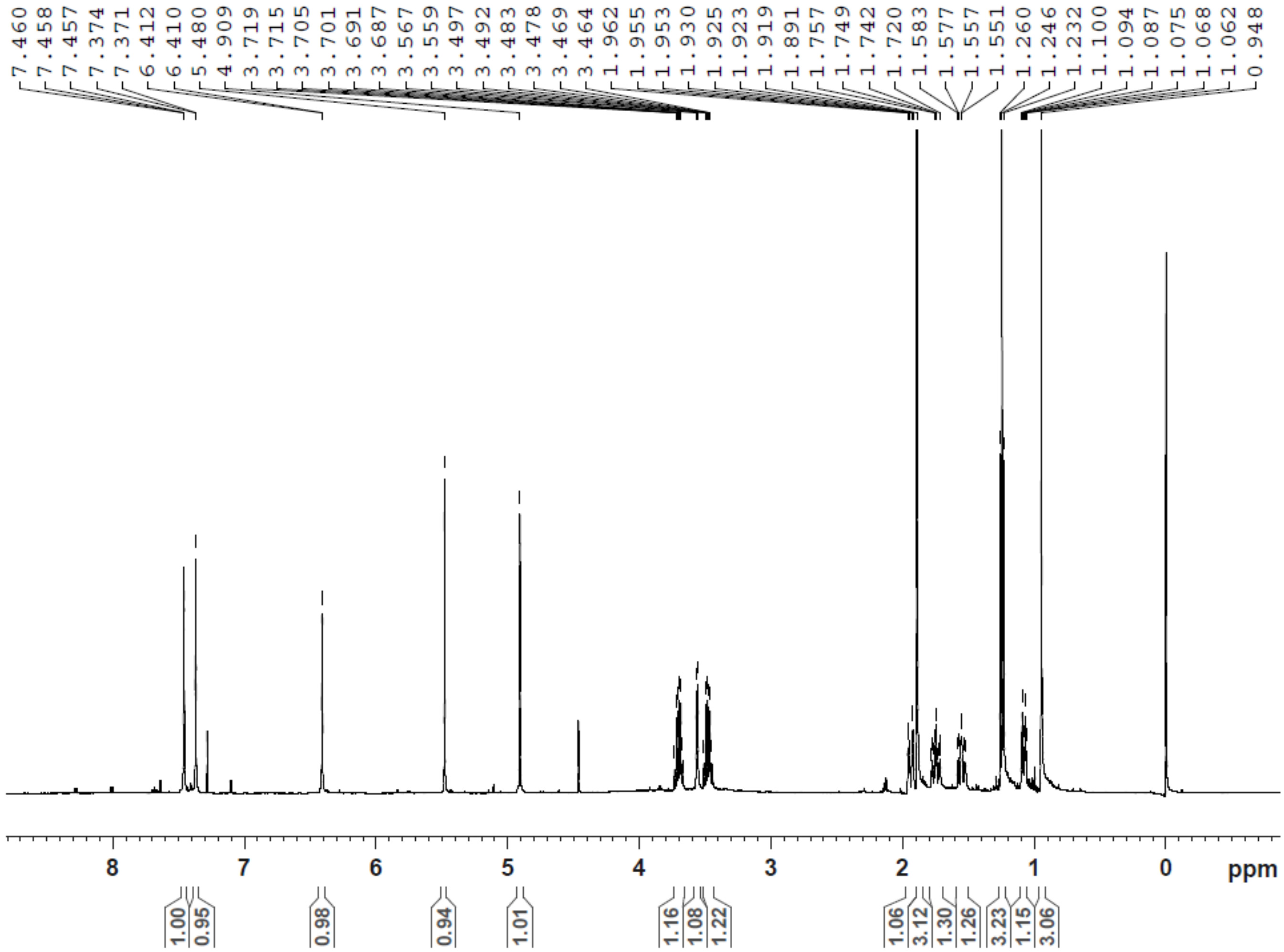

Figure S28 ^{1}H NMR (500 MHz, $CDCl_3$) spectrum of compound **4**

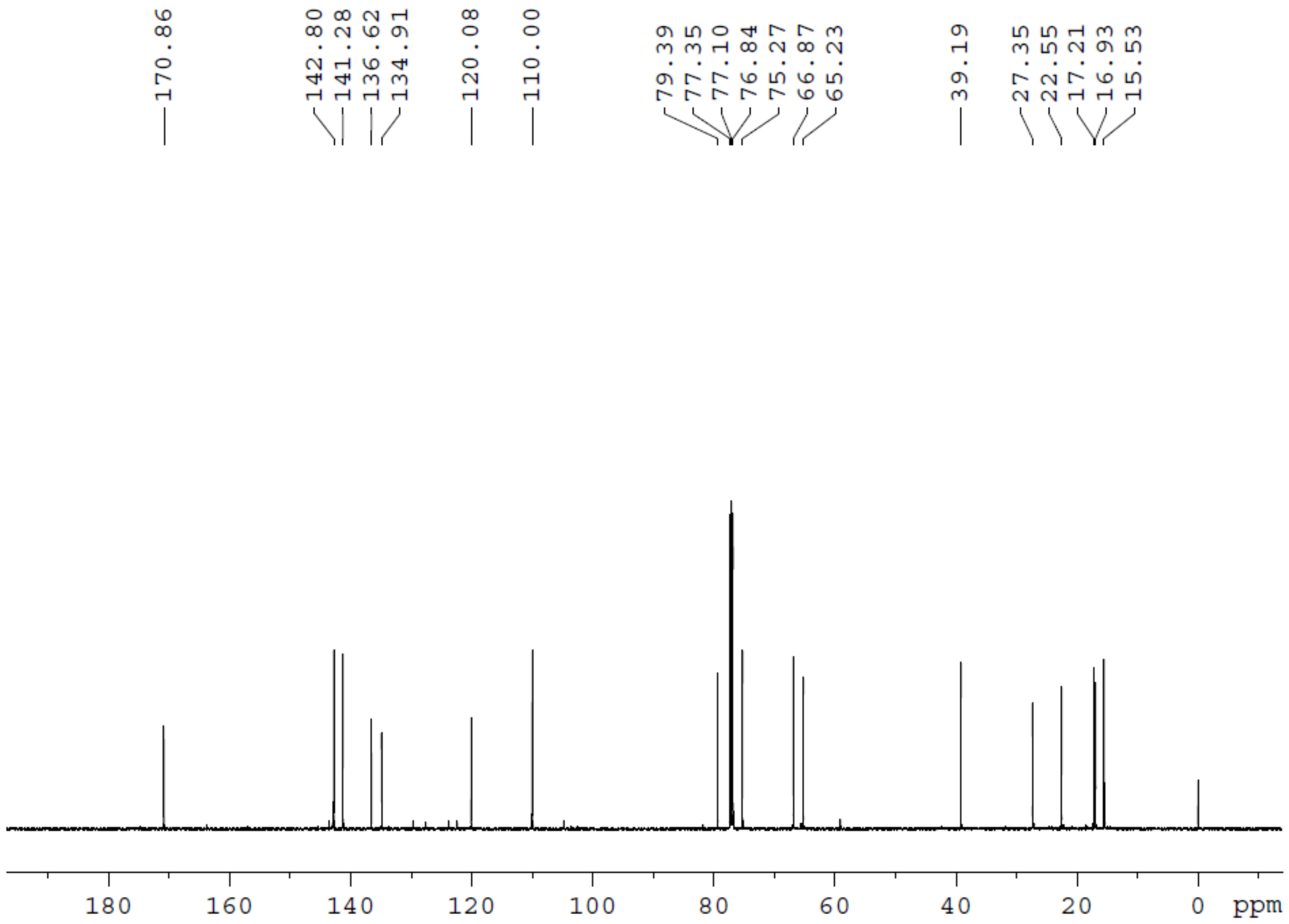

Figure S29 ^{13}C NMR (125 MHz, $CDCl_3$) spectrum of compound **4**

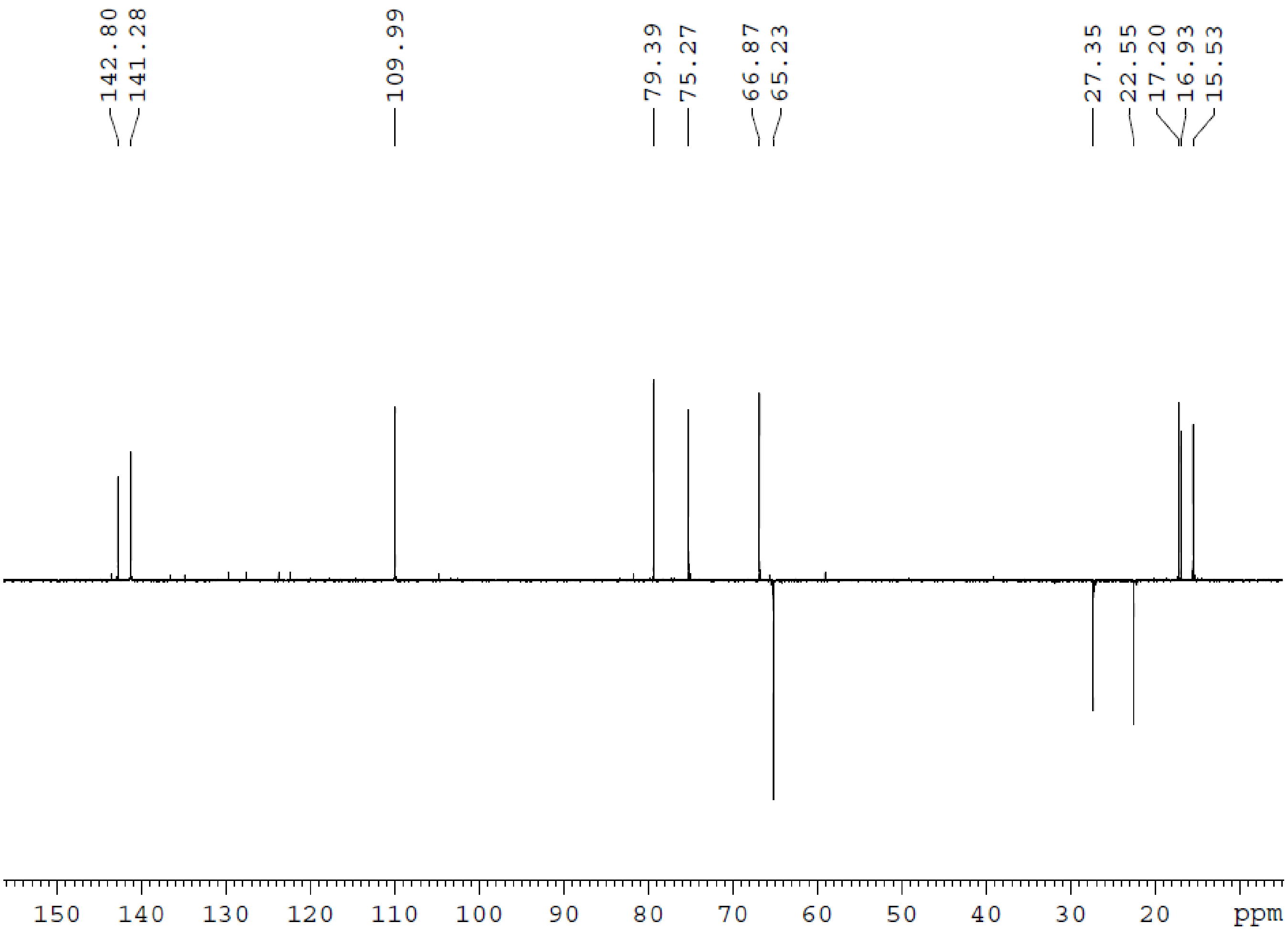

Figure S30 DEPT 135 ($CDCl_3$) spectrum of compound **4**

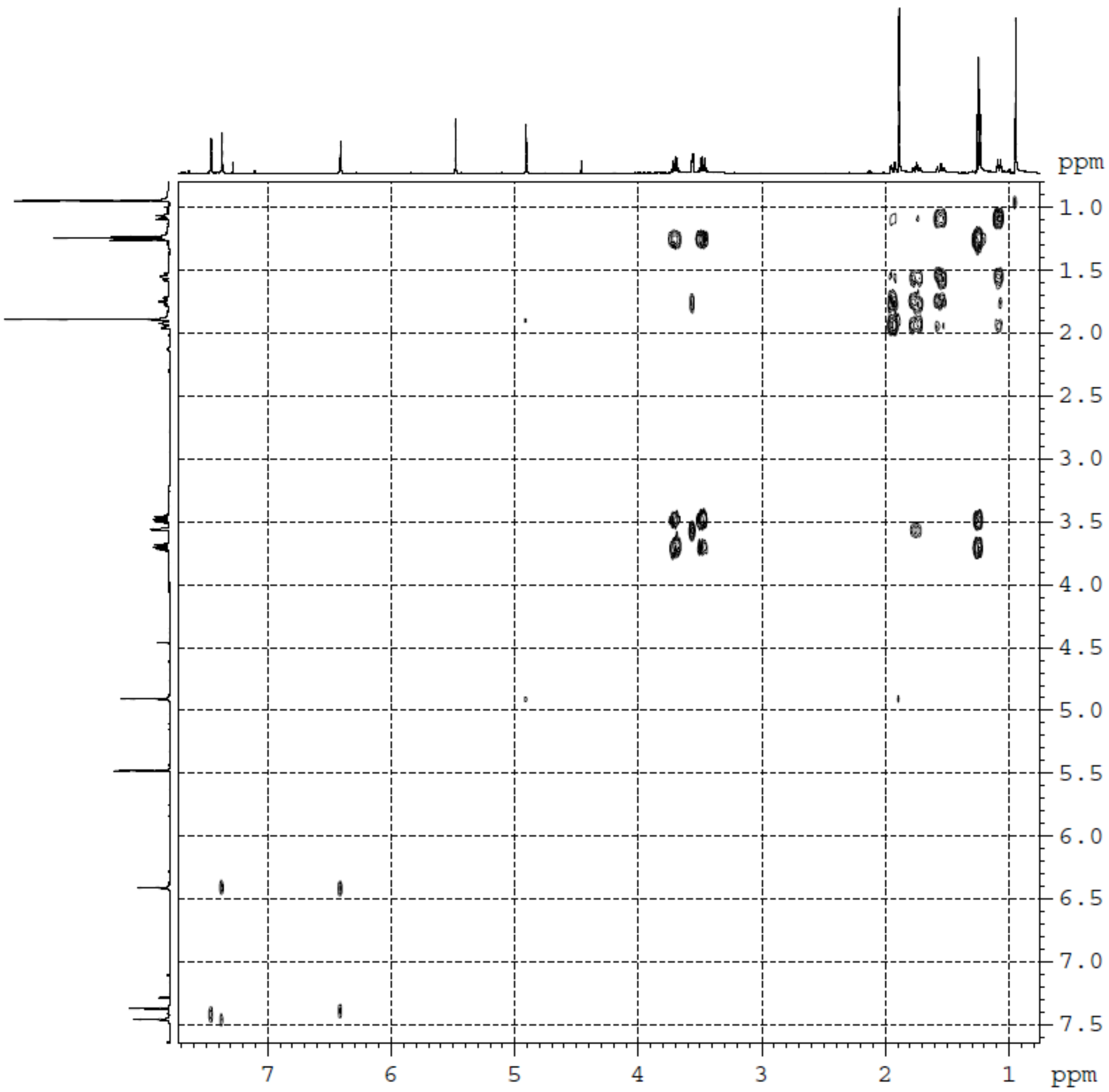

Figure S31 ^{1}H ^{1}H COSY ($CDCl_3$) spectrum of compound **4**

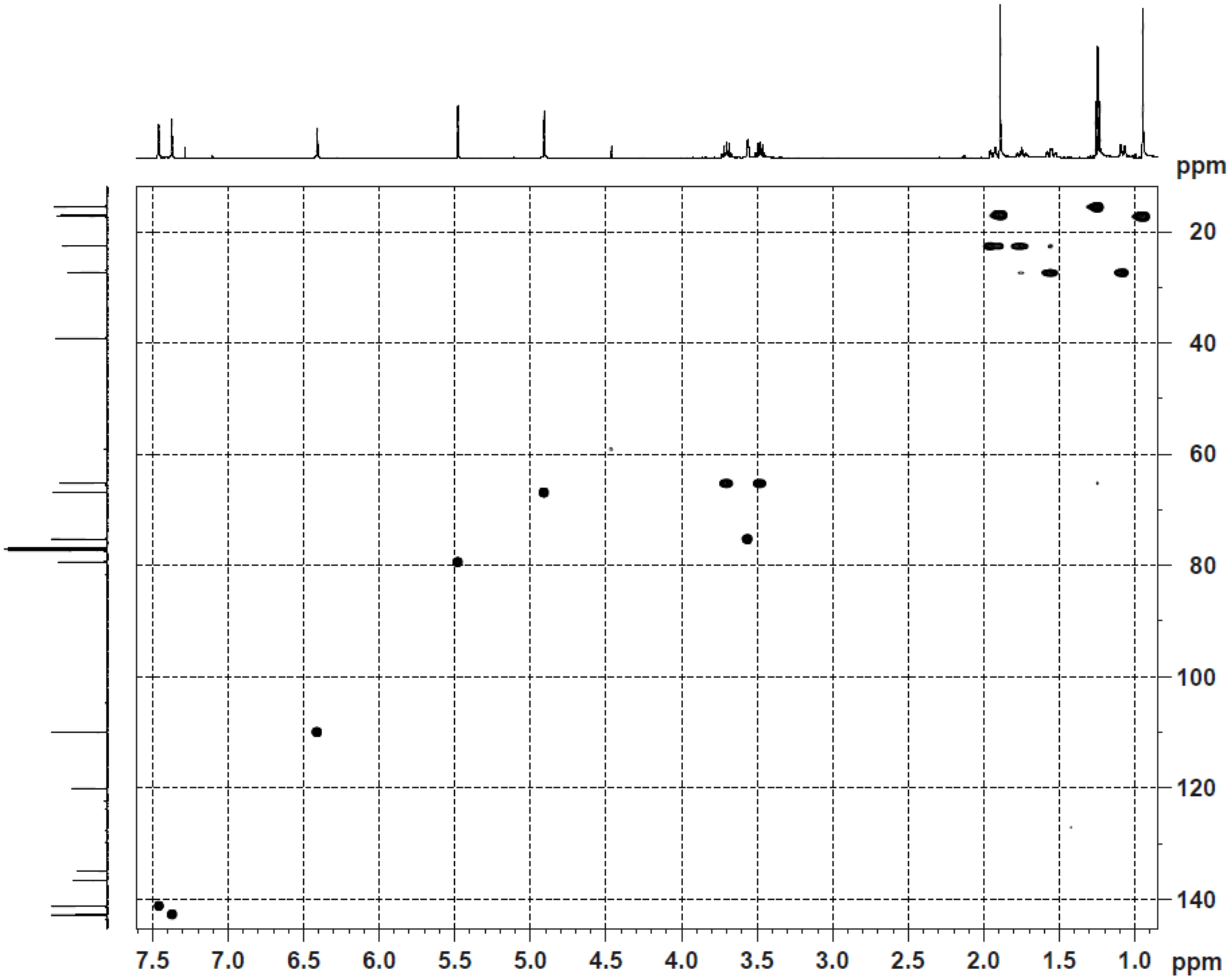

Figure S32 HSQC ($CDCl_3$) spectrum of compound **4**

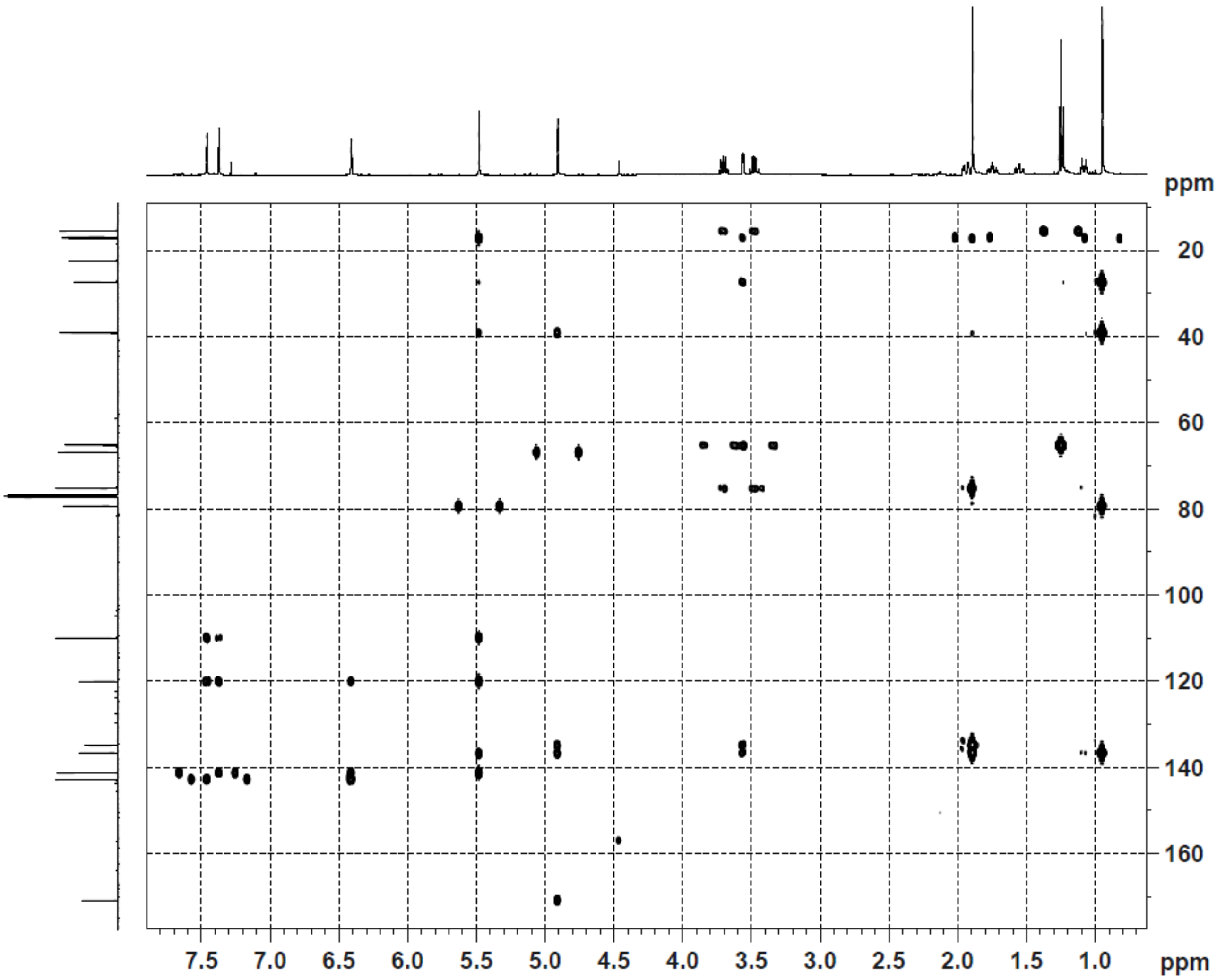

Figure S33 HMBC ($CDCl_3$) spectrum of compound **4**

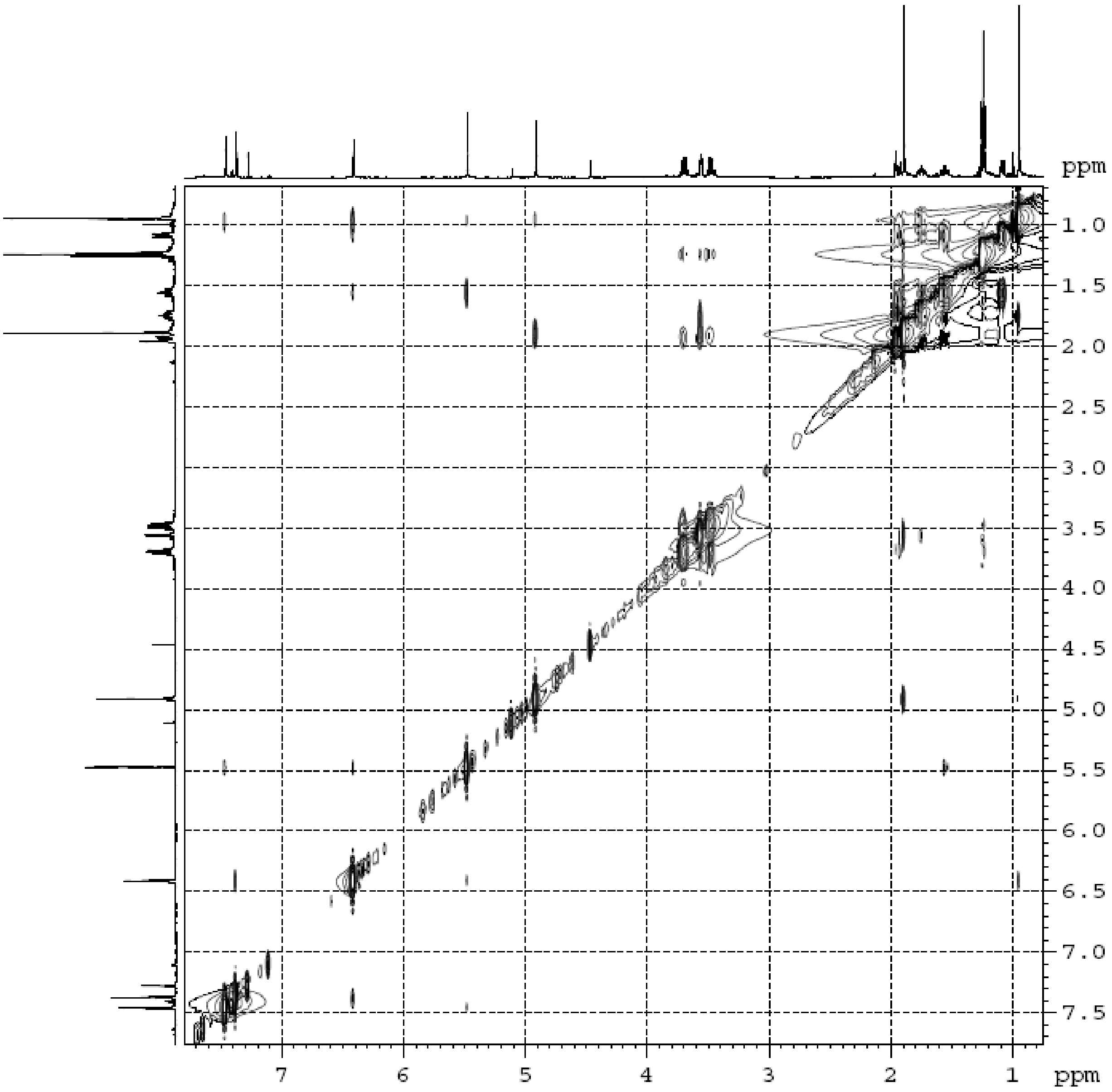

Figure S34 NOESY ($CDCl_3$) spectrum of compound **4**

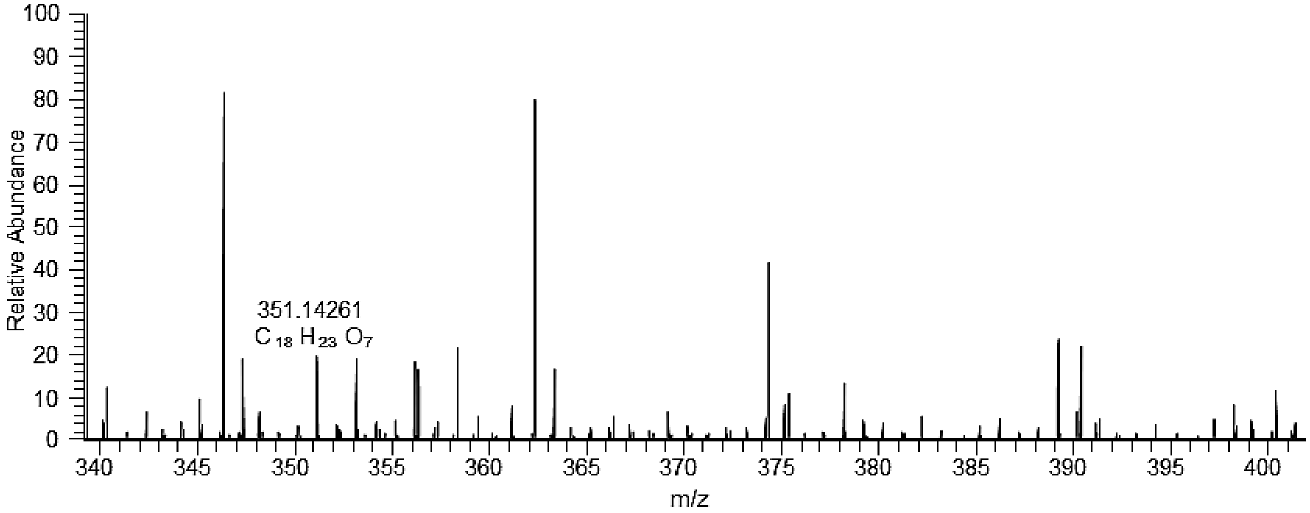

Figure S35 HRESI-Orbitrap-MS spectrum of compound **4**

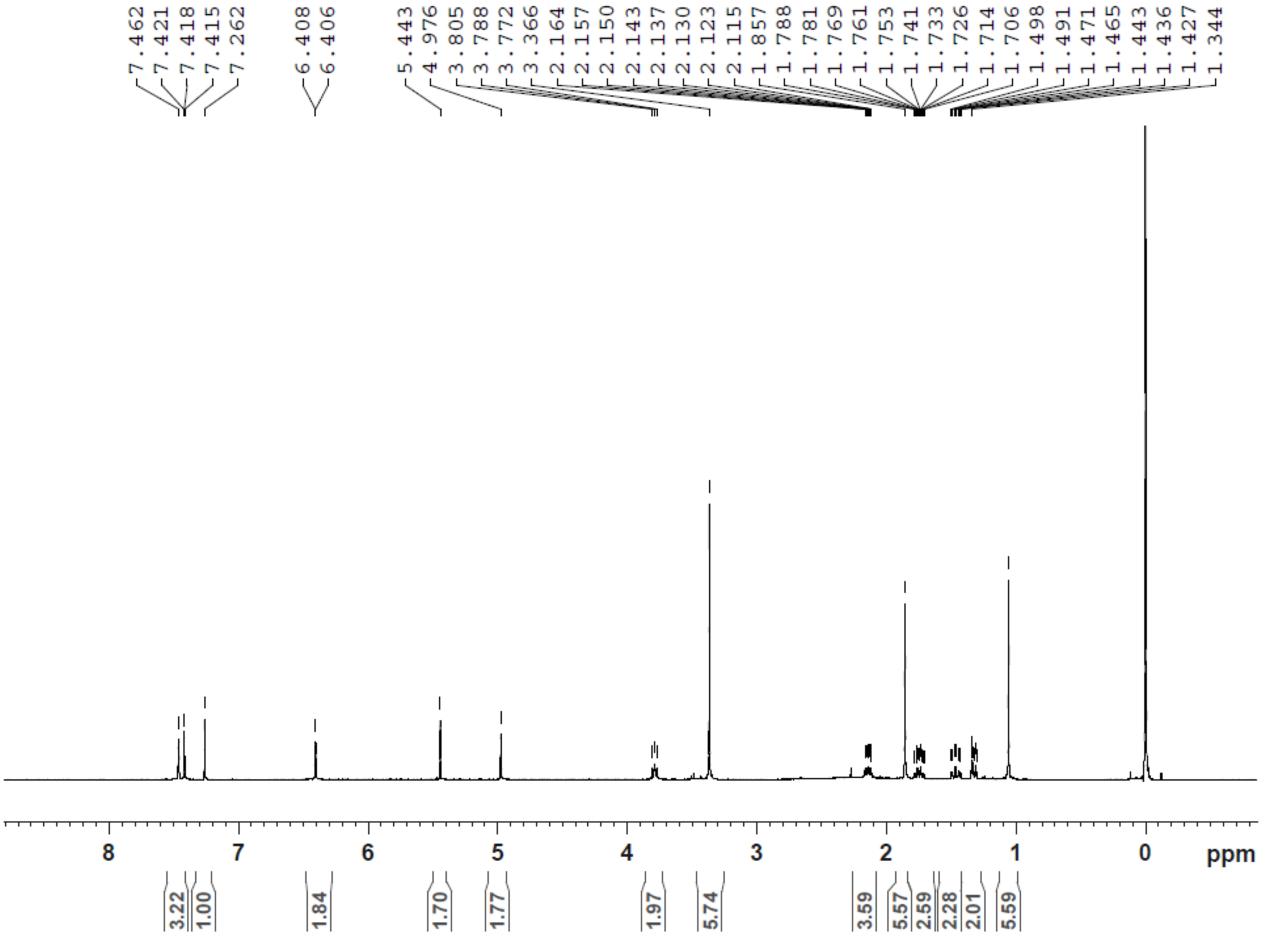

Figure S36 ^{1}H NMR (500 MHz, $CDCl_3$) spectrum of compound **5**

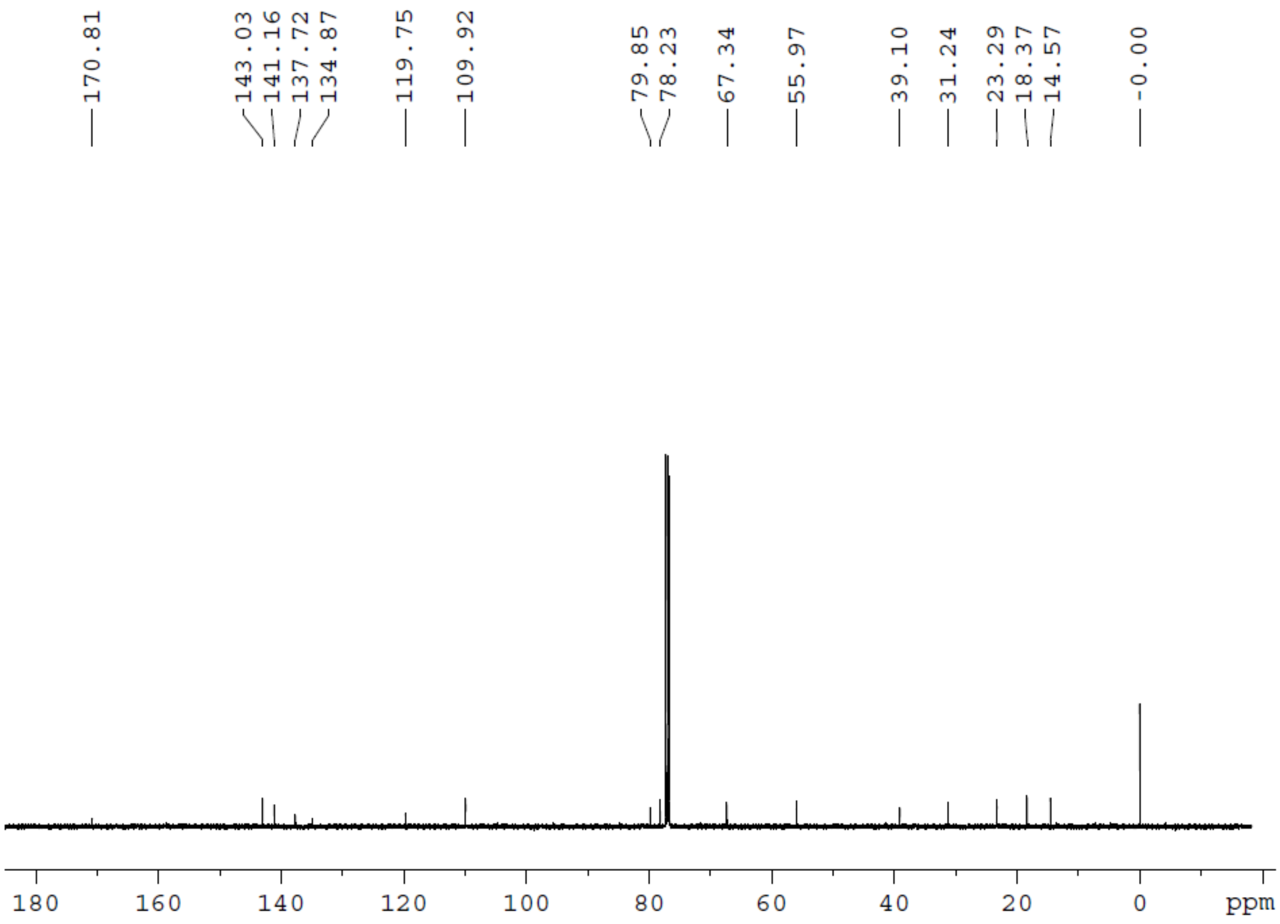

Figure S37 ^{13}C NMR (125 MHz, $CDCl_3$) spectrum of compound **5**

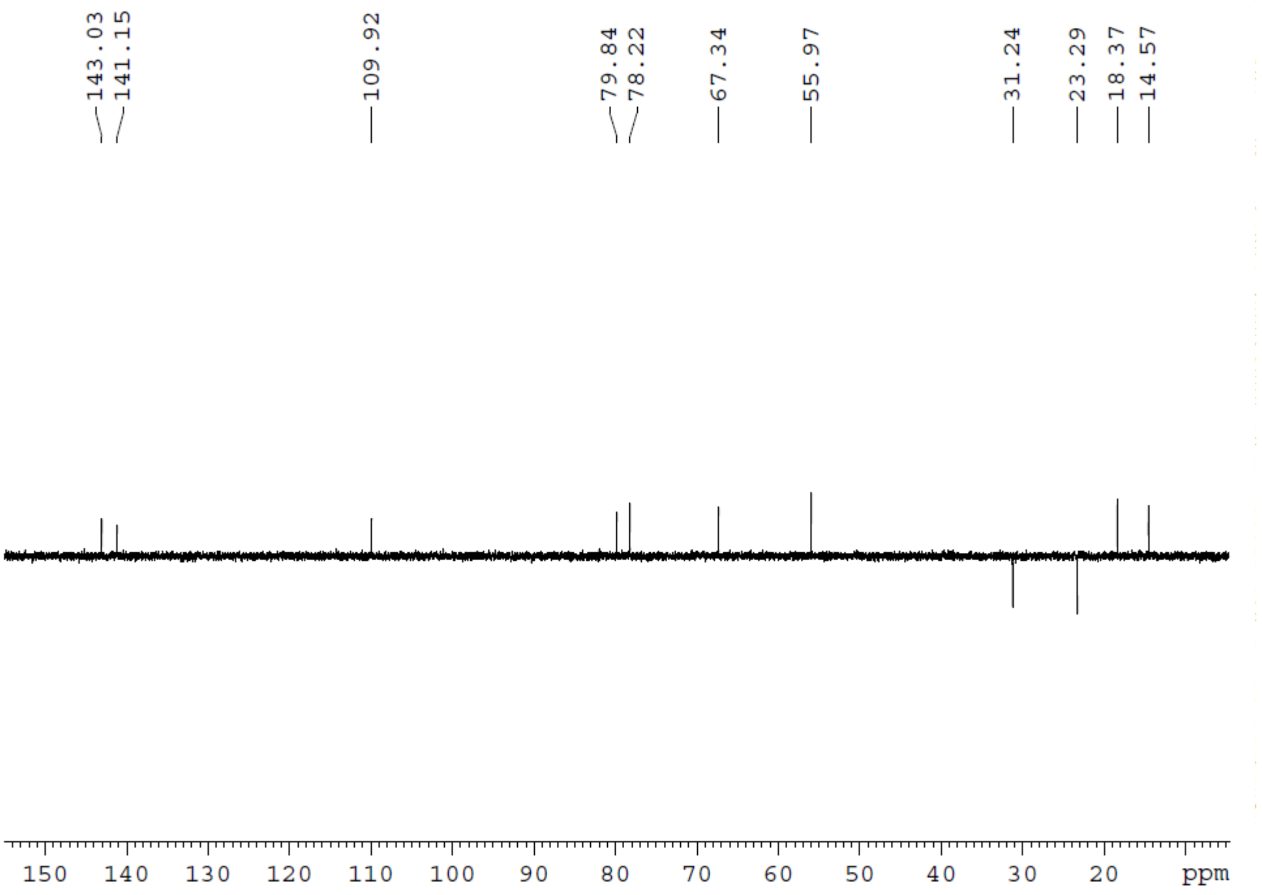

Figure S38 DEPT 135 ($CDCl_3$) spectrum of compound **5**

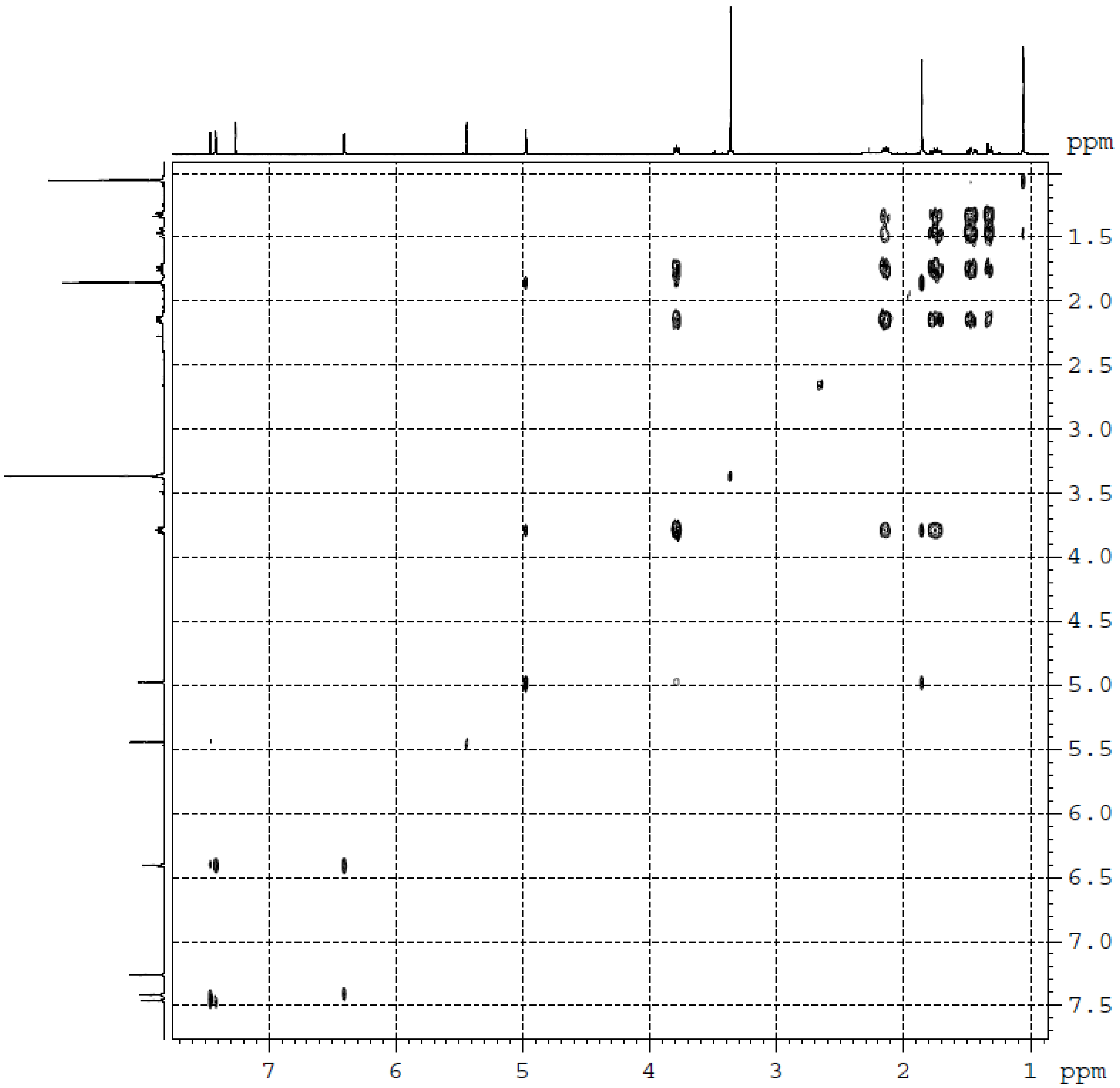

Figure S39 ^{1}H ^{1}H COSY ($CDCl_3$) spectrum of compound **5**

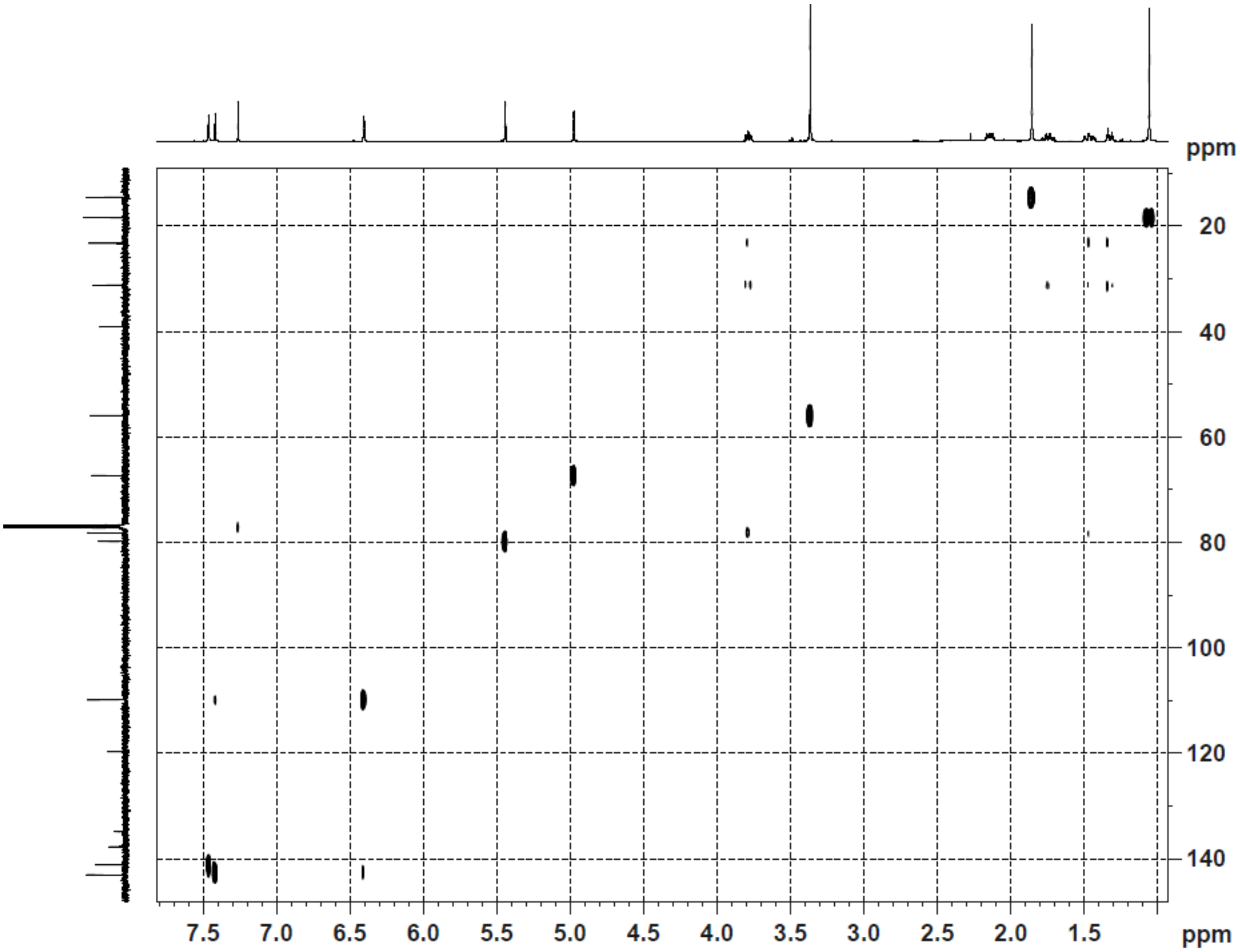

Figure S40 HSQC ($CDCl_3$) spectrum of compound **5**

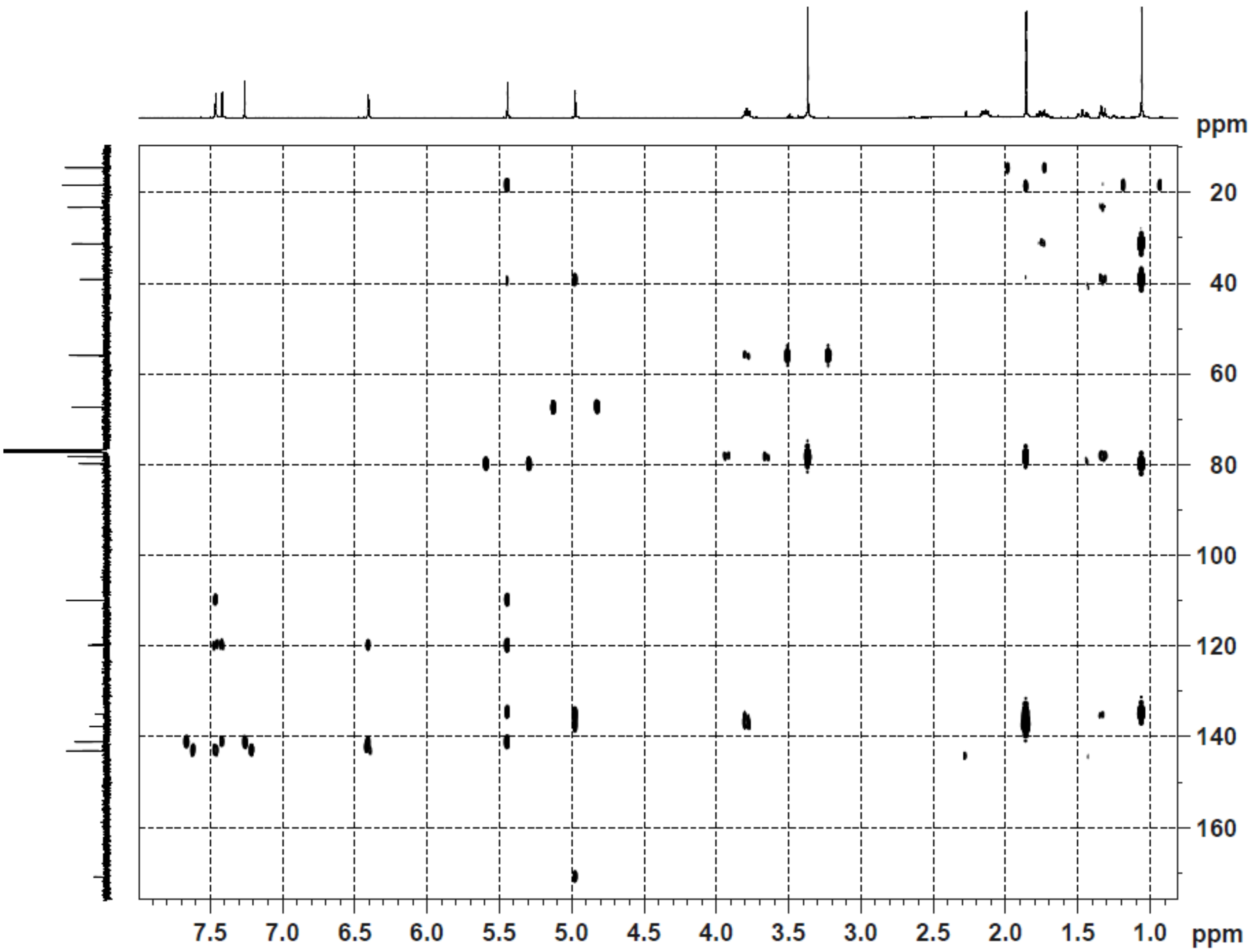

Figure S41 HMBC ($CDCl_3$) spectrum of compound **5**

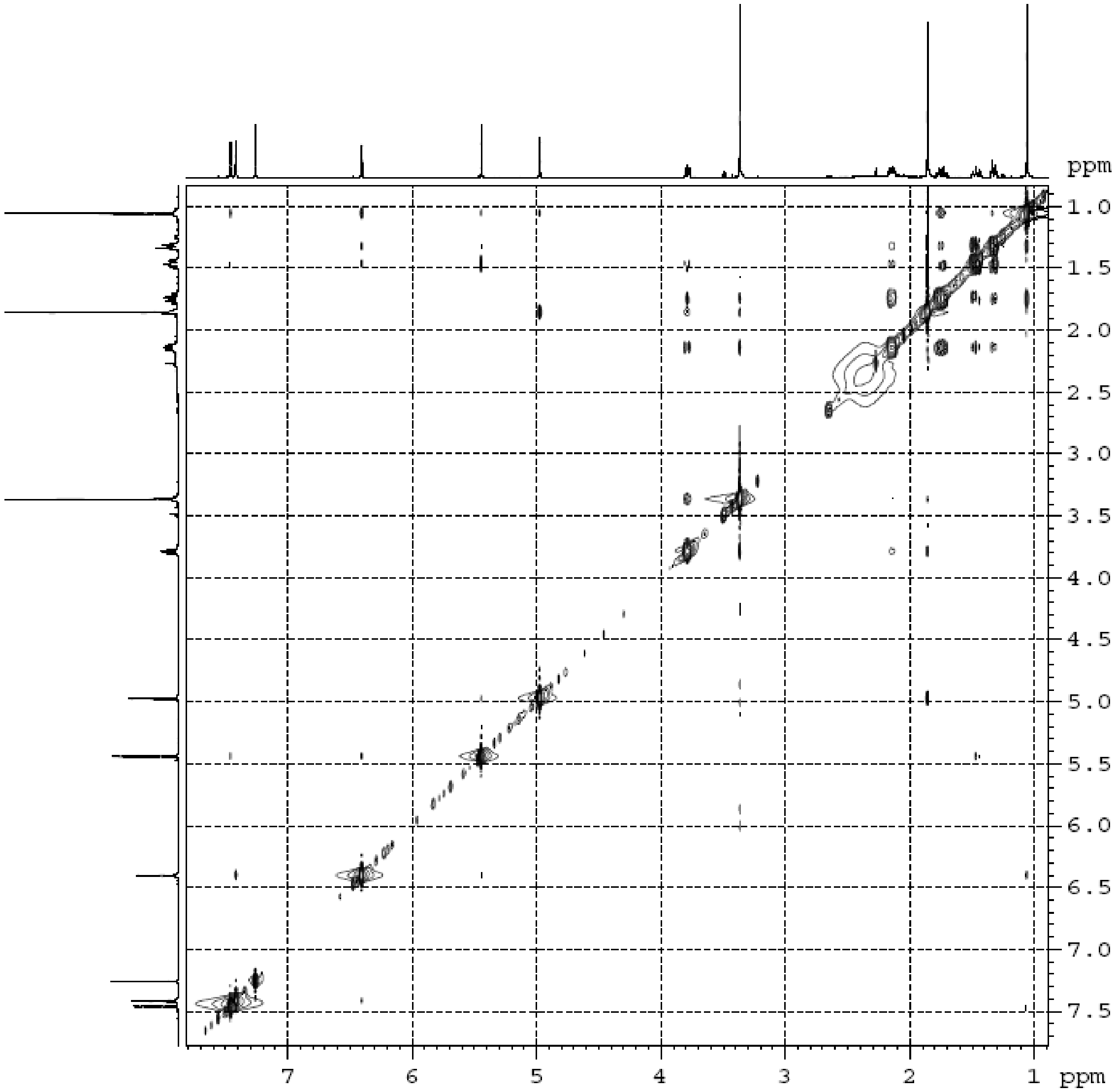

Figure S42 NOSEY ($CDCl_3$) spectrum of compound **5**

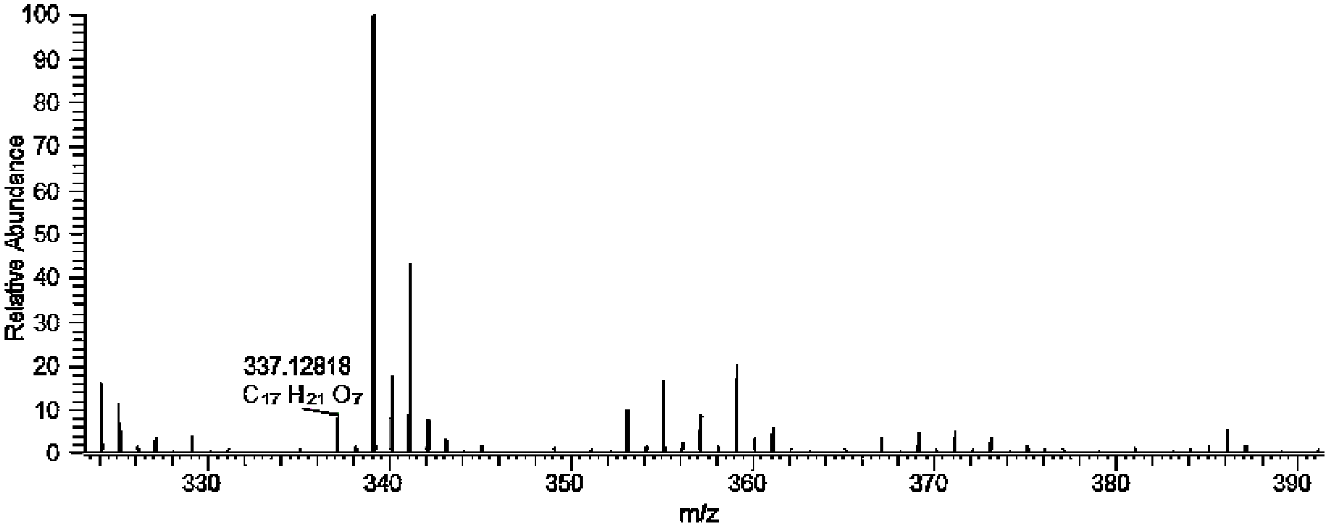

Figure S43 HRESI-Orbitrap-MS spectrum of compound **5**

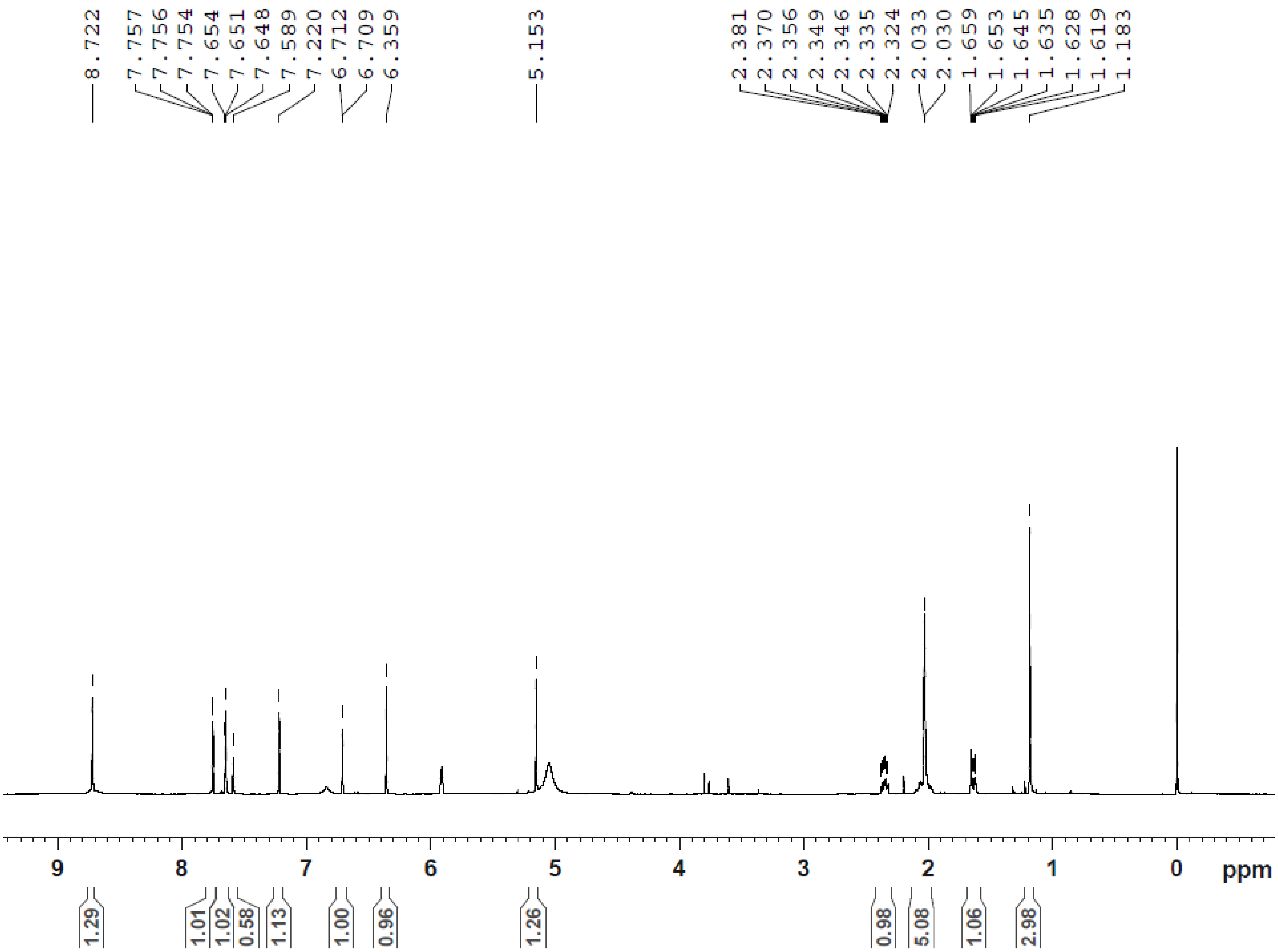

Figure S44 ^{1}H NMR (500 MHz, C_5D_5N) spectrum of compound **6**

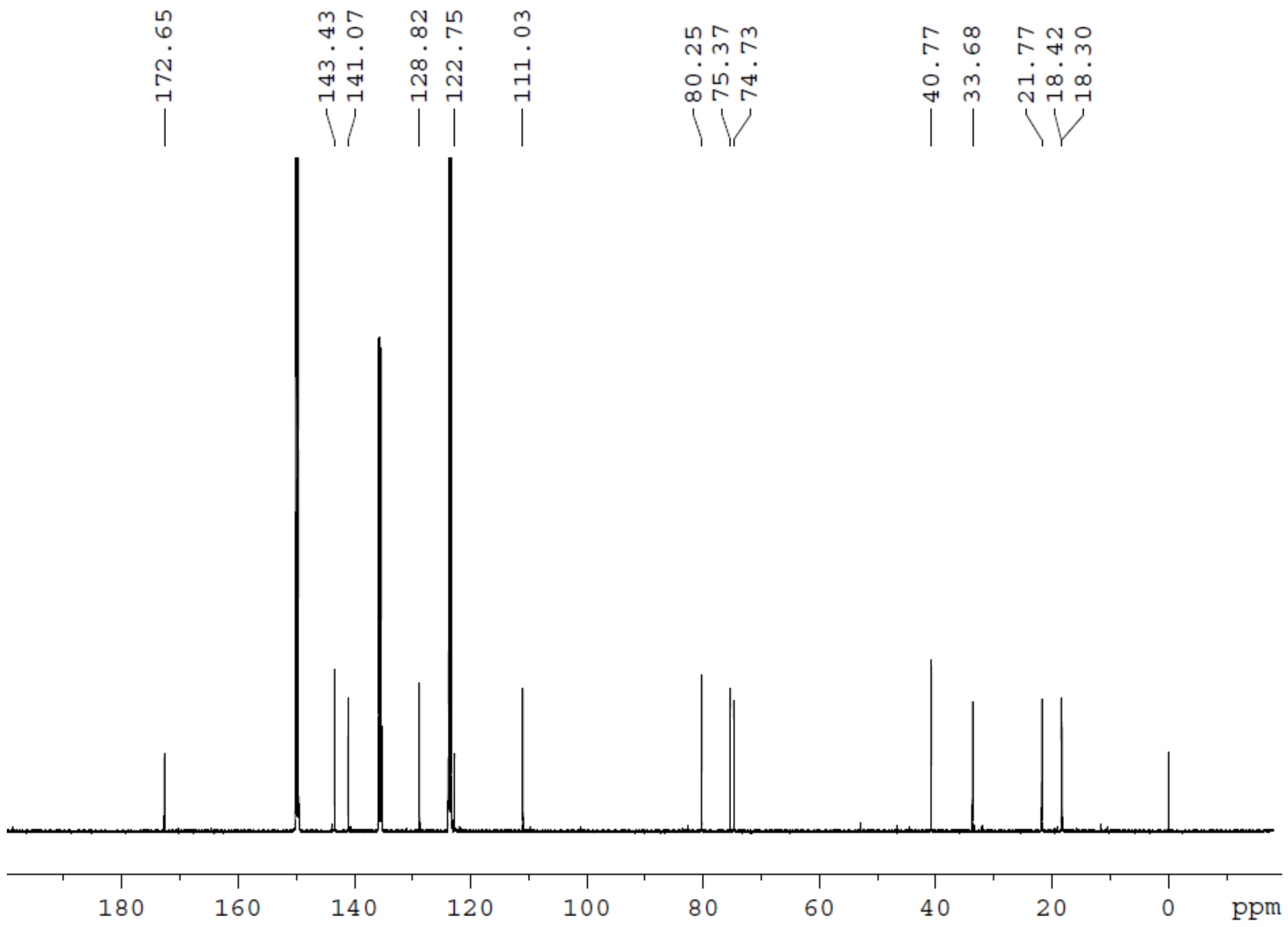

Figure S45 ^{13}C NMR (125 MHz, C_5D_5N) spectrum of compound **6**

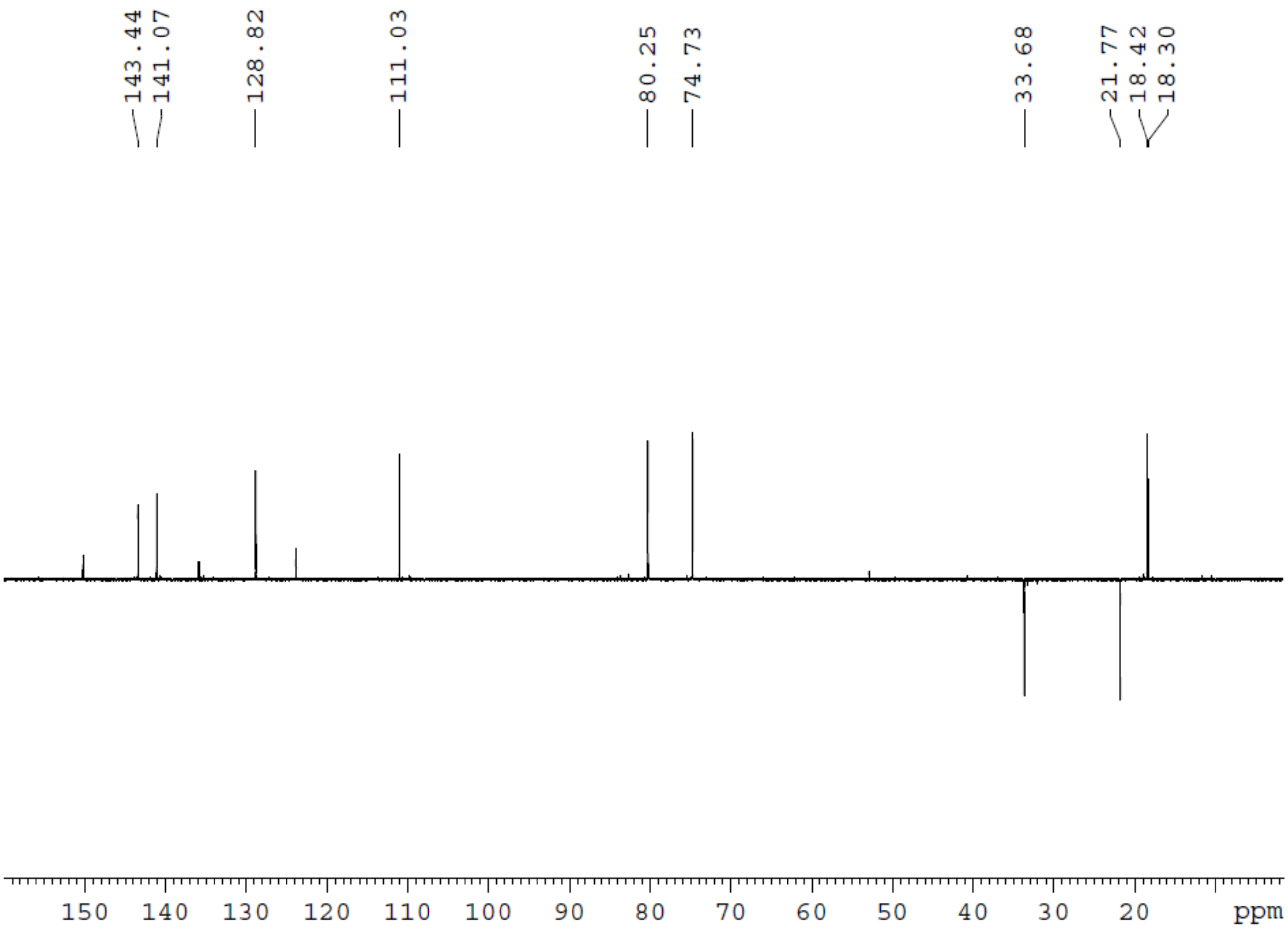

Figure S46 DEPT 135 (C_5D_5N) spectrum of compound **6**

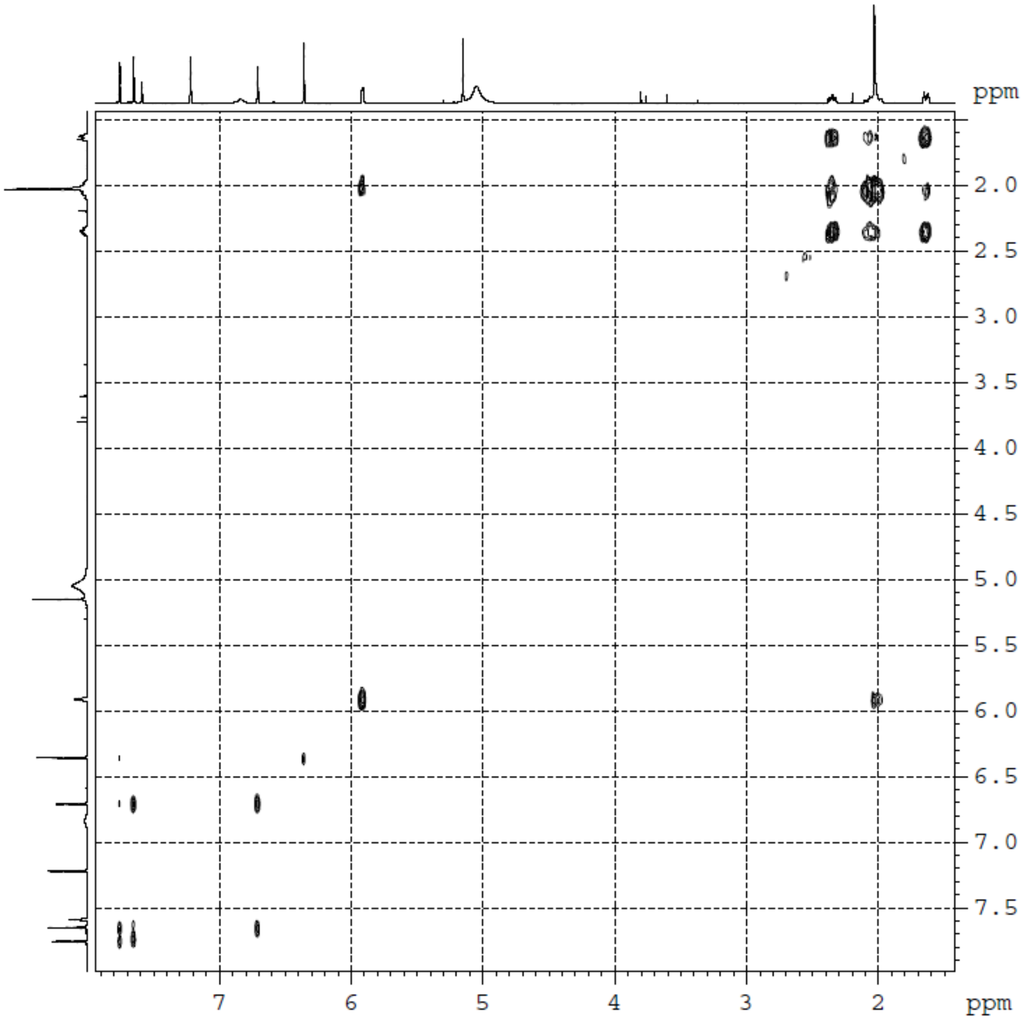

Figure S47 ^{1}H ^{1}H COSY (C_5D_5N) spectrum of compound **6**

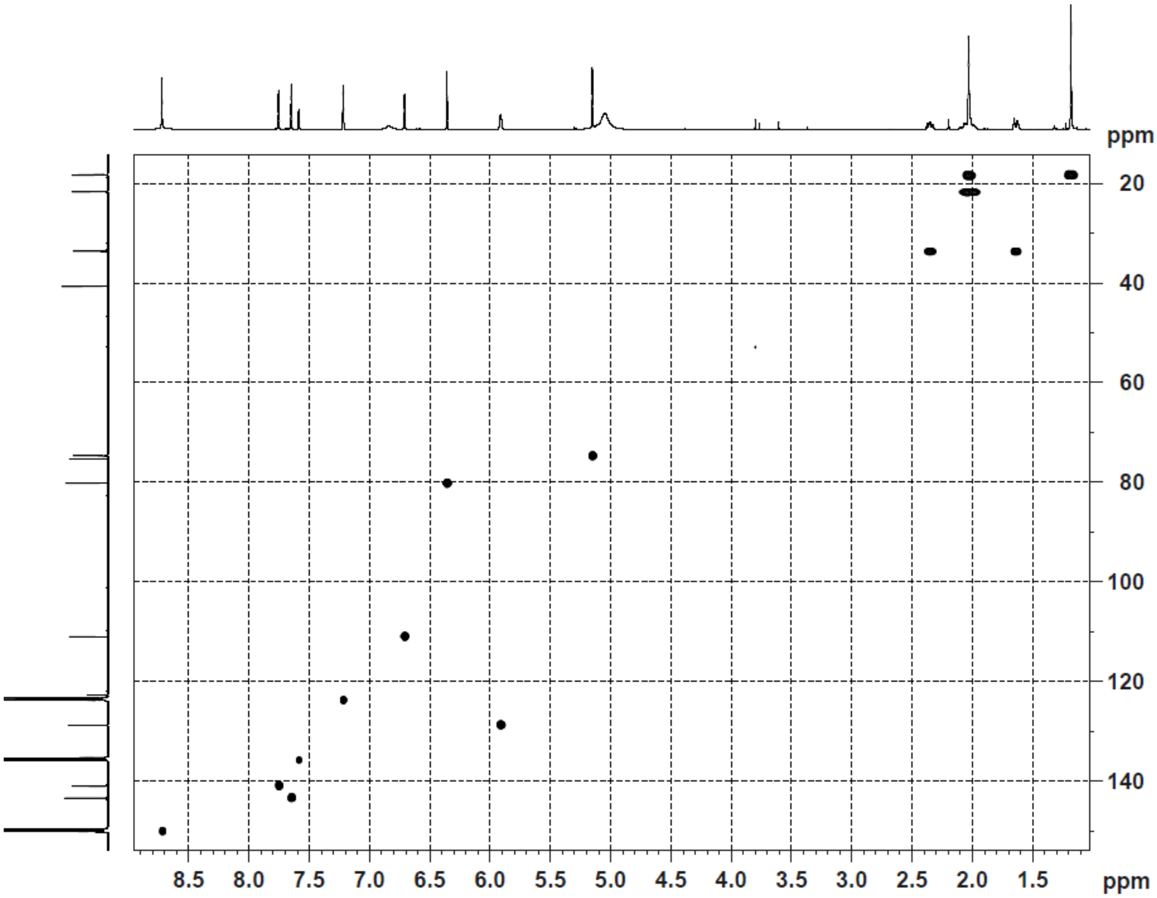

Figure S48 HSQC (C_5D_5N) spectrum of compound **6**

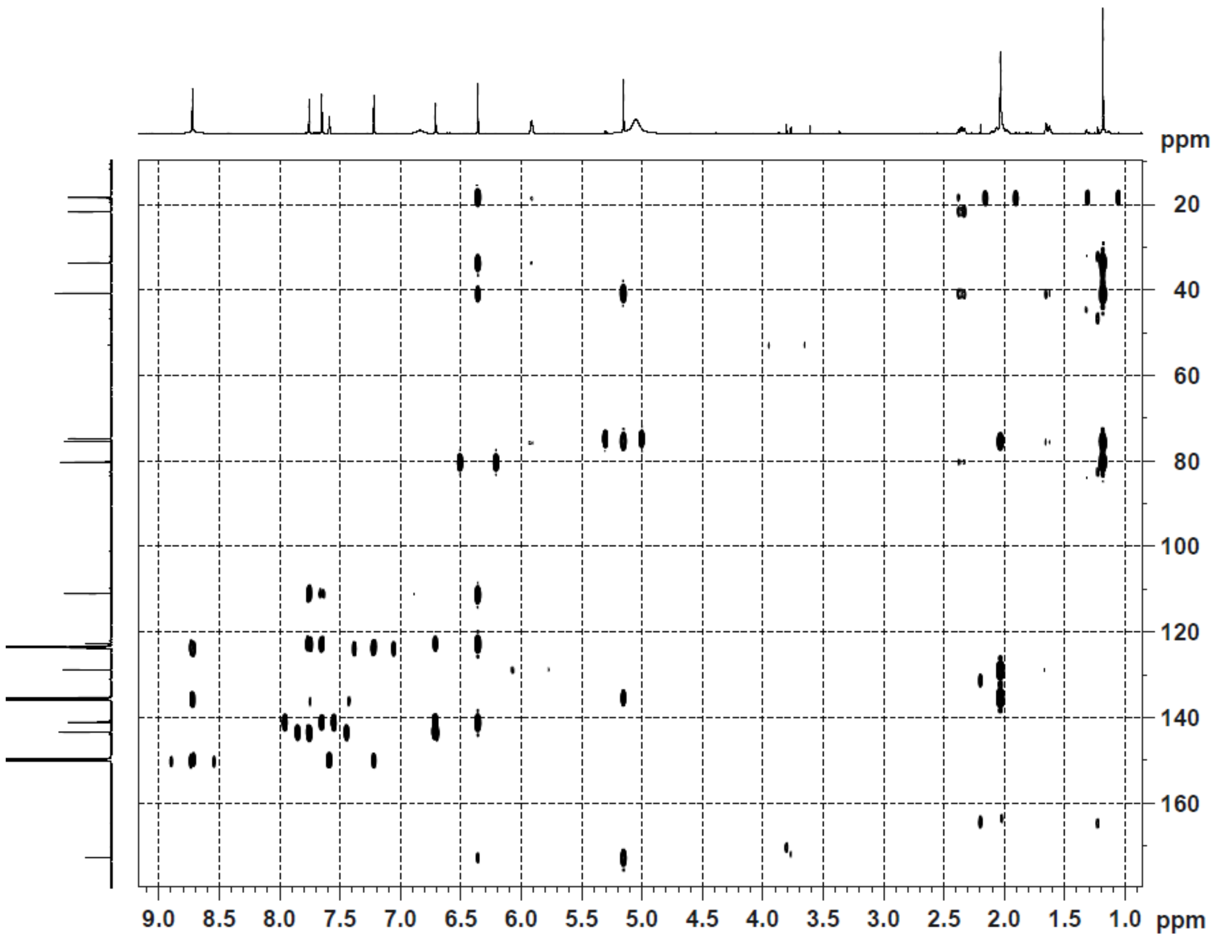

Figure S49 HMBC (C_5D_5N) spectrum of compound **6**

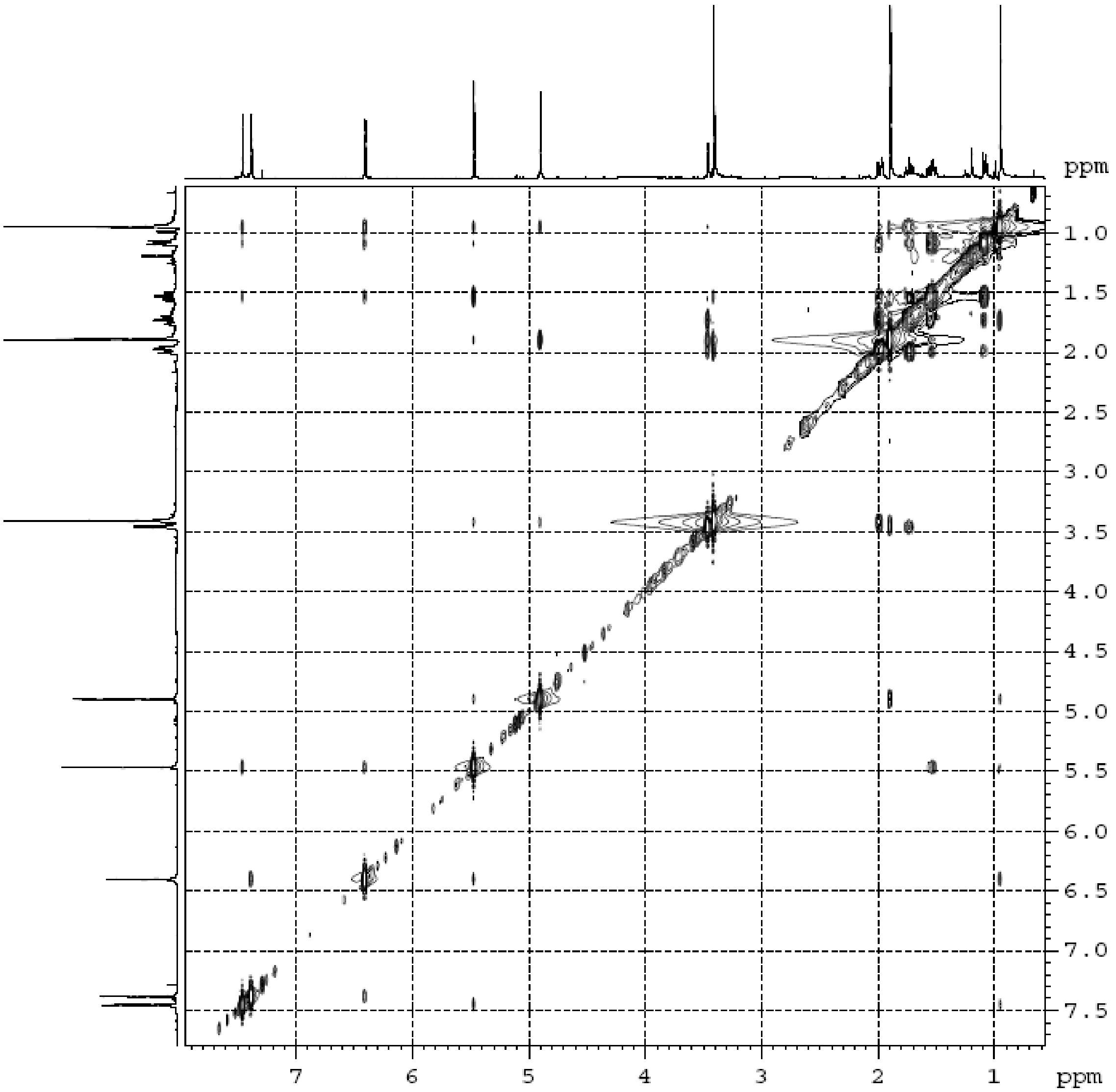

Figure S50 NOSEY (C_5D_5N) spectrum of compound **6**

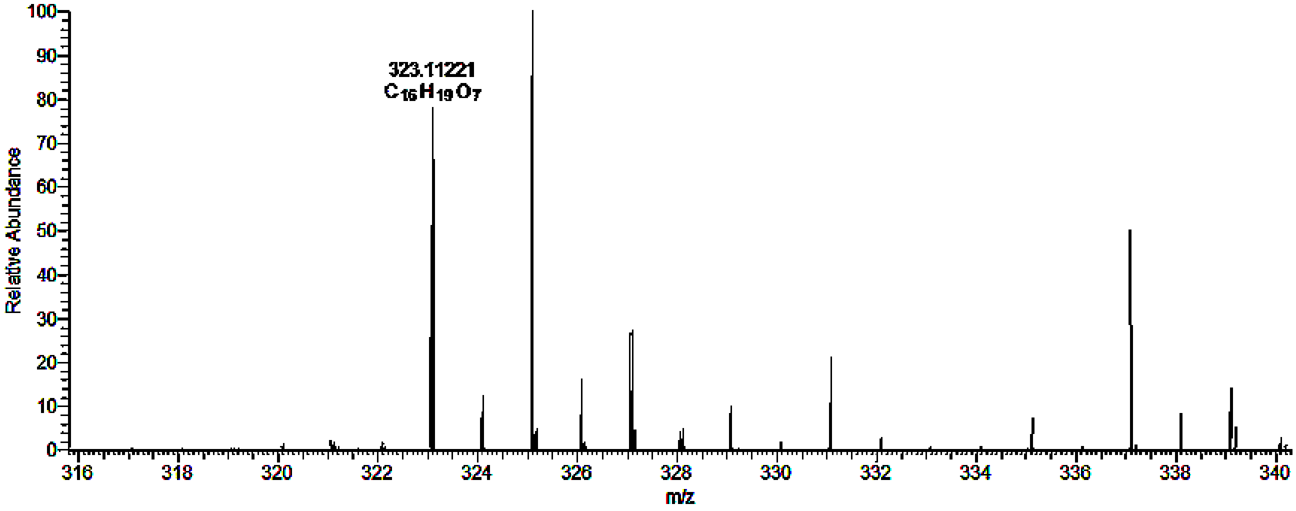

Figure S51 HRESI-Orbitrap-MS spectrum of compound **6**

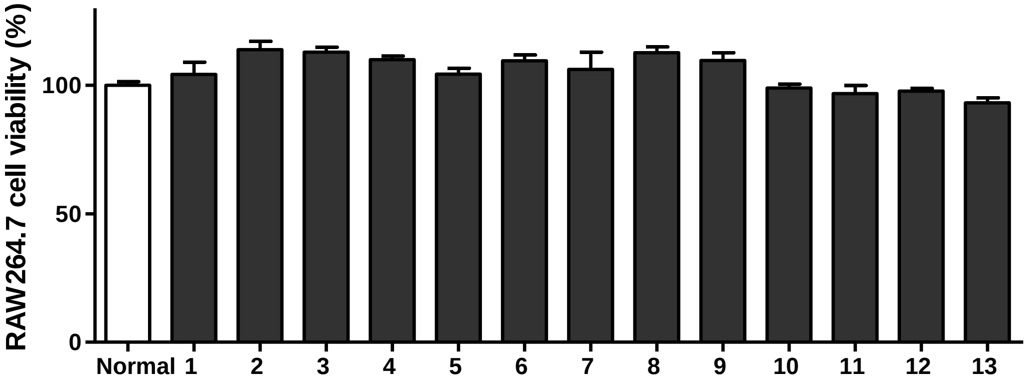

Figure S52 MTT analysis of **1–13** obtained from *Cortex Dictamni* on RAW264.7 cells

Values represent the mean ± SD of three determinations. *P < 0.05; **P < 0.01; ***P < 0.001 (Differences between compound-treated group and normal group). N = 4. Final concentrations were 20 μM for **1–13**, respectively.

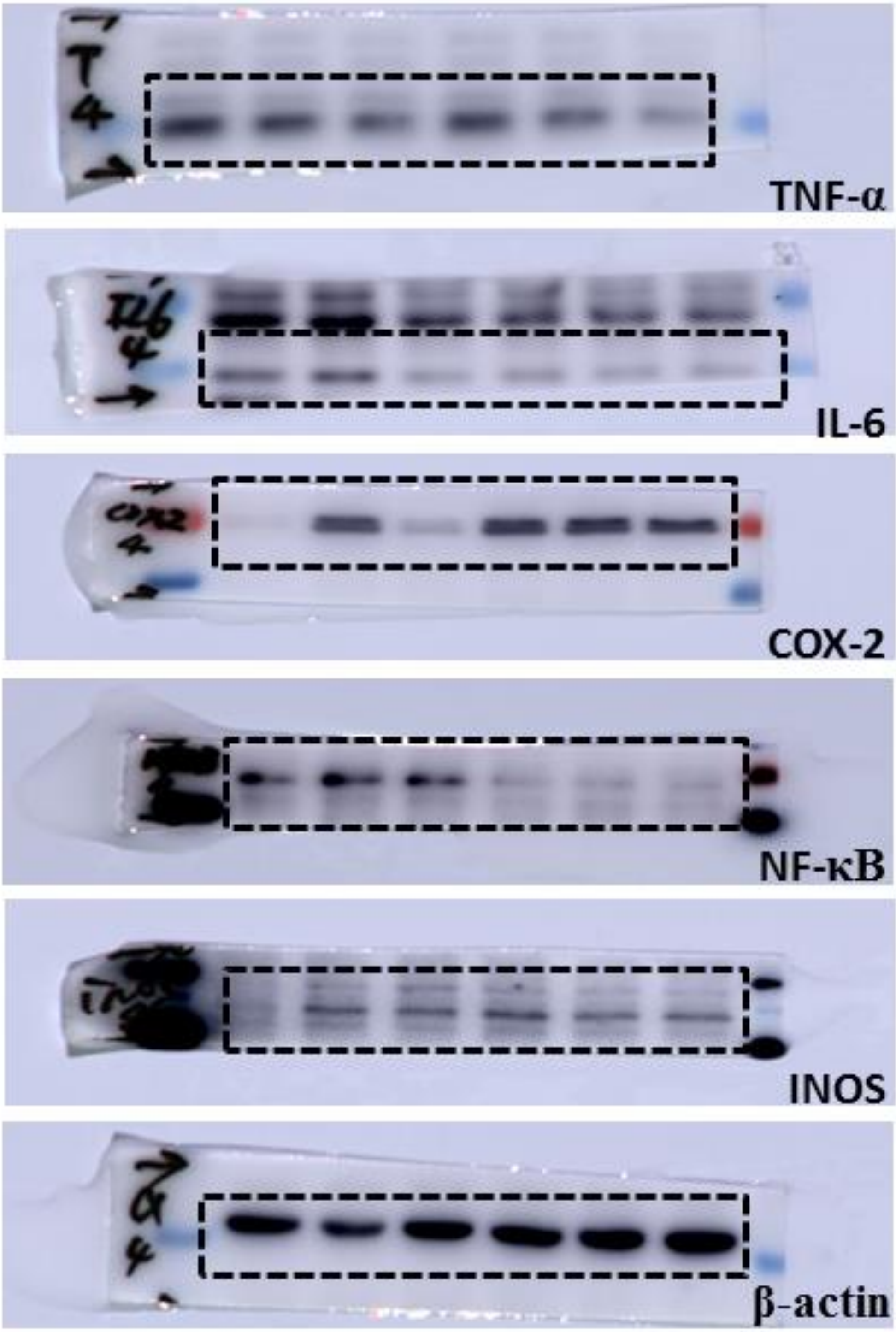

Figure S53 Raw quantification data for figure 6

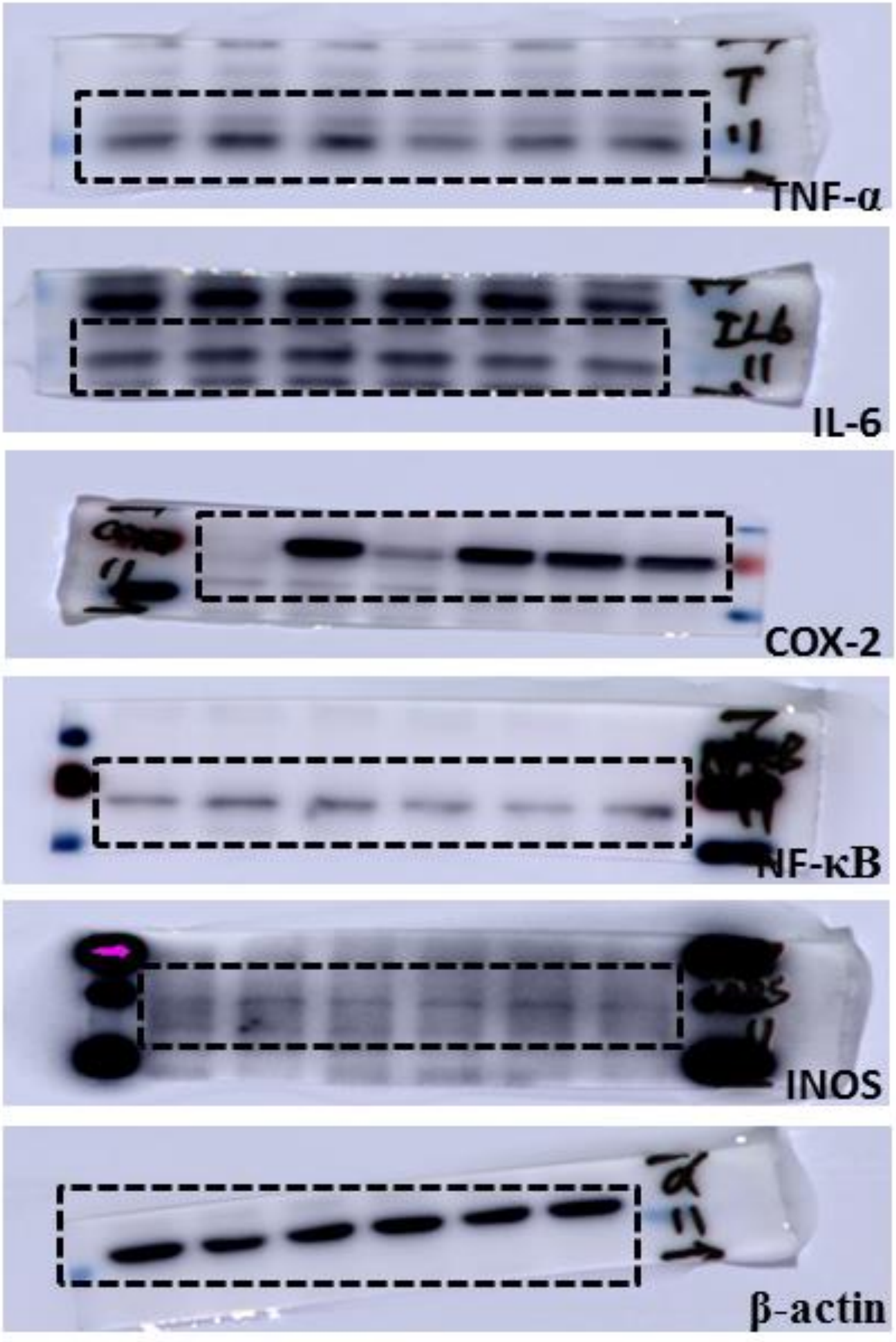

Figure S54 Raw quantification data for figure 7

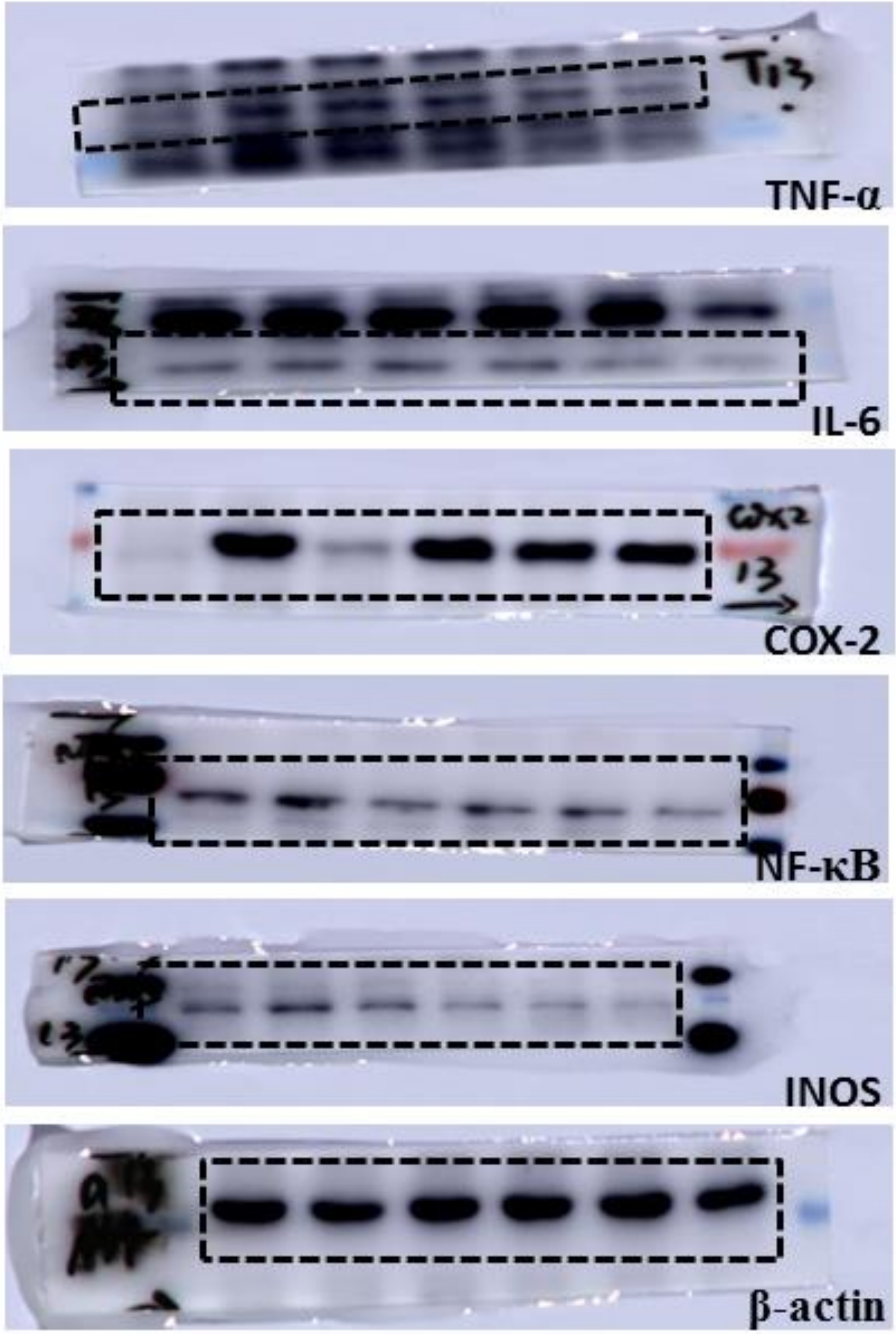

Figure S55 Raw quantification data for figure 8
